# The Hippo/YAP pathway interacts with EGFR signaling and HPV oncoproteins to regulate cervical cancer progression

**DOI:** 10.15252/emmm.201404976

**Published:** 2015-09-28

**Authors:** Chunbo He, Dagan Mao, Guohua Hua, Xiangmin Lv, Xingcheng Chen, Peter C Angeletti, Jixin Dong, Steven W Remmenga, Kerry J Rodabaugh, Jin Zhou, Paul F Lambert, Peixin Yang, John S Davis, Cheng Wang

**Affiliations:** 1 Olson Center for Women’s Health, Department of Obstetrics & Gynecology, University of Nebraska Medical CenterOmaha, NE, USA; 2 College of Animal Science and Technology, Huazhong Agricultural UniversityWuhan, China; 3 College of Animal Science and Technology, Nanjing Agricultural UniversityNanjing, China; 4 Fred & Pamela Buffett Cancer Center, University of Nebraska Medical CenterOmaha, NE, USA; 5 Nebraska Center for Virology, School of Biological Sciences, University of Nebraska-LincolnLincoln, NE, USA; 6 Department of Obstetrics and Gynecology, Urumqi General Hospital of Lanzhou Military RegionUrumqi, China; 7 Department of Oncology, McArdle Laboratory for Cancer Research, University of Wisconsin School of Medicine and Public HealthMadison, WI, USA; 8 Department of Obstetrics, Gynecology & Reproductive Sciences, University of Maryland School of MedicineBaltimore, MD, USA; 9 Omaha Veterans Affairs Medical CenterOmaha, NE, USA

**Keywords:** cervical cancer, EGFR, Hippo, HPV, YAP

## Abstract

The Hippo signaling pathway controls organ size and tumorigenesis through a kinase cascade that inactivates Yes-associated protein (YAP). Here, we show that YAP plays a central role in controlling the progression of cervical cancer. Our results suggest that YAP expression is associated with a poor prognosis for cervical cancer. TGF-α and amphiregulin (AREG), via EGFR, inhibit the Hippo signaling pathway and activate YAP to induce cervical cancer cell proliferation and migration. Activated YAP allows for up-regulation of TGF-α, AREG, and EGFR, forming a positive signaling loop to drive cervical cancer cell proliferation. HPV E6 protein, a major etiological molecule of cervical cancer, maintains high YAP protein levels in cervical cancer cells by preventing proteasome-dependent YAP degradation to drive cervical cancer cell proliferation. Results from human cervical cancer genomic databases and an accepted transgenic mouse model strongly support the clinical relevance of the discovered feed-forward signaling loop. Our study indicates that combined targeting of the Hippo and the ERBB signaling pathways represents a novel therapeutic strategy for prevention and treatment of cervical cancer.

## Introduction

Cervical cancer is the second most commonly diagnosed cancer and the fourth leading cause of cancer death in women worldwide (Jemal *et al*, 2011). The estimate from the International Agency for Research on Cancer (IARC) predicted that 528,000 women would be diagnosed with cervical cancer and 266,000 deaths would result from this disease in 2012 (Ferlay *et al*, 2013). Cervical cancer affects women when they are still young and has devastating effects with a very high human, social, and economic cost. Human papillomavirus (HPV) infection is detected in 99.7% of cervical cancer patients and is believed to be the major risk factor for cervical cancer (Jemal *et al*, 2011; Ferlay *et al*, 2013). However, epidemiological studies have shown that although the estimated lifetime risk of HPV infection is more than 75% (Koutsky, 1997), the lifetime risk of developing cervical cancer is only around 0.7% (Siegel *et al*, 2014). It is clear that HPV alone is insufficient for malignant transformation and uncontrolled growth of cervical epithelium (Mahdavi & Monk, 2005; Chan & Berek, 2007). The exact molecular mechanisms underlying cervical cancer initiation and progression are largely unknown.

The Hippo signaling pathway, originally discovered in *Drosophila*, is an evolutionarily conserved pathway that controls organ size by regulating cell proliferation in a diverse number of species. The core Hippo pathway, consisting of a kinase cascade and associated co-activators and scaffold proteins, has been well established in both *Drosophila* and mammals (Pan, 2010; Mo *et al*, 2014). In the mammalian Hippo pathway, macrophage stimulating 1/2 kinase (MST1/2, called Hippo in *Drosophila*), in complex with Salvador homolog 1 (Sav1), phosphorylates the large tumor suppressor kinase 1/2 (LATS1/2) and a regulatory protein, Mps one binder kinase activator-like 1A (MOB1). Phosphorylated LATS1/2 and MOB1 form an interaction complex that leads to activation of LATS1/2. Activated LATS1/2, in turn, phosphorylates the growth-promoting transcriptional co-activator Yes-associated protein (YAP1, or more commonly YAP). Phosphorylation of YAP leads to its cytoplasmic retention and/or degradation, depending on the sites of phosphorylation (Dong *et al*, 2007; Zhao *et al*, 2007; Pan, 2010; Mo *et al*, 2014). Conversely, loss of the Hippo signaling can lead to organ overgrowth and induce tumors in model organisms (Dong *et al*, 2007; Lee *et al*, 2010). Dysregulation of the Hippo pathway occurs in a broad range of human carcinomas, including lung, colorectal, breast, ovarian, pancreatic, gastric, and liver cancer (Hall *et al*, 2010; Lee *et al*, 2010; Wang *et al*, 2010, 2012b; Kang *et al*, 2011; Zhang *et al*, 2011; Avruch *et al*, 2012; Hergovich, 2012; He *et al*, 2015). However, whether the Hippo pathway plays a role in the progression of cervical cancer development is currently unknown.

Yes-associated protein is the major downstream effector of the Hippo pathway (Pan, 2010). Recent studies suggest that expression and function of YAP in cancer are cell type and/or cellular context dependent. Several studies define YAP as an oncogene. For example, the amplification of the YAP gene locus at 11q22 is found in several types of cancers (Snijders *et al*, 2005; Overholtzer *et al*, 2006; Zender *et al*, 2006; Fernandez *et al*, 2009; Kang *et al*, 2011; Muramatsu *et al*, 2011). Overexpression and nuclear localization of the YAP protein has been noted in colon, liver, lung, ovarian, and prostate cancers (Snijders *et al*, 2005; Zhao *et al*, 2007; Steinhardt *et al*, 2008; Zhang *et al*, 2011; Yu & Guan, 2013; He *et al*, 2015). Overexpression of YAP induced oncogenic transformation of an immortalized breast epithelial cell line, MCF10A (Overholtzer *et al*, 2006), and significantly stimulated granulosa cell tumor cell proliferation (Fu *et al*, 2014). On the other hand, YAP was reported to be a tumor suppressor in certain cell types. YAP has been shown to enhance p73-dependent cell death during cisplatin-induced DNA damage (Strano *et al*, 2005). In a subset of breast cancers, YAP protein expression was significantly decreased due to loss of heterozygosity. Additionally, shRNA knockdown of YAP increased anchorage-independent growth, migration, and invasiveness of breast cancer cells and enhanced tumor growth in nude mice (Yuan *et al*, 2008). However, the precise mechanism for the expression and function of YAP in cervical cancer cell remains undefined.

In the present study, we sought to determine the expression of YAP in human cervical cancer tissues, and to examine the role of the Hippo pathway in the progression of cervical cancer. We found that YAP is overexpressed in cervical cancer tissue. We also found that the Hippo signaling pathway, through interaction with the EGFR signaling pathway, regulates progression of cervical cancer via an autocrine loop involving EGF-like ligands, EGFR, and the Hippo pathway. Importantly, we discovered that the HPV E6 protein stabilizes the YAP protein to maintain its action on the progression of cervical cancer.

## Results

### YAP expression during cervical cancer progression

**Figure 1:**
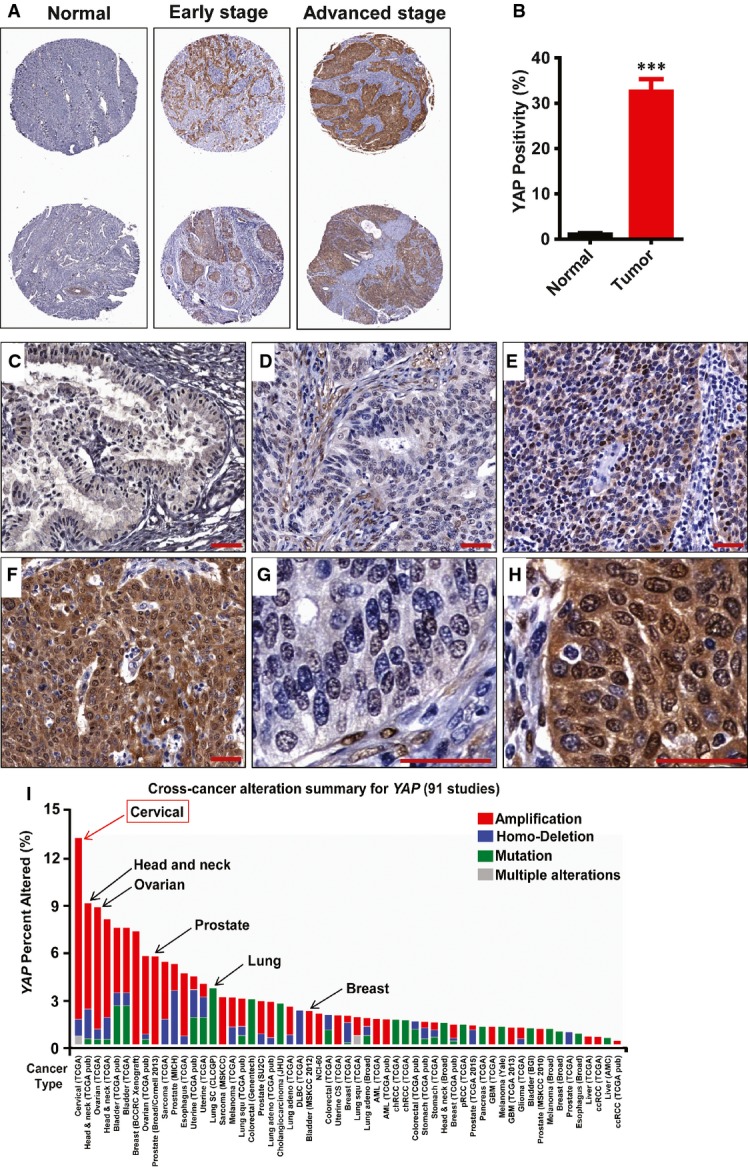
YAP expression in normal cervical tissues and cervical cancers A Representative images show expression of YAP in normal cervical tissues, early-stage cervical cancer (stage I & II), and advanced-stage cervical cancer (stage III & IV). Note the significant increase in YAP immunosignals (brown staining) in the advanced-stage tumors. Tissue core size: 1.5 mm.

Compared with normal control tissues, both the positivity and intensity of the YAP immunosignal, which indicate YAP-positive cells and YAP protein levels, respectively, were significantly higher in the cervical cancer tissues (Fig1A and B, Tables1 and 2). We also found that YAP expression and localization were associated with tumor stage. Compared with tumor tissue from patients with early-stage disease (Fig1D, E and G), tissue from patients with advanced-stage cervical cancer had significantly higher levels of YAP protein, which was localized mainly to the nucleus of tumor cells (Fig1F and H).

B Positivity (percentage of positively stained cells relative to the total number of cells in the tissue section) of YAP immunosignals in the normal and cancerous tissues. Data were analyzed with unpaired *t*-test in GraphPad Prism 5 with Welch’s correction. Bars represent means ± SEM (*n *=* *10 for normal tissues; *n *=* *69 for tumor tissues; ***P <* *0.0001).

C A representative image showing the expression of YAP (brown staining) in normal cervical tissue. Scale bar = 20 μm.

D–F Representative images showing the expression of YAP in (D) early-stage cervical cancer tissue (stage Ib, T1N0M0); (E) medium-stage cervical cancer tissue (stage IIb, T2N0M0); and (F) advanced-stage cervical cancer tissue (stage IIIb, T3N1M0). Scale bar = 20 μm.

G, H High-resolution images showing the cellular location of YAP in (G) early-stage cervical cancer tissue and (H) advanced-stage cervical cancer tissue. Scale bar = 20 μm.

I Multidimensional cancer genomics data analysis results showing cross-cancer YAP gene alteration. The histogram showed the frequencies of YAP gene mutation, deletion, and amplification across cancers. Data were extracted from TCGA database and analyzed using cBioPortal online analyzing tools. The results indicate that the highest alteration frequency of YAP gene occurs in cervical cancer.

**Table 1:** Positivity^a^ of YAP immunosignal in normal cervical tissues and cervical tumors

Signal Intensity	Tumor (*n* = 69)	Normal (*n* = 10)
Negative	2.9% (2/69)	60% (6/10)
Weak	5.8% (4/69)	40% (4/10)
Moderate	13.0% (9/69)	0
Strong	78.3% (54/69)	0

a Positivity: the ratio of immunosignal-positive cell number to the total cell number. Signal intensity was classified as follows: negative, positivity < 1%; weak, 1% < positivity < 10%; moderate, 10% < positivity < 20%; and strong, positivity > 20%.

**Table 2:** Correlation between YAP expression and the clinicopathologic determinants in cervical cancer

Feature	IP (*P*)	Positivity (*P*)
Tumor vs. Normal	0.021	< 0.0001
Grade	0.512	0.645
Stage	< 0.0001	0.444
T	0.0008	0.647
N	0.002	0.312
Age	0.080	0.362

IP = total intensity of positive signal.

Positivity: the ratio of immunosignal-positive cell number to the total cell number.

T: primary tumor: T1—tumor invades submucosa; T2—tumor invades muscularis propria; T3—tumor invades through muscularis propria into subserosa or into non-peritonealized pericolic or perirectal tissues; T4—tumor directly invades other organs or structures and/or perforate visceral peritoneum.

N: regional lymph nodes: N0—no regional lymph node metastasis; N1—metastasis in 1–3 regional lymph nodes; N2—metastasis in 4 or more regional lymph nodes.

M: distant metastasis: M0—no distant metastasis; M1—distant metastasis.

The relationships between YAP protein levels and clinical histopathological parameters were also analyzed in this study. As described in Table2, YAP positivity was greater in cervical cancer tissues compared to normal tissues, but the number of YAP-positive cells in cervical cancer did not vary with age, grade, stage, primary tumor, and regional lymph node status. Notably, the intensity of YAP immunosignal (expression levels) was significantly correlated with the FIGO stage, primary tumor, and regional lymph node status, but not the tumor grade (Table2).

Western blot analyses showed that YAP was differentially expressed in normal and cancerous cervical cell lines (Appendix Fig S1A). Consistent with IHC results, YAP was highly expressed in cervical cancer cell lines (ME180, HT3, and HeLa), while in Ect1 cells, an immortalized epithelial cell line derived from the ectocervical epithelium, YAP protein was expressed at low levels and was highly phosphorylated (Appendix Fig S1A).

**Figure EV1:**
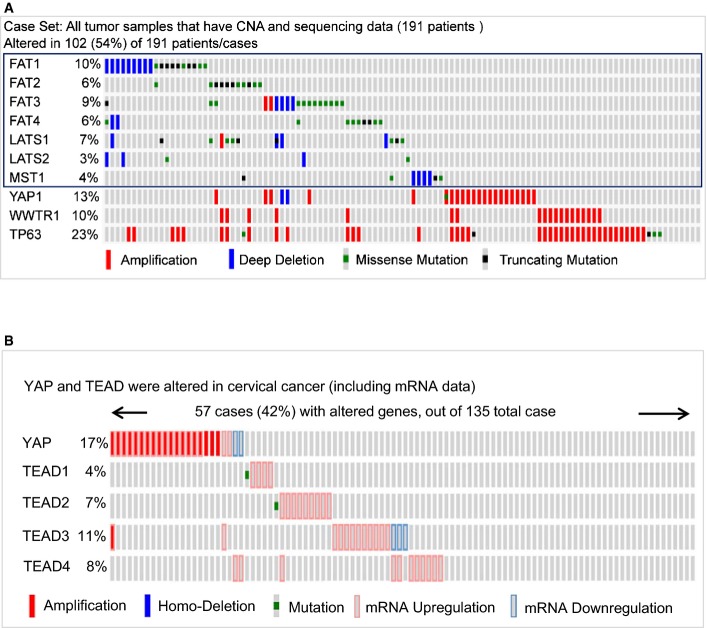
Multidimensional cancer genomics data analysis showing alteration of the major genes involved in the Hippo/YAP pathway in cervical cancer Alteration frequencies of major genes involved in the Hippo pathway. Genes in the blue box are upstream genes of the Hippo tumor suppressor pathway. Note the frequent deletion and mutation of these genes in cervical cancer. YAP and TAZ (WWTR1) genes are frequently amplified in cervical cancer (*n *=* *191). TP63 are a known cervical cancer biomarker and are frequently amplified in cervical cancer.

To confirm that YAP plays a role in human cervical cancer progression, we analyzed *YAP* gene alteration using data extracted from The Cancer Genomic Atlas (TCGA) database and the cBioPortal online analyzing tool (the cBioPortal for Cancer Genomics) (Cerami *et al*, 2012; Gao *et al*, 2013). The cross-cancer *YAP* alteration analysis shows that *YAP* is frequently altered in different types of cancers (Fig1I). Interestingly, among 36 examined cancer types or subtypes (from a total of 90 studies), the cervical cancer has the highest frequency of *YAP* gene amplification (Fig1I). Intriguingly, Analysis of the cervical cancer patient sample from the TCGA datasets indicated that upstream genes involved in the Hippo tumor-suppressing pathway are frequently deleted and mutated, while the effectors, *YAP* and *WWTR1 (TAZ)* genes, are frequently amplified in 191 cervical cancer cases (Fig EV1A). Further analysis using 135 cervical cancer genome sequencing data from TCGA datasets indicates that *YAP* gene is altered in 17% examined cases (FigEV1). TEADs are the major mediators of YAP transcriptional activities. In the examined cervical cancer patient samples, 42% cases have alterations in at least one of the genes in YAP-TEAD complex (FigEV1B). Moreover, network analysis showed that almost all genes that interacted with YAP, including other YAP-associated transcriptional factors such as ERBB4, Runx1, and Runx2, are up-regulated in various degrees in examined cervical cancer cases (Appendix Fig S2).

A visual summary of the different mechanisms of YAP1 and TEAD alteration across a set of cervical cancer samples based on a query of the five genes, *YAP, TEAD1, TEAD2, TEAD3*, and *TEAD4*. Each row represents a gene, and each column represents a tumor sample (*n *=* *135). The YAP and TEADs gene alteration analyses were performed using online datasets and data mining tools (the cBioPortal for Cancer Genomics and the datasets from the TCGA Research Network).

### YAP promotes proliferation and migration of cervical cancer cells *in vitro*

**Figure 2:**
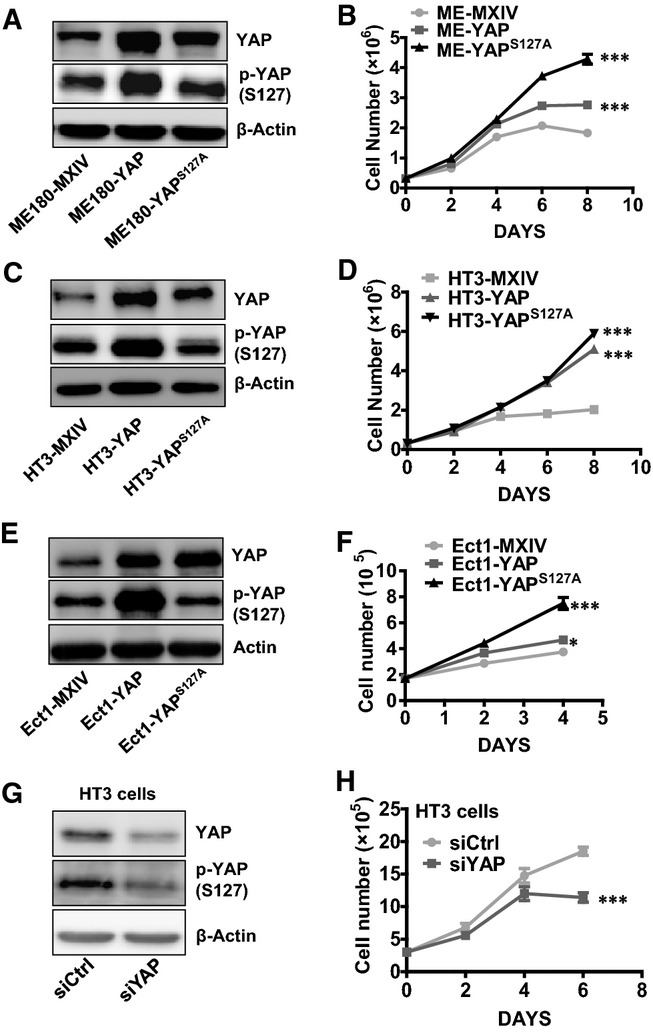
Effect of YAP on the proliferation of normal and cancerous cervical cells A, C, E Western blot analysis showing levels of YAP and phosphorylated YAP in ME180 cell lines [ME180-MXIV (control), ME180-YAP, and ME180-YAP^S^^127A^ cells] (A); HT3 cell lines [HT3-MXIV (control), HT3-YAP, and HT3-YAP^S^^127A^ cells] (C); and Ect1 cell lines [Ect1-MXIV (control), Ect1-YAP, and Ect1-YAP^S^^127A^ cells] (E). β-Actin was used as a protein loading control.

Since YAP is overexpressed in cervical cancer, we used ME180 (HPV positive) and HT3 (HPV negative) cervical cancer cell lines to clarify the role of YAP in cervical cancer cell proliferation. We established six cell lines with differential YAP protein levels and activities: ME180-YAP^S127A^ and HT3-YAP^S127A^ cell lines expressing constitutively activated YAP; ME180-YAP and HT3-YAP overexpressing wild-type YAP; and ME180-MXIV and HT3-MXIV cells transfected with control vectors (MXIV). As expected, YAP was overexpressed in ME180-YAP, HT3-YAP, ME180-YAP^S127A^, and HT3-YAP^S127A^ cells (Fig2A and C, Appendix Fig S3A and B). An increase in phosphorylated YAP was observed in the ME180-YAP and HT3-YAP cells, but not in ME180-YAP^S127A^ and HT3-YAP^S127A^ cells, consistent with the mutation of serine 127 to alanine (Fig2A and C, Appendix Fig S3A and B), which results in constitutive YAP activity (Pan, 2010). We observed that in the presence of complete medium (10% serum for HT3, 2.5% serum for ME180), the growth rate of the ME180 and HT3 cell lines was similar prior to reaching confluence. After reaching confluence (> 4 days after cell plating), cells in the control groups almost stopped proliferating. However, cells overexpressing YAP or YAP^S127A^ continued to proliferate (Fig2B and D), with the highest growth rates observed in ME180-YAP^S127A^ and HT3-YAP^S127A^ cells. Interestingly, when examined under serum-reduced conditions (1% FBS), the growth rate of the ME180-YAP^S127A^ cells was significantly higher than that of the ME180-YAP cells, while growth rate of the ME180-YAP cells was significantly higher than that of ME180-MXIV cells, even before the cell cultures reach confluence (Appendix Fig S3C). We then analyzed cell cycle progression in these cell lines after they reached confluence. The results showed that overexpression or constitutive activation of YAP increased the percentage of cells in S and G2/M phases, and reduced the proportion of cells in G1 phase in both ME180 and HT3 cervical cancer cells (Appendix Fig S4).

B, D, F Growth curves of YAP-overexpressed ME180 cell lines [ME180-MXIV (control), ME180-YAP, and ME180-YAP^S^^127A^ cells] (B); HT3 cell lines [HT3-MXIV (control), HT3-YAP, and HT3-YAP^S^^127A^ cells] (D); and Ect1 cell lines [Ect1-MXIV (control), Ect1-YAP, and Ect1-YAP^S^^127A^ cells] (F). Each point represents the mean ± SEM (*n *=* *4). ***P <* *0.0001, ME180-MXIV vs. ME180-YAP cells and ME180-MXIV vs. ME180-YAP^S^^127A^ cells on day 8. ***P <* *0.0001, HT3-MXIV vs. HT3-YAP cells and HT3-MXIV vs. HT3-YAP^S^^127A^ cells on day 8. ***P <* *0.0001, Ect1-MXIV vs. Ect1-Ect1-YAP^S^^127A^ cells on day 4. *P <* *0.0074, Ect1-MXIV vs. Ect1-YAP cells on day 4.

G Western blot showing YAP levels in non-targeting control siRNA (siCtrl)- and YAP siRNA (siYAP)-transfected HT3 cells.

H Proliferation of HT3 cells treated with control (siCtrl) or YAP siRNA (siYAP). Each point represents the mean ± SEM (*n *=* *5). ***P <* *0.001 compared with siCtrl (siCtrl vs. siYAP, *P *=* *0.0002). Data information: Data in (B), (D), and (F) were analyzed for significance using a one-way ANOVA in GraphPad Prism 5 with Tukey’s *post hoc* test. Data in (H) were analyzed with an unpaired *t*-test in GraphPad Prism 5 with Welch’s correction. Source data are available online for this figure.

The cervical epithelial cell line Ect1/E6E7 was immortalized with HPV16 E6/E7 and is considered to be an immortalized non-tumorigenic ectocervical epithelial cell line (Fichorova *et al*, 1997; Fichorova & Anderson, 1999). We used this cell line to determine whether YAP was able to stimulate the proliferation of immortalized cervical epithelial cells. We established three cell lines based on Ect1/E6E7 cells: Ect1-MXIV (Ect1/E6E7 cells transfected with an empty control vector), Ect1-YAP (Ect1/E6E7 cells overexpressing wild-type YAP), and Ect1-YAP^S127A^ cells (Ect1/E6E7 cells overexpressing constitutively active YAP). Western blot detection of phosphorylated and total YAP levels in these cells showed that YAP was successfully overexpressed in Ect1-YAP and Ect1-YAP^S127A^ cells (Fig2E). Morphologically, Ect1-YAP^S127A^ cells formed many cell plaques, which may be attributed to multilayered cell growth (Appendix Fig S5). As expected, the cell growth curve showed that Ect1-YAP^S127A^ cells proliferated faster and Ect1-MXIV cells proliferated slower than Ect1-YAP cells (Fig2F).

To confirm that YAP plays a role in regulating the proliferation of cervical cancer cells, we used YAP siRNA to knock down YAP protein in ME180 and HT3 cells. Non-targeting control siRNA (siCtrl) was used as a control. Western blot analysis demonstrated that YAP siRNA successfully reduced YAP protein level in ME180 and HT3 cells (Fig2G, Appendix Fig S6A). Knockdown of YAP protein did not affect HT3 cell proliferation until day 4, when the cells achieved a higher density (≥ 90%). After day 4, control HT3 cells treated with non-targeting control siRNA (siCtrl) continued to proliferate, although the growth rate was lower than in low-density cells. However, HT3 cells treated with YAP siRNA stopped growing (Fig2H). Similarly, after day 4, the growth rate of control ME180 cells (siCtrl) was significantly higher than in siYAP treatment ME180 cells (Appendix Fig S6B). This is consistent with our finding that YAP protein level and activity in ME180 cervical cancer cells were associated with cell density. YAP protein in ME180 cells allowed to proliferate to high density in culture was highly phosphorylated (Appendix Fig S1B). Phosphorylation of YAP at serine 127 leads to sequestration of YAP in the cytoplasm, leading to its inactivation (Zender *et al*, 2006; Fernandez *et al*, 2009).

Wound-healing assays showed that, compared to the controls, overexpression or constitutive activation of YAP leads to significant increases in wound closure (*P *<* *0.001), indicating that YAP also plays an important role in the regulation of cervical cancer cell migration (Appendix Fig S7).

### YAP is able to transform cervical epithelial cells and enhances anchorage-independent cervical cancer cell growth

**Figure 3:**
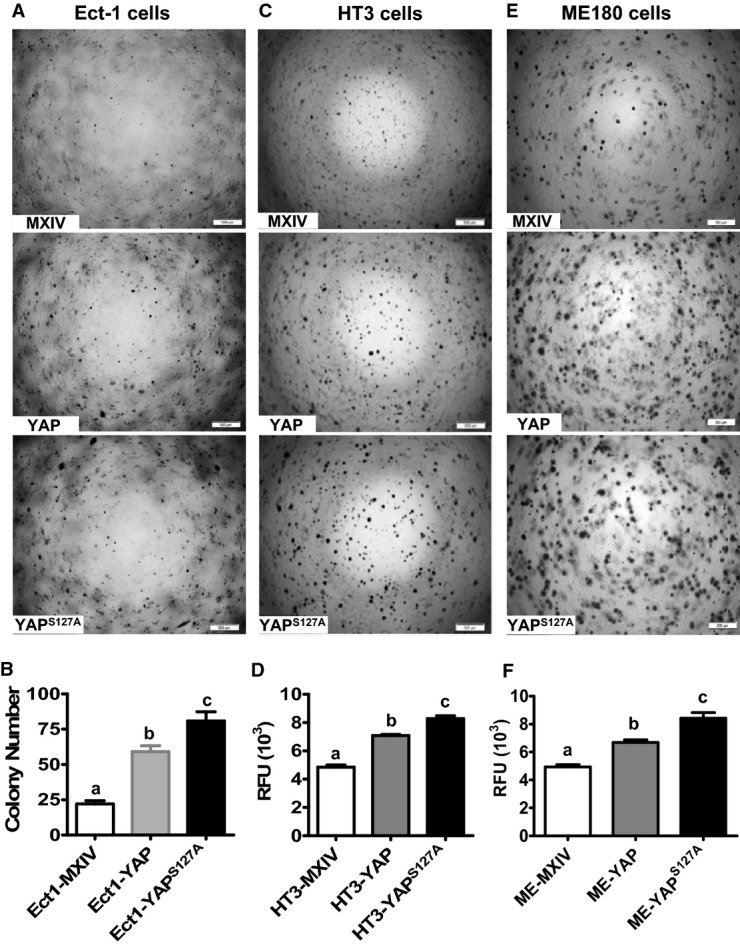
Effect of YAP on the anchorage-independent growth of normal and cancerous cervical cells A Representative images showing colonies formed by Ect1-MXIV, Ect1-YAP, and Ect1-YAP^S^^127A^ cells after growth in soft agar for 9 days. Scale bar: 500 μm.

As mentioned above, Ect1/E6E7 (Ect1) cell was immortalized with HPV16 E6/E7 and is considered to be an immortalized ectocervical epithelial cell line (Fichorova *et al*, 1997; Fichorova & Anderson, 1999). Therefore, the three cell lines derived from this cell line are proper cellular models to determine whether YAP can transform cervical epithelial cells using a soft agar colony formation assay. When grown on soft agar, Ect1-MXIV cells only formed a few very small, slow-growing colonies after 9 days. However, Ect1-YAP cells and Ect1-YAP^S127A^ cells formed many large colonies on soft agar (Fig3A and B). The formation of colonies by the Ect1-MXIV may be attributed to the fact that HPV16 E6 in these cells promoted YAP protein levels, which is evidenced in studies presented later in this report.

B Quantitative data showing colony formation in Ect1-MXIV, Ect1-YAP, and Ect1-YAP^S^^127A^ cells. Each bar represents mean ± SEM of five independent experiments. Bars with different letters are significantly different from each other (Ect1-MXIV vs. Ect1-YAP, *P *=* *0.0003; Ect1-MXIV vs. Ect1-YAP^S^^127A^, *P *=* *0.0002).

C, E Representative images showing the anchorage-independent growth of YAP-expressing HT3 (C) and ME180 (E) cervical cancer cell lines. Anchorage-independent cell growth was determined by the soft agar colony formation assay. Scale bar: 500 μm.

D, F Fluorescence-based quantitative analysis showing the differences of anchorage-independent growth in HT3-MXIV, HT3-YAP, and HT3-YAP^S^^127A^ (D) and ME180-MXIV, ME180-YAP, and ME180-YAP^S^^127A^ cells (F). The anchorage-independent cell growth was determined using a CytoSelect 96-Well Cell Transformation Assay kit, and data are presented as relative fluorescent units (RFU). Each bar represents mean ± SEM of four independent experiments. Bars with different letters are significantly different from each other (HT3-MXIV vs. HT3-YAP, *P *=* *0.0002; HT3-MXIV vs. HT3-YAP^S^^127A^, *P *<* *0.0001; ME-MXIV vs. ME-YAP, *P *=* *0.0011; ME-MXIV vs. ME-YAP^S^^127A^, *P *=* *0.0013). Data information: Data in (B), (D), and (F) were analyzed for significance using one-way ANOVA in GraphPad Prism 5 with Tukey’s *post hoc* tests. Source data are available online for this figure.

The soft agar assay for colony formation was also used to determine whether high levels of YAP enhanced the transformed phenotype of cervical cancer cell lines. As shown in Fig3C, HT3-YAP^S127A^ cells formed more colonies than HT3-YAP cells, while HT3-YAP cells formed more colonies than HT3-MXIV cells. Similarly, ME180-YAP^S127A^ cells formed more colonies than ME180-YAP cells, while ME180-YAP cells formed more colonies than ME180-MXIV cells (Fig3E). A fluorometric colony formation kit (CytoSelect™ 96-Well Cell Transformation Assay kit, Cell Biolabs, Inc.) was used to avoid the potential subjective results from manual colony counting. The results clearly showed that HT3-YAP^S127A^ (Fig3D) and ME180-YAP^S127A^ cells (Fig3F) had the highest anchorage-independent growth rate, while the HT3-MXIV and ME180-MXIV control cells had the lowest anchorage-independent growth rates (Fig3D and F).

### YAP enhances tumor growth *in vivo*

**Figure 4:**
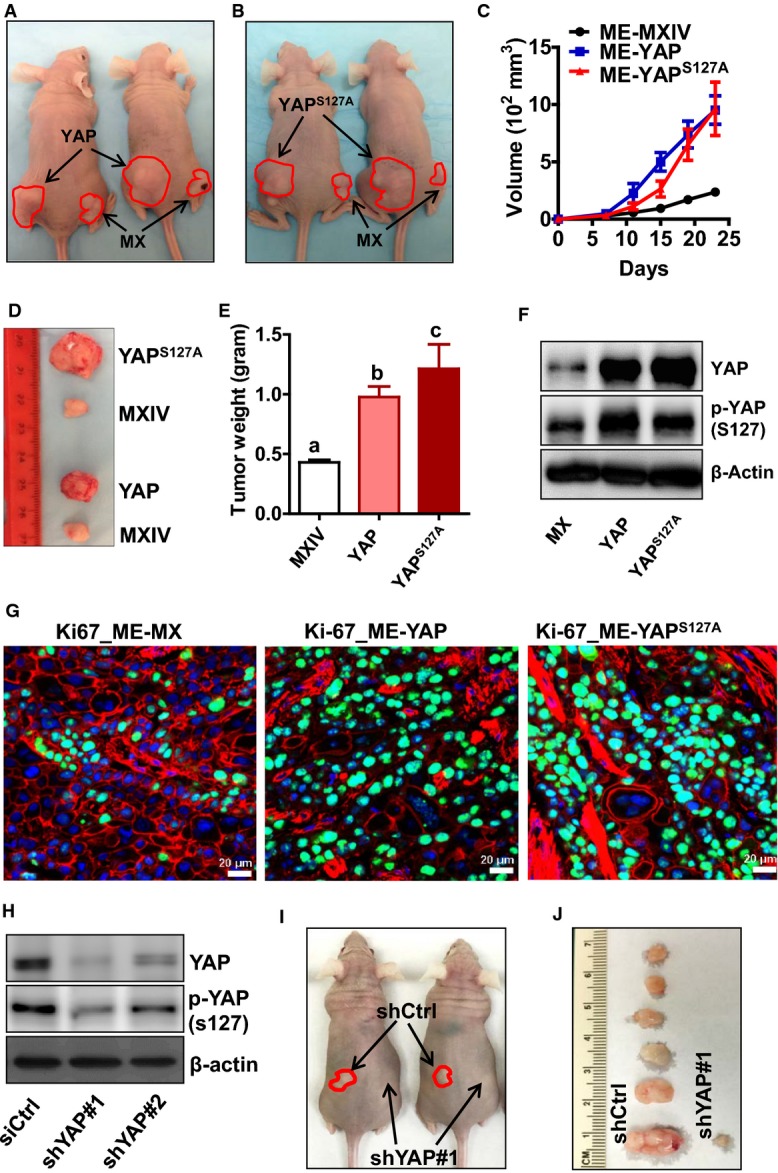
Effect of YAP on human cervical tumor growth *in vivo* A, B Representative images of tumor xenografts of ME180-MXIV (MX, right flank), ME180-YAP (A) (YAP, left flank), and ME180-YAP^S^^127A^ (B) (YAP^S^^127A^, left flank) cells implanted in athymic nude mice. Red lines indicate the edge of tumors.

A mouse xenograft tumor model was used to determine the effects of YAP on the progression of cervical cancer *in vivo*. The results showed that in comparison with ME180-MXIV cells, tumors derived from ME180-YAP and ME180-YAP^S127A^ cells were larger and detected earlier. On day 23 of tumor growth, the tumor volumes (mm^3^, mean ± SEM, *n* = 6) of the ME180-YAP group (952.5 ± 124.3) and the ME180-YAP^S127A^ group (963.1 ± 232.6) were significantly larger than that of the ME180-MXIV group (236.8 ± 23.4) (*P *<* *0.01) (Fig4A–D). The weights of the tumors in the YAP and YAP^S127A^ groups were also significantly higher than those of the control group (*n* = 6, *P *<* *0.001, Fig4E). Western blot analysis confirmed the overexpression of YAP protein in both ME180-YAP and ME180-YAP^S127A^ tumor xenografts (Fig4F). Fluorescent immunohistochemistry clearly indicated that tumor tissues derived from ME180-YAP and ME180-YAP^S127A^ tumor xenografts had higher expression of Ki67, a known cell proliferation marker, confirming our *in vitro* observations that YAP regulates the proliferation of cervical cancer cells *in vivo* (Fig4G, Appendix Fig S8).

C The growth curve of human tumor xenografts derived from control (ME-MX) and YAP-overexpressing ME180 cervical cancer cell lines (ME-YAP, ME-YAP^S^^127A^) implanted in the athymic nude mice. Each point represents mean ± SEM of six tumors (control *n *=* *10).

D Representative images showing the relative size of tumors from control and YAP-overexpressing ME180 cervical cancer cells.

E The average weight of tumor xenografts from the control and YAP-overexpressing ME180 cell lines. Data were analyzed for significance using one-way ANOVA in GraphPad Prism 5 with Tukey’s *post hoc* tests. Each bar represents the mean ± SEM (*n *=* *10 for control, *n *=* *6 for YAP and YAP^S^^127A^ groups). Bars with different letters are significantly different from each other (MXIV vs. YAP, *P *=* *0.0008; MXIV vs. YAP^S^^127A^, *P *<* *0.0001).

F Western blot analysis of YAP protein levels in the tumor xenografts derived from ME180-MXIV, ME180-YAP, and ME180-YAP^S^^127A^ cells.

G Expression of Ki67 in the tumor xenografts of ME180-MXIV, ME180-YAP, and ME180-YAP^S^^127A^ cells implanted in athymic nude mice. Ki67 was visualized using an Alexa-488 (green)-conjugated secondary antibody. Actin was visualized using an Alexa-594 (red)-conjugated secondary antibody. Nuclei were stained with DAPI. Scale bar: 20 μm. Note the significant increase in the Ki67-positive cells in the ME180-YAP and ME180-YAP^S^^127A^ tumor xenografts.

H Western blot analysis showing YAP protein levels in ME180 cells transfected with lentiviral empty vector (shCtrl) or lentivirus-based YAP shRNAs (shYAP#1 or shYAP#2).

I Representative images showing tumor xenografts derived from ME180-shCtrl cells (left flank) and ME180-shYAP#1 cells (right flank) (*n *=* *6).

J Representative images showing the relative size of tumors derived from ME180 cervical cancer cells transfected with control shRNA (left, shCtrl) or YAP shRNA#1 (right, shYAP#1) (*n *=* *6). Source data are available online for this figure.

To further confirm that YAP plays a role in regulating the proliferation of cervical cancer cells *in vivo*, we used lentivirus-based YAP shRNAs to knock down YAP protein in ME180 cells. Non-targeting shRNA was used as a control (shCtrl). Western blot analysis demonstrated that YAP shRNAs successfully reduced total and phosphorylation of YAP protein level in ME180 cells (Fig4H). Injection (SC) of ME180-CTRL cells into the athymic nude mice induced tumor formation in 100% (6/6) of the mice within 2 weeks. However, injection of the YAP-knockdown ME180-shYAP#1 cells into the nude mice induced tumor formation in only one mouse (1/6). Moreover, the tumor derived from ME180- shYAP#1 cells grew very slowly (Fig4I and J). These results indicated that YAP protein is essential for cervical tumor formation and tumor cell growth *in vivo*.

### TGF-α, which is up-regulated by YAP, promotes proliferation of cervical cancer cells

**Figure 5:**
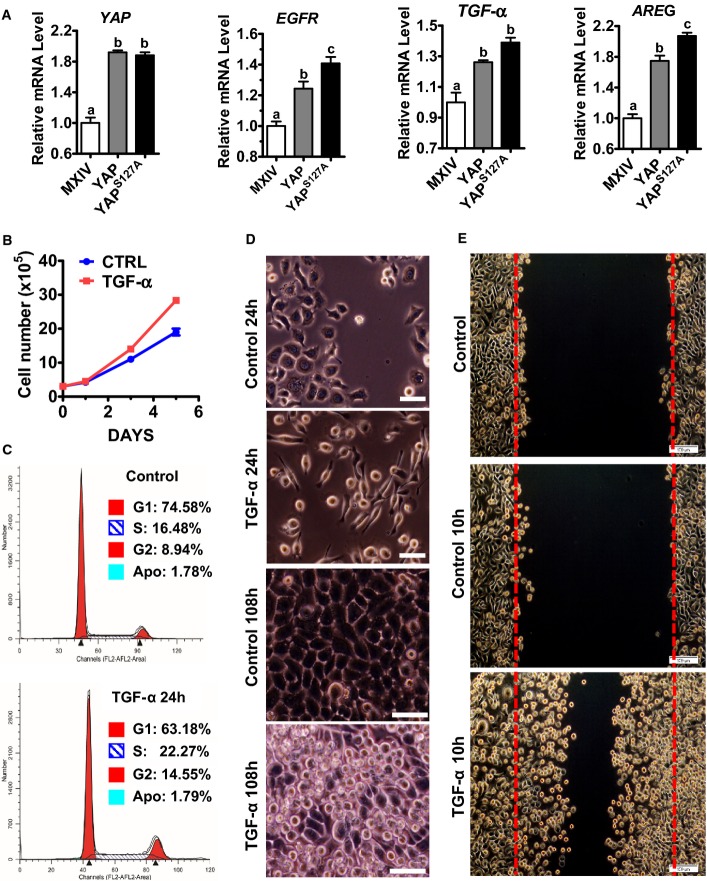
YAP stimulates expression of EGF-like ligands and EGFR in cervical cancer cell to promote cervical cancer cell proliferation and migration RT–PCR results showing that YAP stimulates the mRNA expressions of EGFR, AREG, and TGF-α in ME180 cervical cancer cell. Data were analyzed for significance using one-way ANOVA in GraphPad Prism 5 with Tukey’s *post hoc* tests. Each bar represents mean ± SEM (*n *=* *4). Bars with different letters are significantly different from each other (YAP mRNA: MXIV vs. YAP, *P *=* *0.0003; MXIV vs. YAP^S^^127A^, *P *=* *0.0001; EGFR mRNA: MXIV vs. YAP, *P *=* *0.0066; MXIV vs. YAP^S^^127A^, *P *=* *0.0003; TGF-α mRNA: MXIV vs. YAP, *P *=* *0.0235; MXIV vs. YAP^S^^127A^, *P *=* *0.0040; AREG mRNA: MXIV vs. YAP, *P *=* *0.0002; MXIV vs. YAP^S^^127A^, *P *<* *0.0001).

**Figure EV2:**
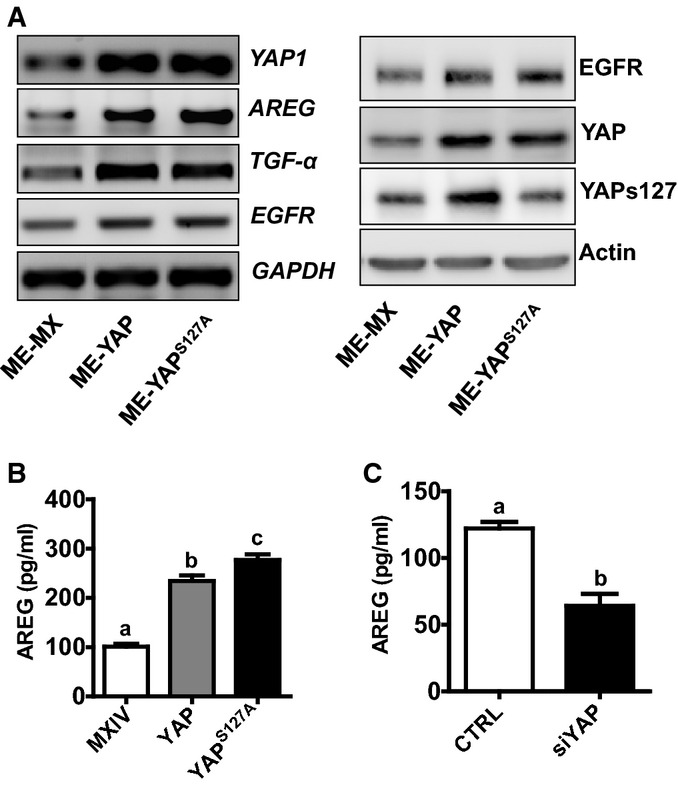
YAP regulated EGFR, AREG, and TGF-α expression in cervical cancer cell RT–PCR (left) and Western blot (right) were used to determine the expression of EGFR, AREG, and TGF-α in ME180-MXIV, ME180-YAP, and ME180-YAP^S^^127A^ cells. GAPDH and actin were used as controls.

Amphiregulin (AREG), a known YAP target gene, was up-regulated by YAP in cervical cancer ME180 cells (Figs5A and EV2A). Most importantly, overexpression or constitutive activation of YAP induced significant increase in the secretion of AREG in the culture medium (FigEV2B). Knockdown of YAP significantly reduced AREG concentrations in the culture medium (FigEV2C). Interestingly, overexpression of wild-type YAP or constitutively active YAP also dramatically increased expression of *TGF-α* and *EGFR* mRNA (Figs5A and EV2A). This observation is supported by the RNA sequencing data extracted from TCGA datasets, in which we found that YAP expression is significantly correlated with TGF-α and EGFR expression in cervical cancer (*P *=* *0.0009 and *P *=* *0.0122, respectively, FigEV3). Consistent with our observations in ovarian granulosa cell tumors (Wang *et al*, 2012a), TGF-α significantly enhanced proliferation of ME180 cells (Fig5B) and promoted cell cycle progression (Fig5C). Treatment of ME180 cells with TGF-α for 24 h resulted in elongation of ME180 cells (Fig5D). In growth medium, control ME180 cells formed a monolayer upon reaching confluence and had a marked reduction in growth rate. TGF-α-treated ME180 cells, however, continued to grow even after the cells reached confluence, leading to the formation of multilayer cell plaques (Fig5D). Wound-healing assays showed that TGF-α (10 h) treatment dramatically induced wound closure in ME180 cells, indicating that TGF-α also induced cervical cancer cell migration (Fig5E).

Proliferation of ME180 cells incubated in medium containing 1% FBS in the absence (control) or presence of 10 ng/ml TGF-α. Data were analyzed with unpaired *t*-test in GraphPad Prism 5 with Welch’s correction. Each point represents the mean ± SEM of four independent repeats. ***P <* *0.0001 versus control on day 5.

TGF-α treatment (10 ng/ml, 24 h) promotes ME180 cell cycle progression. G1, S, and G2 indicate cells in G1 phase, DNA synthesis phase, and the G2/M phase, respectively, of cell cycle. Apo: apoptotic cells.

Representative images showing the morphology of ME180 cells with or without TGF-α (10 ng/ml) treatment for 24 h (scale bar: 50 μm) or 108 h (scale bar: 25 μm). Please note the elongation of ME180 cells after TGF-α treatment for 24 h (TGF-α, 24 h) and the formation of multiple layers in ME180 cells after TGF-α treatment for 108 h (TGF-α, 108 h).

Effect of TGF-α on the migration of ME180 cells. TGF-α treatment (100 ng/ml, 10 h) drastically stimulated the migration of ME180 cells. Source data are available online for this figure.

The concentration of AREG in cell culture medium from ME180-MXIV, ME180-YAP, and ME180-YAP^S^^127A^ cells. The concentration of AREG in the medium was determined by AREG ELISA kit. Each bar represents mean ± SEM (*n *=* *5). Bars with different letters are significantly different from each other (ME-MX vs. ME-YAP, *P *<* *0.0001; ME-MX vs. ME-YAP^S^^127A^, *P *<* *0.0001).

The concentrations of AREG in ME180 cells with or without siYAP treatment. ME180-CTRL and ME180-siRNA cells were incubated in the FBS 1% medium for 48 h. Each bar represents mean ± SEM (*n *=* *5). Bars with different letters are significantly different from each other (*P *=* *0.0006). Data information: Quantitative data in (B) were analyzed for significance using one-way ANOVA in GraphPad Prism 5 with Tukey’s *post hoc* tests. Data in (C) were analyzed for significance with unpaired *t*-test in GraphPad Prism 5 with Welch’s correction.

**Figure EV3:**
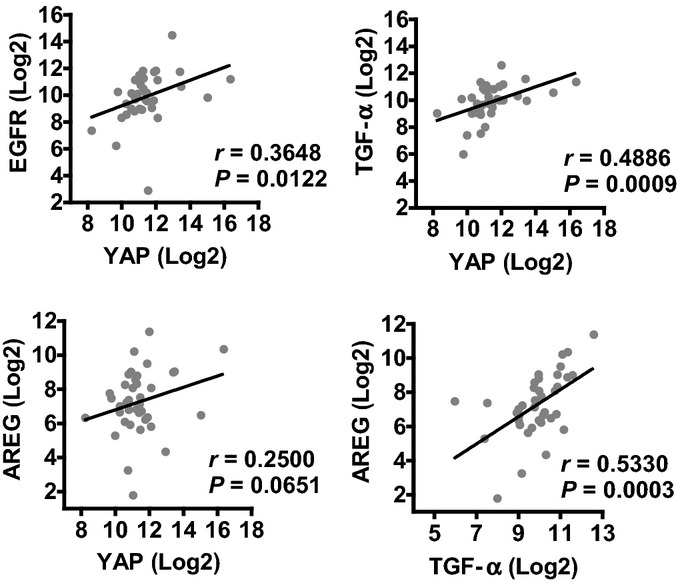
*YAP* mRNA expression is correlated with TGF-α, EGFR, and AREG in cervical cancer tissues mRNA expression data (*n *=* *39) were extracted from TCGA RNA sequencing datasets and were analyzed with linear regression analysis in GraphPad Prism 5 (GraphPad Software, Inc., La Jolla, CA). These data indicate that YAP mRNA expression is significantly correlated with TGF-α (*P *=* *0.001) and EGFR (*P *=* *0.0122) mRNA expression. Statistical analysis shows that YAP mRNA expression is not significantly correlated with AREG mRNA expression (*P *=* *0.065). This may be attributed to the limited cervical cancer sample number used for RNA-seq (*n *=* *39). TGF-α mRNA expression is significantly correlated with AREG mRNA expression in examined cervical cancer samples (*P *=* *0.0003).

### The Hippo signaling pathway interacts with TGF-α/EGFR signaling to regulate cervical cancer cell proliferation and migration

**Figure 6:**
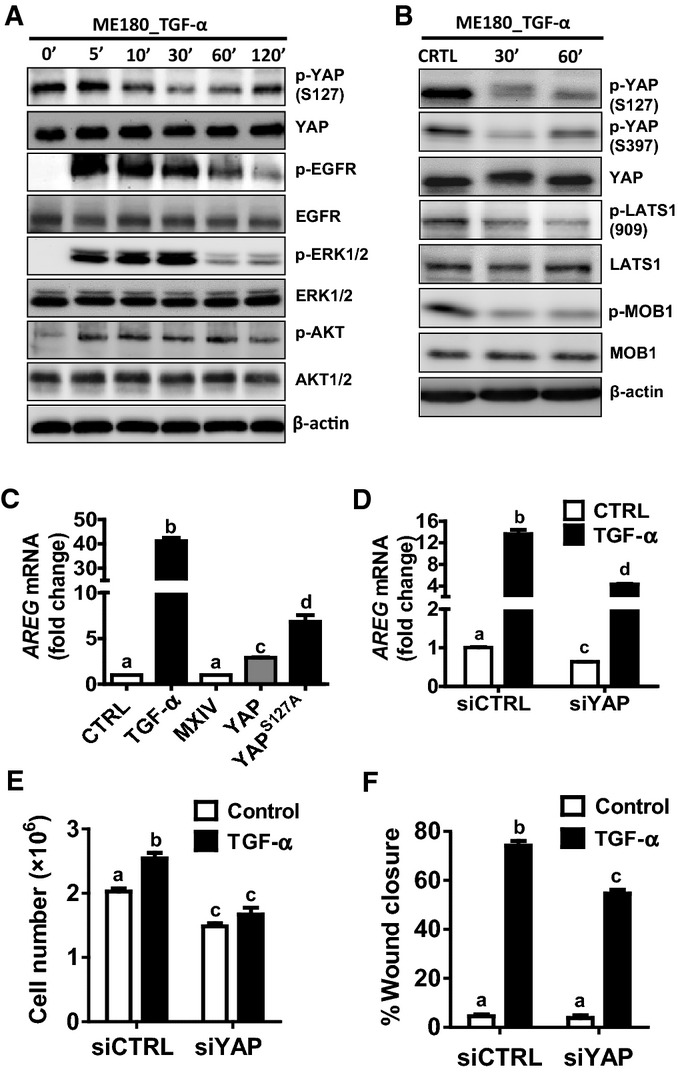
TGF-α regulates the Hippo signaling pathway in cervical cancer cells Western blot analysis showing effects of TGF-α on phosphorylation of EGFR, YAP, AKT, and ERK1/2. ME180 cells were starved for 6 h after reaching cell confluence, then cells were treated with TGF-α (100 ng/ml) for 0, 5, 10, 30, 60 or 120 min.

TGF-α treatment induced multilayer growth of ME180 cells, a phenotype that was observed in ME180 cells transfected with constitutively activated YAP (Fig5), suggesting potential involvement of the Hippo pathway in this process. Treatment of ME180 cells with TGF-α resulted in a rapid increase in the phosphorylation of the EGFR and activation of the PI3K and MAPK signaling pathways (Fig6A). TGF-α also rapidly suppressed phosphorylation of YAP at serine 127 and serine 397 in ME180 cells (Fig6A and B, Appendix Fig S9A). The ability of TGF-α to suppress YAP phosphorylation was also observed in HT3 and End1 cells (Appendix Fig S10). Moreover, LATS1 and MOB1 were dephosphorylated by TGF-α treatment (Fig6B, Appendix Fig S9B and C). Dephosphorylation of LATS1/2 and MOB1 results in the dissociation of LATS1/2-MOB1 complex, leading to suppression of the Hippo signaling pathway and activation of YAP (Pan, 2010; Yu & Guan, 2013). These observations indicate that the EGFR pathway interacts with the Hippo pathway to regulate the proliferation of cervical cancer cells. To further confirm that TGF-α treatment increases YAP transcriptional activity, we determined the mRNA level of amphiregulin (*AREG*), a known YAP target gene (Zhang *et al*, 2009; Hong *et al*, 2014). qRT-PCR analysis showed that *AREG* mRNA levels in ME180-YAP and ME180-YAP^S127A^ cells were increased by 2.9- and 6.8-fold, respectively, compared to ME180-MXIV control cells (Fig6C). Treatment of ME180 cells with TGF-α led to a 40-fold increase in *AREG* mRNA (Fig6C). Knockdown of YAP significantly suppressed TGF-α-stimulated expression of *AREG* mRNA (Fig6D). RNA sequencing data extracted from TCGA datasets also showed that TGF-α mRNA level was significantly correlated with *AREG* mRNA expression in cervical cancer (*P *=* *0.0003, Fig EV3). This evidence clearly suggests that in cervical cancer cells, the TGF-α/EGFR pathway interacts with the Hippo/YAP signaling pathway to form an autocrine/paracrine loop, which may play critical role in regulating cervical cancer progression.

Western blot analysis showing effects of TGF-α on the phosphorylation of the components of the Hippo signaling pathway. ME180 cells were starved for 6 h after reaching cell confluence, then cells were treated with TGF-α (100 ng/ml) for 0, 30, 60 min.

Real-time PCR determines the action of TGF-α and YAP levels on the mRNA expression of *AREG*. Each bar represents the mean ± SEM (*n *=* *5). Bars with different letters are significantly different from each other (Ctrl vs. TGF-α, *P *<* *0.0001; MXIV vs. YAP, *P *<* *0.0001; MXIV vs. YAP^S^^127A^, *P *<* *0.0001).

Real-time PCR analysis showing that knockdown of YAP with YAP siRNA (siYAP) significantly suppressed TGF-α-induced (FBS 10%, TGF-α: 10 ng/ml for 48 h) *AREG* expression in ME180 cells. siCTRL, a non-targeting siRNA, was used as a negative control. Each bar represents the mean ± SEM (*n *=* *5). Bars with different letters are significantly different from each other (siCTRL vs. siCTRL+TGF-α, *P *<* *0.0001; siYAP+CTRL vs. siYAP+TGF-α, *P *<* *0.0001; siCTRL+TGF-α vs. siYAP+TGF-α, *P *=* *0.0067).

Proliferation of ME180 cells (FBS 1%) treated with control (siCTRL) or YAP (siYAP) prior to treatment with control medium or TGF-α (10 ng/ml) for 108 h. Each bar represents the mean ± SEM (*n *=* *4). Bars with different letters are significantly different from each other (siCtrl vs. siCTRL+TGF-α, *P *=* *0.0058; siYAP+CTRL vs. siYAP+TGF-α, *P *=* *0.1840; siCTRL+TGF-α vs. siYAP+TGF-α, *P *=* *0.0013).

Quantitative data of the wound-healing assay showing the migration of ME180 cells that were treated with control (siCTRL) or YAP siRNA (siYAP) prior to treatment with or without TGF-α for 12 h. Each bar in bar graphs represents the mean ± SEM (*n *=* *4). Bars with different letters are significantly different from each other (siCtrl vs. siCTRL+TGF-α, *P *<* *0.0001; siYAP+CTRL vs. siYAP+TGF-α, *P *<* *0.0001; siCTRL+TGF-α vs. siYAP+TGF-α, *P *=* *0.0041). Data information: Data in (C–F) were analyzed for significance using one-way ANOVA in GraphPad Prism 5 with Tukey’s *post hoc* tests. Source data are available online for this figure.

Experiments were performed to determine whether the signaling pathways activated by TGF-α would affect the phosphorylation of YAP. Treatment of ME180 cells with EGFR inhibitor AG1478 completely prevented the TGF-α-induced dephosphorylation of YAP (Appendix Fig S11). Treatment with the PI3K inhibitor LY294002 or the MEK inhibitor U0126 partially but significantly blocked TGF-α-stimulated YAP protein dephosphorylation, suggesting that the EGFR/PI3K and EGFR/MEK/ERK signaling pathways are involved in mediating the actions of TGF-α on the Hippo pathway in cervical cancer (Appendix Fig S11).

To determine whether YAP plays a role in TGF-α-stimulated growth of cervical cancer cells, we knocked down the expression of YAP in ME180 cells using siRNA and then treated these cells with TGF-α. Results showed that TGF-α promoted ME180 cell proliferation in the control group, but it failed to do so in the YAP-knockdown ME180 cells (Fig6E). Moreover, knockdown of YAP in ME180 cells diminished TGF-α-stimulated ME180 cell migration, as indicated by the significant decrease in the wound closure in YAP-knockdown ME180 cells after TGF-α treatment (Fig6F, Appendix Fig S12). Taken together, these results indicate that the effects of TGF-α on cervical cancer cell proliferation and migration require, at least in part, via activation of YAP protein.

### AREG activates YAP protein and induces cervical cancer cell growth

**Figure 7:**
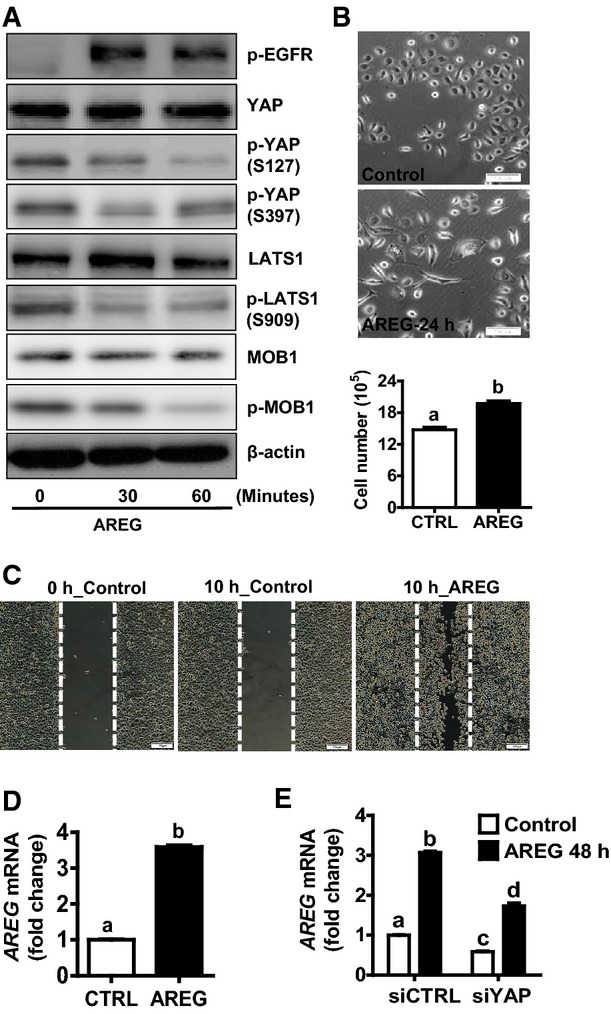
Function and expression of AREG in cervical cancer cells Western blot analysis showing that AREG treatment induced dephosphorylation of LATS1, MOB1, and YAP. ME180 cells were starved for 6 h after reaching cell confluence, then cells were treated with AREG (50 ng/ml) for 0, 30, 60 min.

We found that TGF-α treatment and YAP overexpression significantly increased *AREG* mRNA level (Fig6). Since AREG is a member of the family of the EGF-like ligands and we have shown that the EGFR pathway interacts with the Hippo pathway to regulate cervical cancer cell growth, we infer that AREG may also be involved in the regulation of cervical cancer cell proliferation. Treatment of ME180 cells with recombinant human AREG increased phosphorylation of EGFR at Tyr1173 and reduced phosphorylation of YAP (at Ser127 and Ser397), LATS1 (Ser 909), and MOB1 (Thr35) within 30 min (Fig7A, Appendix Fig S13). Treatment of ME180 cells with AREG induced elongated cell morphology (within 24 h) and significantly increased cell proliferation (72 h) (Fig7B). Moreover, AREG potently stimulated ME180 cell migration, as indicated by the significant increase in the wound closure in the wound-healing assay (Fig7C). Most interestingly, we found that the *AREG* mRNA expression was induced by AREG itself in cultured ME180 cervical cancer cells (Fig7D). Knockdown of YAP with YAP siRNA significantly suppressed AREG-stimulated *AREG* mRNA expression (*P *<* *0.0001) (Fig7E).

AREG (50 ng/ml) treatment induced appearance of elongated cells in ME180 cells (top and middle panels) and significantly stimulated ME180 cell proliferation (lower panel). Each bar represents mean ± SEM (*n *=* *5). Bars with different letters are significantly different from each other (*P = *0.003). Scale bar: 100 μm.

Wound-healing assay showing that AREG stimulates migration of ME180 cervical cancer cells within 10 h in serum-free medium. Scale bar: 200 μm.

Real-time PCR showing that treatment of ME180 cells with AREG for 24 h significantly increased *AREG* mRNA expression. Each bar represents mean ± SEM (*n *=* *9). Bars with different letters are significantly different from each other (*P < *0.0001).

Real-time PCR showing that knockdown of YAP in ME180 cells with YAP siRNA (siYAP) significantly suppressed AREG-induced AREG mRNA expression. siCTRL (non-target siRNA) was used as a siRNA control. Each bar represents mean ± SEM (*n *=* *4). Bars with different letters are significantly different from each other (siCTRL vs. siCTRL+AREG, *P *<* *0.0001; siYAP+CTRL vs. siYAP+AREG, *P *<* *0.0001; siCTRL+AREG vs. siYAP+AREG, *P *=* *0.0037). Data information: Quantitative data in (B) and (D) were analyzed for significance using unpaired *t*-test in GraphPad Prism 5 with Welch’s correction. Data in (E) were analyzed for significance using one-way ANOVA in GraphPad Prism 5 with Tukey’s *post hoc* tests. Source data are available online for this figure.

### The interaction between the Hippo/YAP and the EGFR signaling pathways regulates cervical cancer cell growth

**Figure EV4:**
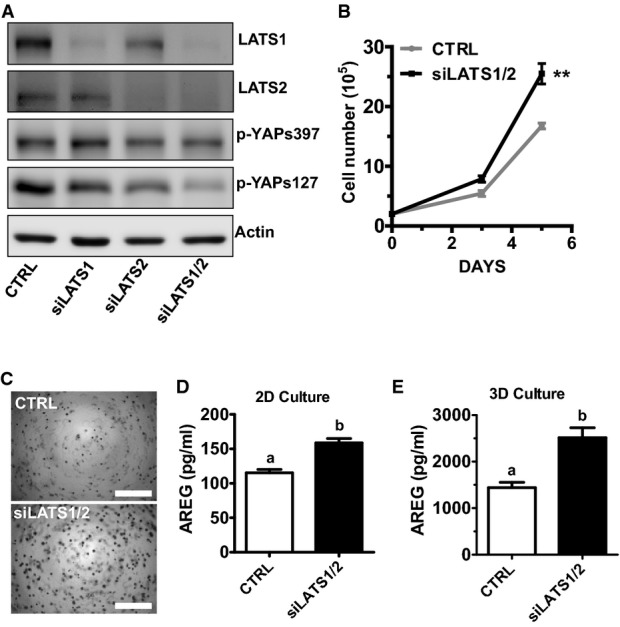
Effect of LATS1/2 on the growth of cancerous cervical cells A Western blot analysis showed that LATS1/2 siRNAs successfully knocked down LATS1/2 and activated YAP in ME180 cells.

**Figure 8:**
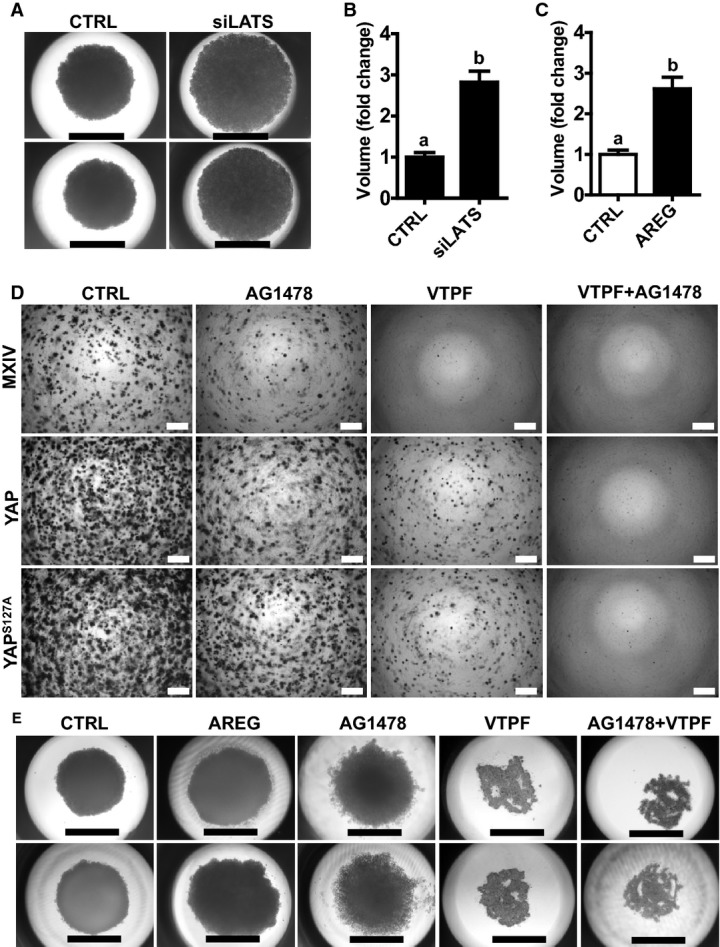
The Hippo/YAP and the ERBB pathways interact with each other to regulate cervical cancer cell growth Representative images showing the morphology of spheroids derived from ME180-siCtrl and ME180-siLATS1/2 cells growing in a 3D hanging-drop culture system for 10 days. Scale bar: 1.0 mm.

Treatment of confluent cervical cells with TGF-α and AREG resulted in a rapid and significant decrease in phosphorylation of LATS1, MOB1, and YAP (Figs6A and B and 7A, Appendix Figs S9, S10 and S13), suggesting that the Hippo pathway may involve in the YAP and EGFR signaling interaction. LATS1 and LATS2 are main components of the Hippo pathway and can directly phosphorylate YAP at Ser127. Knockdown of LATS1/2 in ME180 cells with LATS1/2 siRNAs activated YAP, which is indicated by a significant decrease in phospho-YAP (S127) (FigEV4A). Knockdown of LATS1/2 in ME180 cells also significantly increased cell proliferation and enhanced anchorage-independent cell growth (FigEV4B and C). The advantage of 3D culture, especially its high physiological relevance, has been reported (Friedrich *et al*, 2009). We found that knockdown of LATS1/2 significantly induced cell growth in the 3D culture system (Fig8A and B). Importantly, knockdown of LATS1/2 significantly increased the AREG secretion in both 2D and 3D culture (Fig EV4D and E). Consistent with 2D culture results, treatment of ME180 cells with AREG also significantly stimulated cell growth in the 3D culture system (Fig8C and E).

B Knockdown of LATS1/2 promoted proliferation of ME180 cells. Each point represents the mean ± SEM of five independent experimental results. **P <* *0.01 (*P *=* *0.0020).

C Representative images showing the anchorage-independent growth of ME180 with or without LATS1/2 knockdown (*n *=* *5). Scale bar: 1 mm.

D, E The concentrations of AREG in 2D (D) or 3D hanging-drop (E) culture medium from ME180 control and LATS1/2 knockdown cells. Each bar represents the mean ± SEM of five independent experimental results. Bars with different letters are significantly different from each other [CTRL vs. siLATS1/2 in (D), *P *=* *0.0017; CTRL vs. siLATS1/2 in (E), *P *=* *0.0045]. Data information: Data in (B), (D), and (E) were analyzed for significance with unpaired *t*-test in GraphPad Prism 5 with Welch’s correction.

Quantitative data showing changes in the volume of spheroids derived from ME180-siCTRL and ME180-siLATS1/2 cells growing in a 3D hanging-drop culture system. Each bar represents mean ± SEM (*n *=* *5). Bars with different letters are significantly different from each other (*P = *0.0004).

Quantitative data showing changes in the volume of spheroids derived from ME180 cells growing in a 3D hanging-drop culture system in the absence or presence of AREG (20 ng/ml, 8 days). Each bar represents mean ± SEM (*n *=* *5). Bars with different letters are significantly different from each other (*P = *0.0030).

Soft agar assay showing the effect of AG1478 and VTPF on colony formation in ME180-MXIV, ME180-YAP and ME180-YAP^S^^127A^ cells. Scale bar: 500 μm.

Representative images showing the effect of AREG, EGFR inhibitor (AG1478), and YAP antagonist verteporfin (VTPF) on the growth of ME180 cell in a 3D hanging-drop culture system. ME180 cells were incubated in the 3D hanging-drop culture system for 10 days in the absence or presence of AREG, AG1478 or verteporfin for 8 days. Scale bar: 1.0 mm. Data information: Quantitative data in (B) and (C) were analyzed for significance using unpaired *t*-test in GraphPad Prism 5 with Welch’s correction. Source data are available online for this figure.

**Figure EV5:**
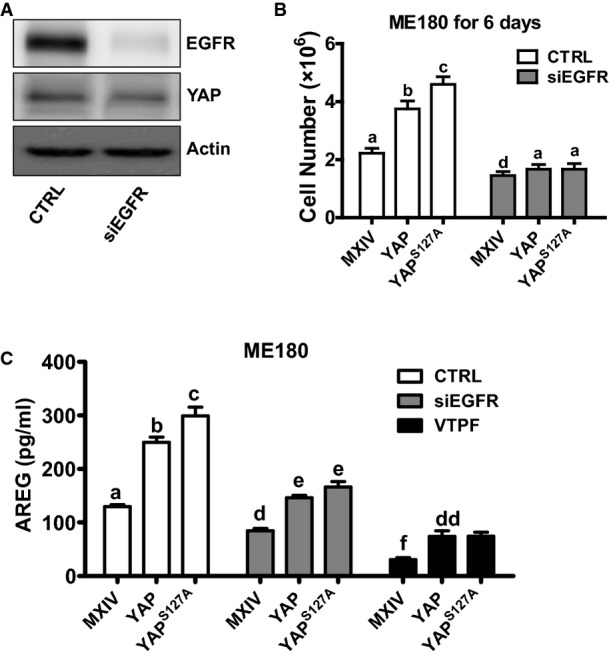
Knockdown of EGFR suppressed YAP-induced cell growth in cancerous cervical cells Western blot analysis showed that siRNAs of EGFR successfully knocked down EGFR in ME180 cells.

Both TGF-α and AREG specifically bind to EGFR to regulate cell proliferation. Knockdown of EGFR inhibits basal and YAP-induced growth of ME180 cell (FigEV5A and B). Most importantly, knockdown of EGFR significantly reduced basal and YAP-induced secretion of AREG (FigEV5C). In addition, the involvement of EGFR pathway in YAP regulating cervical cancer cell growth is further evidenced by the observation that treatment of ME180-YAP and ME180-YAP^S127A^ cells with AG1478 (EGFR inhibitor) dramatically blocked their ability to form colonies in the soft agar (Fig8D). We also found that verteporfin, an antagonist of YAP (Liu-Chittenden *et al*, 2012), not only suppressed the colony formation of ME180-MXIV, ME180-YAP, and ME180-YAP^S127A^ cells (Fig8D), but also reduced the production of AREG in these cells (Fig EV5C).

Knockdown of EGFR blocked YAP-induced proliferation of ME180 cells. Each bar represents the mean ± SEM of five independent experimental results. Bars with different letters are significantly different from each other (CTRL-MXIV vs. siEGFR-MXIV, *P *=* *0.0167; CTRL-YAP vs. siEGFR-YAP, *P *<* *0.0001; CTRL-YAP^S^^127A^ vs. siEGFR-YAP^S^^127A^, *P *<* *0.0001).

EGFR knockdown or verteporfin treatment dramatically decreased YAP-induced AREG secretion. Each bar represents the mean ± SEM of five independent experimental results. Bars with different letters are significantly different from each other (CTRL-MXIV vs. siEGFR-MXIV, *P *=* *0.0006; CTRL-MXIV vs. VTPF-MXIV, *P *<* *0.0001; CTRL-YAP vs. siEGFR-YAP, *P *=* *0.0007; CTRL-YAP vs. VTPF-YAP, *P *<* *0.0001; CTRL-YAP^S^^127A^ vs. siEGFR-YAP^S^^127A^, *P *<* *0.0001; CTRL-YAP^S^^127A^ vs. VTPF-YAP^S^^127A^, *P *<* *0.0001). Data information: Data in (B) and (C) were analyzed for significance using two-way ANOVA in GraphPad Prism 5.

On the soft agar, ME180-YAP and ME180-YAP^S127A^ cells formed much more, larger and fast-growing colonies in comparison with ME180-MXIV cells (Figs3C and 8D). We observed that colonies derived from ME180-YAP^S127A^ and ME180-YAP cells are somewhat resistant to AG1478 or verteporfin treatment. However, combined treatment with verteporfin and AG1478 completely blocked the growth of ME180-YAP^S127A^ cells on soft agar, suggesting that the combined targeting of the Hippo/YAP and EGFR pathways may be a more efficient way to inhibit cervical cancer cell growth (Fig8D).

We then used a more physiology-relevant 3D culture system to examine our finding. ME180 cervical cancer cells were loaded onto the 3D culture system and incubated for 3 days to form spheroids. The formed spheroids were treated with verteporfin or/and AG1478 for 6 days. We found that AG1478 treatment resulted in scattered distribution of ME180 cells and incompletely formed spheroids, indicating that blockade of EGFR could partially disrupt cervical cancer cell–cell communication (Fig8E). Verteporfin treatment completely blocked cancer cell growth and disrupted cervical cancer cell–cell communication, leading to the destruction of initially formed spheroids (Fig8E). Combined treatment with AG1478 and verteporfin also completely blocked the formation of spheroids in the 3D hanging-drop culture system (Fig8E).

### YAP is involved in HPV E6 regulation of cervical cancer cell growth

**Figure 9:**
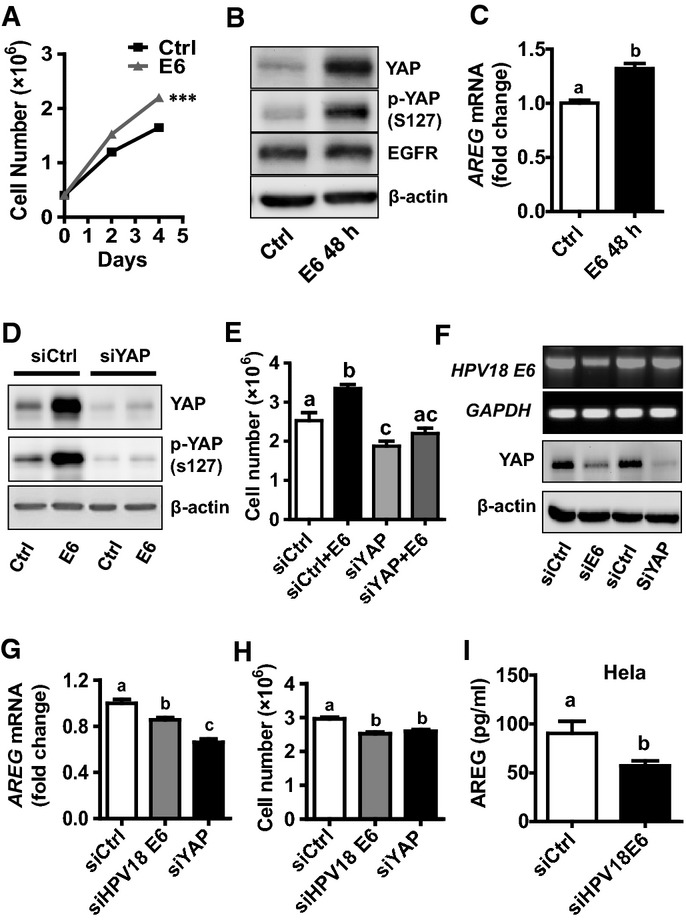
YAP is involved in HPV-E6 regulation of cervical cancer cell growth Effect of recombinant HPV16 E6 protein on the proliferation of HT3 cells. Each point represents mean ± SEM (*n *=* *5). ***P =* *0.0001 compared with control (Ctrl) on day 4. HT3 cells were cultured in serum-reduced medium in the presence or absence of recombinant HPV16 E6 (400 nM).

Epidemiological studies have shown that the high-risk HPV E6/E7 protein plays a critical role in the initiation and progression of cervical cancer. However, the exact molecular mechanism underlying the ability of high-risk HPV E6/E7 to regulate cervical cancer is largely unknown. Treatment of HT3 cells (cervical cancer cells without HPV infection) (Fogh *et al*, 1977; Yee *et al*, 1985) with HPV16 E6 protein significantly increased cancer cell growth (Fig9A). Surprisingly, treatment of HT3 cells with HPV16 E6 increased protein levels of total YAP and phosphorylated YAP (Ser127), but had no effect on the protein level of β-actin (Fig9B). Real-time PCR results showed that treatment of HT3 cells with HPV16 E6 for 48 h also significantly increased mRNA level of *AREG* (Fig9C). This finding is consistent with the observed increase in YAP protein levels since *AREG* is downstream gene of the Hippo/YAP pathway. Knockdown of YAP with YAP siRNA eliminated HPV16 E6-stimulated HT3 cell proliferation (Fig9D and E), further suggesting that YAP is an important mediator of HPV16 E6 action in cervical cancer cells.

Recombinant HPV16 E6 protein increased YAP protein level, but had no effect on β-actin protein level in HT3 cells. Cell culture and treatment procedure are the same as described in (A).

*AREG* mRNA levels in HT3 cells incubated for 48 h with or without recombinant HPV E6. Each bar represents mean ± SEM (*n *=* *5). Bars with different letters are significantly different from each other (*P* = 0.0042).

Western blotting analysis showing the effect of HPV16 E6 (400 nM, 48 h) on YAP protein levels in HT3 cells transfected with non-targeting control siRNA (siCtrl) or YAP siRNA (siYAP).

Effect of YAP on HPV16 E6 stimulation of HT3 cell proliferation. siCtrl: non-targeting control siRNA; siYAP: YAP siRNA; E6: 400 nM recombinant HPV16 E6, 48 h. Each bar represents mean ± SEM (*n *=* *4). Bars with the same letters are not significantly different from each other (siCtrl vs. siCtrl+E6, *P *=* *0.0232; siCtrl+E6 vs. siYAP+E6, *P *=* *0.0011).

Knockdown of endogenous E6 in HeLa cells with HPV18 E6 siRNA (siE6) reduced YAP protein in HeLa cells, while knockdown of YAP with YAP siRNA (siYAP) in these cells had no effect on the mRNA level of HPV18 E6. siCtrl: non-targeting control siRNA.

Knockdown of endogenous HPV18 E6 in HeLa cells with HPV18 E6-specific siRNA (siE6) significantly suppressed mRNA expression of *AREG*. Each bar represents mean ± SEM (*n *=* *4). Bars with different letters are significantly different from each other (siCtrl vs. siHPV18E6, *P *=* *0.0181; siCtrl vs. siYAP, *P *=* *0.0005).

Knockdown of endogenous HPV18 E6 in HeLa cells with HPV18 E6-specific siRNA (siE6) significantly suppressed cell growth (*n *=* *9, siCtrl vs. siHPV18E6, *P *=* *0.0002; siCtrl vs. siYAP, *P *=* *0.0004).

Concentrations of AREG in the culture medium of HeLa cells transfected with non-targeting control siRNA (siCTRL) or HPV18 E6 siRNA (siHPV18E6). Each bar represents mean ± SEM (*n *=* *6). Bars with different letters are significantly different from each other (*P *=* *0.0461). Data information: Quantitative data in (A), (C), and (I) were analyzed for significance using unpaired *t*-test in GraphPad Prism 5 with Welch’s correction. Data in (E), (G), and (H) were analyzed for significance using one-way ANOVA in GraphPad Prism 5 with Tukey’s *post hoc* tests.

Since HeLa cells express endogenous HPV18 E6 protein, we used this cell line to determine whether YAP also mediates the action of endogenous HPV E6 protein. Knockdown of HPV18 E6 with specific siRNA not only decreased *E6* mRNA level, but also reduced YAP protein levels (Fig9F), suppressed *AREG* mRNA expression (Fig9G), and inhibited HeLa cell proliferation (Fig9H). Moreover, knockdown of HPV18 E6 decreased AREG secretion in HeLa cells (Fig9I). Knockdown of YAP reduced YAP protein (Fig9F), suppressed *AREG* mRNA expression (Fig9G), and inhibited HeLa cell proliferation (Fig9H). Knockdown of YAP had no effect on the mRNA level of HPV18 *E6* (Fig9F). These results suggested that both recombinant and endogenous HPV E6 proteins were able to increase YAP protein levels.

### HPV E6 prevents YAP protein from degradation

**Figure 10:**
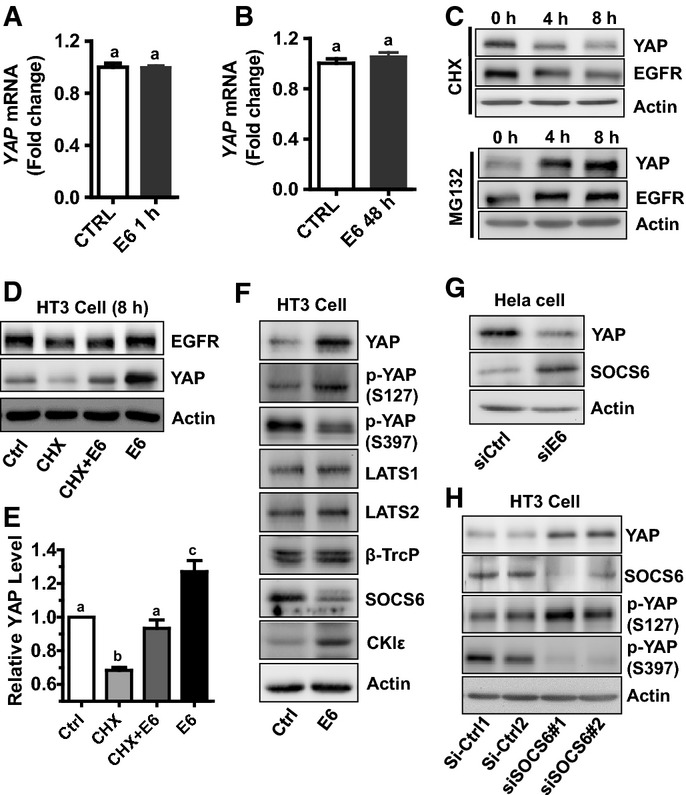
HPV E6 stabilizes YAP protein Effect of *YAP* mRNA levels in HT3 cells treated with or without E6 (400 nM) for 60 min in McCoy’s 5A with 1% serum. Data were normalized with 18S mRNA. Bars with same letters are not significantly different from each other (*P *>* *0.05). *n *=* *4, *P *=* *0.9407.

The HT3 cancer cell line, which is HPV negative (Fogh *et al*, 1977; Yee *et al*, 1985), was used to determine the mechanism underlying HPV16 E6 regulation of YAP protein level. Treatment of HT3 cells with HPV16 E6 increased YAP protein (Fig9B and D), but HPV16 E6 did not affect *YAP* mRNA expression (Fig10A and B). This suggests that HPV16 E6 may regulate YAP protein turnover. Treatment of HT3 cells with MG132, a potent proteasome inhibitor, for 4 h or 8 h drastically increased YAP and EGFR protein levels (Fig10C). In contrast, treatment of HT3 cells with cycloheximide (CHX), an inhibitor of eukaryotic gene translation, for 4 h or 8 h significantly reduced EGFR and YAP protein levels (Fig10C and D). This evidence suggests that YAP and EGFR proteins are continuously synthesized and degraded in a proteasome-dependent mechanism in cervical cancer cells. The addition of HPV16 E6 prevented the degradation of YAP protein in CHX-treated cells, but had little or no effect on the degradation of EGFR (Fig10D and E). These observations clearly indicate that HPV16 E6 stabilizes the YAP protein in cervical cancer cells.

*YAP* mRNA levels in HT3 cells treated with or without E6 (400 nM) for 48 h in McCoy’s 5A with 1% serum. mRNA levels were measured with real-time PCR. Data were normalized with 18S mRNA. Each bar represents mean ± SEM (*n *=* *5). Bars with same letters are not significantly different from each other (*P *>* *0.05) (*P *=* *0.3233).

Western blot results showing YAP and EGFR protein levels after treatment with MG132 or cycloheximide (CHX) for 4 h or 8 h. β-actin was used as a protein loading control. Confluent HT3 cells were starved for 4 h before treatment with MG132 (10 μM) or CHX (20 μg/ml) for 0, 4 or 8 h.

HPV16 E6 protein prevented YAP protein from degradation. Confluent HT3 cells were starved for 4 h before treatment with or without CHX (20 μg/ml), HPV16 E6, or CHX combined with HPV16 E6 for 8 h. Treatment of starved HT3 cells with HPV16 E6 (400 nM) for 8 h suppressed degradation of YAP, but not EGFR protein.

Quantitative data showing relative YAP protein levels in (D). Protein levels were normalized with β-actin and presented as ratios relative to that of control. Each bar represents mean ± SEM (*n *=* *4). Bars with different letters are significantly different from each other (Ctrl vs. CHX, *P *=* *0.0003; Ctrl vs. CHX+E6, *P *=* *0.2665; Ctrl vs. E6, *P *=* *0.0258).

Western blot analysis showing that expression of HPV16 E6 in HT3 cells increased the protein level of total YAP and casein kinase Iε, but decreased the protein level of SOCS6. Importantly, HPV16 E6 increased YAP phosphorylation at serine 127, but suppressed its phosphorylation at serine 397. HPV16 E6 had no effect on the level of β-TrcP, LATS1/2 and MOB1 in HT3 cervical cancer cells.

Western blot results showing that knockdown of endogenous E6 in HeLa cells with HPV18 E6 siRNA (siE6) reduced YAP protein levels, but increased SOCS6 protein levels.

Western blot analysis showing that knockdown of SOCS6 in HT3 cells with SOCS6 siRNA (siSOCS#1 and siSOCS6#2) increased total YAP protein, enhanced phosphorylation of YAP protein at serine 127, but suppressed phosphorylation of YAP at serine 397. Data information: Quantitative data in (A) and (B) were analyzed for significance using unpaired *t*-test in GraphPad Prism 5 with Welch’s correction. Data in (E) were analyzed for significance using one-way ANOVA in GraphPad Prism 5 with Tukey’s *post hoc* tests.

To explore the mechanisms underlying HPV E6 stabilizing YAP protein, we transfected HT3 cells with lentivirus empty control vector (Ctrl) or lentivirus-based HPV16 E6-expressing vector and established HT3-CTRL and HT3-E6 cell lines. Western blotting results indicated that expression of E6 in HT3 cells also increased the proteins level of total YAP and Ser127-phosphorylated YAP (Fig0F), but dramatically decreased Ser397-phosphorylated YAP (Fig0F). It has been reported that phosphorylation of YAP (YAP protein isoform 1) at Ser397 (corresponds to Ser381 in YAP protein isoform 2) primes YAP for subsequent phosphorylation by CK1δ/ε, leading to β-TrCP (SCF) ubiquitin ligase-dependent proteolytic degradation of YAP protein (Zhao *et al*, 2010). Surprisingly, we found that expression of E6 in HT3 cells had little or no effect on the β-TrCP and LATS1/2, but it increased the protein level of CK1δ/ε (Fig0F). The HPV E6-induced decrease in p-YAP (S397) and increase in CK1δ/ε suggested that the CK1δ/ε may not be actively involved in HPV E6 stabilization of YAP protein.

Hong *et al* (2014) recently suggested SOCS6 can directly bind to YAP and induce YAP degradation. Interestingly, we found that expression of HPV16 E6 reduced the protein level of SOCS6 in HT3 cells. Knockdown of HPV18 E6 increased the protein level of SOCS6 in HeLa cells (Fig10F and G). These results indicate that SOCS6 may play a role in HPV E6-mediated stabilization of YAP. To confirm our finding, we knocked down SOCS6 in HT3 cells using SOCS6 siRNAs. Western blot analysis demonstrated that knockdown of SOCS6 increased total YAP and phosphorylation of YAP at Ser127 levels, but decreased phosphorylation of YAP at Ser397 (Fig0H). Clearly, SOCS6 is actively involved in HPV E6 stabilizing YAP protein in the cervical cancer cells.

### YAP expression in HPV16 E6/E7-induced mouse cervical tumors and HPV16-containing human foreskin keratinocytes “raft” cultures

**Figure 11:**
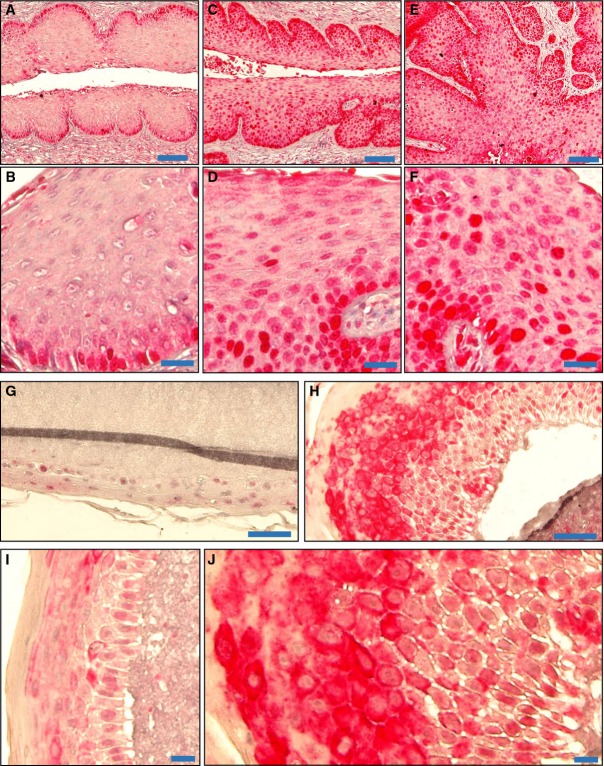
YAP expression in normal, HPV16 E6, and HPV16 E6/E7-induced cancerous cervical tissues in a transgenic mouse model, and HPV16-containing human foreskin keratinocyte raft cultures A–F Representative images showing expression of YAP (in red) in wild-type mouse cervical tissues (*n *=* *5) (A), HPV16 E6-induced (C) and HPV16 E6/E7-induced (E) mouse cervical tumor tissue (*n *=* *4 each). Scale bars for (A), (C), and (E): 100 μm. High-resolution images showing the expression and cellular location of YAP in normal cervical tissues (B), HPV16 E6-induced (D) and HPV16 E6/E7-induced (F) mouse cervical tumor tissue. Scale bars for (B), (D), and (F): 25 μm.

Transgenic mouse models were used to determine whether HPV E6 also effects YAP protein level *in vivo*. Previous studies showed that HPV16-E6 or HPV16-E6/E7 double-transgenic mice treated for 6 months with estrogen can develop cervical cancers (Brake & Lambert, 2005; Shai *et al*, 2007). Consistent with our results, we found that in comparison with the control mouse cervical tissues (Fig11A and B), YAP is highly expressed in the E6 (Fig11C and D) and E6/E7-induced cervical tumor tissues (Fig11E and F) and YAP is mainly localized to nucleus of tumor cells (Fig11D and F).

G, H Representative images showing expression of YAP (in red) in normal (G) and HPV16-containing human foreskin keratinocyte raft cultures (*n *=* *5 each) (H). Scale bars: 100 μm.

I, J High-resolution images showing the expression and cellular location of YAP in normal (I) and HPV16-containing human foreskin keratinocytes raft cultures (J). Scale bars: 20 μm.

The HPV16-containing organotypic human foreskin keratinocyte (HFK) “raft” culture system provides an unique model to investigate the life cycle of HPV16 (Lambert *et al*, 2005; Wang *et al*, 2009). We used the HFK raft culture system to confirm whether the wild-type HPV16 also affects YAP protein levels in HFKs, which share many similar features with the basal epithelial cells in the cervical epithelia. IHC staining results showed that keratinocytes in HPV16 plasmid-containing raft cultures were hyperproliferative compared with cells in HPV-free HFK raft cultures (control) (Fig11G and H). More importantly, we found that YAP signal intensity in keratinocytes in HPV16-containing raft cultures was much higher in comparison with that of control (Fig11G–J). Moreover, our results showed that the majority of the YAP immunosignal was localized to the mid- to upper spinous cells, with relatively lower signals observed in basal and parabasal cells (Fig11H). The expression level and distribution of YAP in the HPV16-containing organotypic HFK raft culture system perfectly match the YAP expression pattern and HPV DNA distribution that are consistently observed in naturally occurring HPV lesions (Stoler & Broker, 1986; Xiao *et al*, 2014).

## Discussion

Epidemiological and molecular evidence indicates that high-risk HPV, especially HPV type 16 and type 18, plays a causative role in cervical cancer (Jemal *et al*, 2011). However, the molecular mechanism(s) underlying HPV initiation of cervical cancer remains unclear. Moreover, in HPV-infected patients, the majority of HPV-associated lesions regress spontaneously (Melnikow *et al*, 1998), indicating that additional genomic alterations are also necessary for transformation of cervical epithelial cells and progression of cervical cancer. The present study provides compelling evidence showing that the HPV E6 protein, the Hippo pathway, and the EGFR signaling pathway interact with each other to regulate cervical cancer progression.

Yes-associated protein is the major downstream effector of the Hippo pathway. Activation of the Hippo pathway results in phosphorylation and sequestration of YAP into the cytoplasm, leading to inactivation of YAP-regulated gene transcription (Fernandez *et al*, 2009; Pan, 2010). Elevated YAP expression and nuclear localization have been observed in multiple types of human cancers, including liver cancer, colon cancer, epithelial ovarian cancer, lung cancer, and prostate cancer (Overholtzer *et al*, 2006; Zender *et al*, 2006; Dong *et al*, 2007; Yuan *et al*, 2008; Zhang *et al*, 2009; Hall *et al*, 2010; Jemal *et al*, 2011; Hergovich, 2012; He *et al*, 2015). In liver cancer, YAP has been reported to be an independent prognostic marker for overall survival and disease-free survival (Xu *et al*, 2009). In epithelial ovarian cancer, research has shown that a high level of nuclear YAP is strongly associated with poor patient survival (Hall *et al*, 2010). Up to date, only one IHC study showed that YAP could function as a predictive marker for cervical cancer (Xiao *et al*, 2014). The role of YAP in cervical cancer is unclear. In the present study, we show that YAP is overexpressed in the cervical cancer tissues. Moderate/strong expression of YAP protein was observed in 91% of cervical cancer tissues, while moderate/strong expression of YAP protein was not observed in all 10 normal cervical tissues (Table1). Moreover, YAP expression was significantly correlated with the FIGO stage, the extent of tumor, and the degree of regional lymph node involvement. To verify the clinical relevance of YAP up-regulation in the cervical cancer, we performed a cross-cancer *YAP* gene alteration analysis by using the multidimensional cancer genomic datasets and online analysis tools. We surprisingly found that among all cancer types, cervical cancer has the highest frequency of *YAP* gene alterations. The subsequent network analysis indicated that many cell proliferation-associated genes that interacted with *YAP* were up-regulated in various degrees in examined cervical cancer cases. Our data provide evidence that YAP could be used as a potential prognostic biomarker for cervical cancer. Since we do not have patient survival data, we cannot correlate YAP expression data with cervical patient survival in the present study. However, the high level of expression and nuclear location of YAP protein in cervical cancer tissues, as well as the very high frequency of *YAP* gene amplification in the patient samples, strongly argue that YAP plays an important role in regulating the progression of cervical cancer.

The concept that YAP plays a role in regulating the progression of cervical cancer is further supported by the following evidence: (i) Knockdown of YAP significantly reduced the growth rate of ME180, HT3, and HeLa human cervical cell lines *in vitro* and suppressed cervical cancer tumorigenesis *in vivo*; (ii) ectopic expression of wild-type YAP or constitutively active YAP in cervical cancer cells significantly stimulated cancer cell growth *in vitro*; (iii) overexpression or constitutive activation of YAP in cervical cancer cells overcame the contact inhibition-induced cell growth inhibition; (iv) overexpression or constitutive activation of YAP in cervical cancer cells promoted cell cycle progression; and (v) finally, overexpression or constitutive activation of YAP in cervical cancer cells dramatically stimulated tumor growth *in vivo*. As we have shown in this study, YAP is overexpressed and localized to the nucleus of the cancer cells in the cervical cancer tissues. According to our *in vitro* and *in vivo* data, high levels of biologically active YAP protein in the nucleus of cervical cancer cells are expected to stimulate cancer cell proliferation and promote cervical cancer progression.

Notably, under low-density cell culture conditions, knockdown of YAP had no significant effect on the growth of cervical cancer cells incubated in complete medium (with 10% FBS). After the cultured cells achieved higher density, cell growth rate in the YAP-knockdown group decreased, while cells in the control group continued to proliferate. This result is consistent with the observation that when incubated in complete medium, ME180-YAP^S127A^, ME180-YAP, and ME180-MXIV cells had similar growth rates before reaching confluence. However, after the cells reached confluence, the ME180-YAP^S127A^ and ME180-YAP cells continued to grow, while the proliferation of ME180-MXIV cells almost stopped. These findings indicate that YAP may play a critical role in overcoming cell contact-induced inhibition of cell growth. Interestingly, under serum-reduced culture conditions (with 1% FBS), ME180-YAP^S127A^ and ME180-YAP cells have a significantly higher growth rate in comparison with ME180-MXIV cells (*P *<* *0.001), regardless of the cell density. The compensatory activity of YAP on cell growth with serum deprivation suggests that YAP may control the production of certain hormones or growth factors that are essential for the growth of cervical cancer cells.

**Figure 12:**
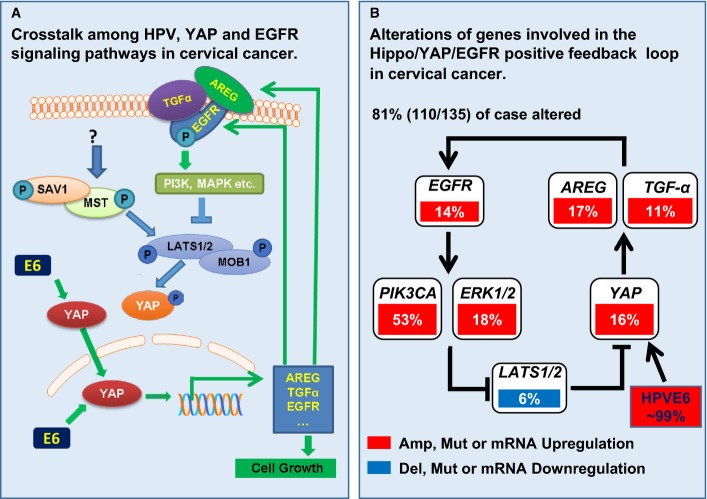
Schematic cartoons showing the proposed mechanism underlying the Hippo signaling pathway regulation of cervical cancer progression Schematic cartoons showing the proposed positive feedback loop in cervical cancer cells. Data in this study support the existence of an autocrine loop involving EGF-like ligands/EGFR pathway/Hippo pathway/EGF-like ligands in regulating the proliferation and motility of cervical cancer cells, which plays a critical role in cancer progression. Under normal conditions, low levels of EGF-like ligands such as TGF-α and AREG are not sufficient to activate EGFR. The inactive EGFR ensures the activation of Hippo signaling pathway, which in turn results in ubiquitination-dependent degradation of YAP protein. In cervical cancer tissues, especially the advanced-stage cancer tissues, the elevated nuclear YAP protein stimulates the expressions of TGF-α and AREG, which in turn activates the EGFR, leading to suppression of LATS1 and MOB1 phosphorylation. Dephosphorylation of LATS1 and MOB1 results in the dissociation of LATS1/2-MOB1 complex, leading to inactivation of the Hippo pathway and subsequent activation of the growth-promoting co-activator YAP. Activated YAP induces expression of growth factors such as TGF-α and AREG to drive cervical cancer growth. YAP-induced growth factors such as TGF-α and AREG complete the autocrine loop by activating EGFR and YAP to drive the proliferation of cancer cell and the production of growth-promoting factors in cervical cancer cells. HPV E6 protein maintains YAP protein level in the HPV-infected normal and cancerous cervical cells by preventing YAP from proteasome-dependent protein degradation.

The present study indicates that TGF-α and AREG are the candidate growth factors. The Hippo/YAP signaling pathway interacts with the ERBB signaling pathway to regulate cervical cancer cell proliferation and migration. This concept is supported by the following evidence: (i) Ectopic expression of wild-type YAP or constitutive active YAP in cervical cancer cells not only significantly stimulated *TGF*-α, *AREG*, and *EGFR* mRNA expression, but also induced AREG secretion; (ii) knockdown of LATS1/2, the major suppressor of YAP, stimulated secretion of AREG; (iii) TGF-α and AREG, via activation of EGFR, stimulated proliferation, promoted cell cycle progression, and enhanced migration of cervical cancer cells; (iv) TGF-α suppressed the Hippo signaling pathway, which was demonstrated by the significant reductions in the phosphorylation of LATS1, MOB1, and YAP after TGF-α treatment; (v) treatment of cervical cancer cell with TGF-α induced 40-fold increases in the transcription of AREG, a known downstream target of the Hippo/YAP signaling pathway (Zhang *et al*, 2009; Yu & Guan, 2013); and (vi) knockdown of YAP eliminated TGF-α-induced proliferation and migration of cervical cancer cells. Knockdown of YAP also suppressed TGF-α-induced AREG transcription in cervical cancer cells. We also found that AREG significantly stimulated cervical cancer cell proliferation and promoted cancer cell migration. Intriguingly, AREG was able to suppress phosphorylation of LATS1, MOB1, and YAP in the cervical cancer cells, suggesting that AREG is not only a downstream target of the Hippo pathway, but also an important upstream regulator of the Hippo/YAP signaling pathway. Remarkably, we found that treatment of cervical cancer cells with AREG resulted in a fourfold increase in the expression of *AREG* mRNA (*P *>* *0.001). Furthermore, blocking EGFR activity with AG1478, or knockdown of EGFR using siEGFR, eliminated YAP-induced cell proliferation and AREG secretion of cervical cancer cells. These observations, together with previous results, provide convincing evidence for the existence of an AREG/EGFR/Hippo signaling pathway/YAP/AREG autocrine loop in cervical cancer cells, which may play a critical role in the progression of cervical cancer (Fig12). Of relevance to our findings are previous reports showing that EGFR is overexpressed in cervical cancer and is associated with poor prognosis and decreased survival of cervical cancer patients (Schrevel *et al*, 2011; Soonthornthum *et al*, 2011). The existence of this feedback loop in human cervical cancer and its clinical relevance were further evidenced by the results derived from multidimensional cancer genomic data analysis. These analyses indicate that ∼81% of human cervical cancer cases have alterations in genes involved in this positive feedback loop (Fig12B). The synergetic suppressive effect of AG1478 and verteporfin on the growth of ME180-YAP and ME180-YAP^S127A^ cells and secretion of AREG clearly indicates that combined targeting of Hippo/YAP and EGFR pathways may represent a novel therapeutic strategy for cervical cancer (Figs8 and EV5).

Schematic cartoons showing the clinical relevance of the proposed positive feedback loop in cervical cancer. Multidimensional cancer genomics data analysis indicates that very high frequency of alterations has been observed in genes involved in the Hippo/YAP/EGFR positive feedback loop in cervical cancer (we acknowledge The Cancer Genome Atlas (TCGA) Data Portal for the datasets and the cBioPortal for Cancer Genomics for the online analysis tools).

Since Hippo-independent YAP activation has also been reported (Leung & Zernicka-Goetz, 2013; Feng *et al*, 2014; Taniguchi *et al*, 2015), one can argue that the pro-proliferative role of YAP in cervical cancer cells may be independent of the Hippo pathway. However, our present studies clearly indicated that the Hippo pathway is involved in the YAP regulation of cervical cancer cell growth. First of all, the results obtained from the multidimensional cancer genomic datasets clearly indicated that in the cervical cancer tissue, the Hippo pathway is frequently disrupted, indicated by the frequent deletion and mutation of the genes involved in the Hippo pathway (FigEV1A). As a consequence of the disrupted Hippo pathway, YAP, TAZ, and TEADs are frequently up-regulated in cervical cancer tissues. Secondly, both TGF-α and AREG dephosphorylate MOB1, LATS1, and YAP in cervical cells (Figs6 and 7), suggesting that the Hippo pathway is actively involved in the EGFR pathway regulation of cervical cancer progression. Finally, knockdown of LATS1/2, the major suppressors of YAP, results in dephosphorylation of YAP in ME180 cervical cancer cells, leading to the significant increase in the cell proliferation and AREG secretion in both 2D and 3D culture systems. These results provide compelling evidence that the Hippo pathway is actively involved in the regulation of cervical cancer initiation and progression.

The causal relationship between high-risk HPV infection and cervical cancer has been proposed because high-risk HPVs, such as HPV16, HPV18, and HPV31, have been detected in up to 99.7% of cervical squamous cell carcinomas and 94–100% of cervical adeno- and adenosquamous carcinomas (Walboomers *et al*, 1999; Castellsague *et al*, 2006). Two types of high-risk HPVs, HPV16 and HPV18, have been proposed to be responsible for more than 70% of all cervical cancer cases (Schiffman *et al*, 2007). It is believed that the high-risk HPV oncoproteins, E6 and E7, contribute to cervical carcinogenesis by inactivating the cellular tumor suppressor proteins p53 and pRb, respectively (Dyson *et al*, 1989; Scheffner *et al*, 1990; Boyer *et al*, 1996). However, recent studies showed that although persistent infection with high-risk HPV is a necessary step for the development of cervical cancer, HPV alone is not sufficient to initiate and drive the progression of cervical cancer (Melnikow *et al*, 1998; Reshmi & Pillai, 2008). Moreover, evidence shows that HPV E6-induced degradation of P53 may have no role on cell transformation. For example, it has been shown that mutants of E6, defective in their ability to induce the degradation of p53, can still immortalize human embryonic cells (Ishiwatari *et al*, 1994; Nakagawa *et al*, 1995). E6 proteins from HPV-5 and HPV-8 do not interact with P53 protein, but they are necessary for immortalization of rodent fibroblasts (Elbel *et al*, 1997). On the contrary, E6 protein of the low-risk HPV-1 inhibits P53 transactivation (Kiyono *et al*, 1994). Therefore, the exact molecular mechanism for HPV to drive the initiation and progression of cervical cancer is not clear. Other functions of E6 must also be involved in this process.

In the present study, for the first time, we show that the dysregulation of the Hippo pathway may be involved in the HPV-induced initiation and progression of cervical cancer. We found that HPV E6 protein is able to increase the level of YAP, the major effector of the Hippo pathway, in cervical cancer cells by preventing YAP from proteasome-dependent degradation. Regulation of protein turnover by ubiquitin-mediated protein degradation has long been recognized as an important mechanism for regulating the activity of signal transduction pathways in cancer (Marmor & Yarden, 2004; Fulda *et al*, 2012). Recent studies suggested YAP is inactivated by two mechanisms: (i) the Ser127 phosphorylation-mediated spatial regulation (nuclear–cytoplasmic shuttling); and (ii) the Ser397 (Ser381 in YAP protein isoform 2) phosphorylation-mediated temporal regulation (the phosphodegron-induced degradation). These two mechanisms function coordinately to suppress YAP oncogenic activity (Zhao *et al*, 2010; Mo *et al*, 2014). The CK1δ/ɛ and SOCS6 proteins have been reported to be critical for YAP degradation (Zhao *et al*, 2010; Hong *et al*, 2014). However, expression of HPV E6 in HT3 cells induced a great increase in CK1δ/ɛ and a drastic decrease in p-YAP (S397), suggesting that CK1δ/ɛ pathway may not contribute to the HPV E6-mediated YAP protein turnover. However, expression of HPV16 E6 in HT3 cells reduced SOCS6 protein levels, but increased YAP protein levels, suggesting that SOCS6 may be involved in HPV E6-mediated YAP protein stabilization in cervical cancer cells. Interestingly, one recent report showed that the small t antigen of polyomavirus (PyST) stabilizes YAP through promoting its dephosphorylation (Hwang *et al*, 2014), suggesting that different viruses may stabilize YAP protein in different ways. More experiments are required to uncover the molecular mechanisms underlying HPV oncoprotein stabilizing YAP protein. Importantly, the interactions between the Hippo pathway and other high-risk HPV oncoproteins (such as E5 and E7) may also play an important pathological role in cervical carcinogenesis.

Finally, our data also show that HPV E6 not only increased YAP protein levels, but also increased the expression of *AREG* mRNA and the secretion AREG protein in culture medium. These observations suggest that HPV E6 protein may contribute to the Hippo-ERBB positive signaling loop to drive the transformation and progression of cervical cancer. Interestingly, previous studies have shown that HPV16 E6 and E7 can transcriptionally activate EGFR expression (Sizemore *et al*, 1998; Akerman *et al*, 2001). The HPV16 E6 causes prolonged receptor protein tyrosine kinase signaling and enhances internalization of phosphorylated receptor species (Spangle & Munger, 2013). Internalized receptors maintain signaling potential since they are phosphorylated at residues that promote receptor association with signaling adaptor proteins and cause activation of downstream signaling cascades (Sigismund *et al*, 2008; Goh *et al*, 2010). Collectively, these findings suggest that the Hippo-ERBB positive signaling loop may play an important role in mediating HPV action during the initiation and progression of cervical cancer.

In summary, our study demonstrates that YAP is overexpressed in human cervical cancer tissues and its expression is correlated with cervical cancer progression. *In vitro* and *in vivo* findings support the idea that the Hippo/YAP signaling pathway plays a critical role in the progression of cervical cancer. We also found that the Hippo pathway interacts with the ERBB signaling pathway to form a positive feedback signaling loop to regulate cervical cancer progression (Fig12). Most importantly, HPV16 E6, via preventing proteasome-dependent degradation of YAP, maintains the level of YAP protein in the cervical cancer cells, which may serve to drive cancer cell growth. Therefore, YAP might be a promising prognostic biomarker for cervical cancer and a novel target for the development of drugs against cervical cancer.

## Materials and Methods

### Chemicals

Transforming growth factor alpha *(*TGF-α), EGF, amphiregulin (AREG), HPV16 E6, and ELISA kit of AREG were from R&D systems Inc. (Minneapolis, MN). McCoy’s 5a and other cell culture media were from Invitrogen (Carlsbad, CA). Fetal bovine serum (FBS) was from Atlanta Biologicals, Inc. (Lawrenceville, GA). The Ribogreen RNA Quantification Kit and Alexa-conjugated secondary antibodies were from Life Technologies Corp. (Grand Island, NY); RNeasy Mini Kit was from QIAGEN Inc. (Valencia, CA). YAP siRNA, EGFR siRNA, LATS1 siRNA, LATS2 siRNA, SOCS6 siRNA#1, and HPV18 E6 siRNA were from Dharmacon/Thermo Scientific (Pittsburgh, PA). Lentiviral-based shYAP#1, shYAP#2, and shLATS1/2 vectors were from Addgene (Cambridge, MA). Lentivirus containing HPV16 E6 gene was from Applied Biological Materials (ABM) Inc. (Richmond, BC, Canada). PCR chemicals were from Invitrogen (Carlsbad, CA), QIAGEN (Carlsbad, CA), or Bio-Rad (Hercules, CA). Antibodies against YAP, phospho-YAP (Ser127), phospho-YAP (Ser397) LATS1, phospho-LATS1, MOB1, phospho-MOB1, EGFR, phospho-EGFR, ERK1/2, phospho-ERK-1/2, AKT, phospho-AKT, and β-TrcP were from Cell Signaling Technology Inc. (Danvers, MA); siRNA of SOCS6 and antibodies against SOCS6 and CKIε were from Santa Cruz (Dallas, Texas). Antibodies against β-actin were from Sigma-Aldrich (St. Louis, MO). Peroxidase-conjugated secondary antibodies for Western blot analysis were from Jackson ImmunoResearch Laboratories Inc. (West Grove, PA); the SuperSignal West Femto Chemiluminescent Substrate Kit was from Pierce/Thermo Scientific (Rockford, IL); Optitran nitrocellular transfer membrane was from Schleicher & Schuell Bioscience (Dassel, Germany); MG132, cycloheximide, AG1478, LY294002, U0126, and SB203580 were from EMD Millipore Corporation (Billerica, MA). All other molecular-grade chemicals were purchased from Sigma (St. Louis, MO), Fisher (Pittsburgh, PA), or United States Biochemical (Cleveland, OH).

### Cell lines and human cervical tissue array

ME180, HT3, Ect1/E6E7, HeLa, and End1/E6E7 cells were purchased from ATCC (Manassas, VA). ME180 cells were derived from an omental metastasis of a recurrent cervical epidermoid carcinoma (Sykes *et al*, 1970). HT3 cells were derived from tumor cells of a cervical papilloma patient that recurred from the cervix and lymph nodes (Fogh *et al*, 1977). Ect1 and End1/E6E7 cell lines were established from normal epithelial tissue taken from a premenopausal woman undergoing hysterectomy for endometriosis (Fichorova *et al*, 1997; Fichorova & Anderson, 1999). Cells used in these experiments were from passages 6–12. All cell lines were validated by short tandem repeat (STR) polymorphism analysis performed by the Genetica DNA Laboratories (Burlington, NC, USA). The cervical carcinoma and normal tissue arrays, with stage and grade information, were purchased from US Biomax (Rockville, MD). This array contained 25 early-stage cases, 45 advanced-stage cases, and 10 normal tissues.

### Detection of YAP expression in human cervical cancer and normal tissues by immunohistochemistry

YAP protein expression in human cervical tissues was detected by using peroxidase-based immunohistochemistry as described previously (Fu *et al*, 2014). Briefly, human tissues were deparaffinized with xylene, rehydrated with graded ethanol series, and then autoclaved in an unmasking solution (Vector Laboratories, Burlingame, CA) for antigen retrieval before blocking endogenous peroxidase activity with 3% hydrogen peroxide. Tissues were next blocked with 10% normal donkey serum (NDS) at room temperature for 1 h followed by incubation with primary antibodies at 4°C for 16 h. Biotinylated secondary antibody and streptavidin peroxidase complex (DAKO LSAB Kit, Carpinteria, USA) were added consecutively for ten minutes at room temperature. The immunosignal was visualized with DAB kit (Invitrogen, Carlsbad, CA). The sections were counterstained with Mayer’s hematoxylin. For negative controls, the primary antibody was replaced with blocking buffer containing the same amount of IgG from the non-immune rabbit serum. Sections were scanned with an iSCAN Coreo Slide Scanner (Ventana Medical Systems, Inc. Oro Valley, AZ). The intensity of the positive immunosignal was quantified using Aperio ImageScope software (Vista, CA). The intensity of positive signal and the positivity (i.e., the ratio of positive cell number relative to the total cell) of each section were recorded.

### Western blot analysis

Western blot was used to determine protein levels as described previously (Wang *et al*, 2013). Briefly, normal or treated cells were harvested on ice with ice-cold lysis buffer containing 10 mM Tris pH 7.4, 100 mM NaCl, 1 mM EDTA, 1 mM EGTA, 1 mM NaF, 20 mM Na_4_P_2_O_7_, 1% Triton X-100, 10% glycerol, 0.1% SDS and 0.5% deoxycholate, and protease and phosphatase inhibitor cocktails. Samples (30 μg protein) were loaded to a 10% SDS–PAGE gel, fractioned through electrophoresis, and transferred onto nitrocellulose membranes. The membranes were blocked with 5% BSA and then probed with appropriate primary and horseradish peroxidase (HRP)-conjugated secondary antibodies. The immunosignal was detected using a Thermo Scientific SuperSignal West Femto Chemiluminescent Substrate Kit. The images were captured and analyzed using a UVP gel documentation system (UVP, Upland, CA).

### Quantitative real-time PCR

Quantitative real-time PCR (qRT–PCR) was used to determine mRNA expression. Total RNA was prepared with TRIzol reagent (Invitrogen, Carlsbad, CA) and QIAGEN RNeasy mini kit (QIAGEN, Carlsbad, CA). RNA concentration was determined with Ribogreen RNA quantification kit. Reverse transcription was done by using high-capacity cDNA reverse transcription kit (Applied Biosystems, Grand Island, NY). PCR was performed in a Bio-Rad CFX96 real-time fast PCR system. The following primers were used for AREG: forward: 5′-CGAACCACAAATACCTGGCTA-3′; reverse: 5′-TCCATTTTTGCCT CCCTTTT-3′. The following primers were used for 18S (used as a loading control): forward: 5′-GGCGTCC CCCAACTTCTTA-3′; reverse: 5′-GGGCATCACAGACCTGTTATT-3. All samples were amplified in triplicate.

### Establishment of YAP-overexpressing and knockdown cell lines

Nine cell lines expressing different levels of YAP protein were established to determine the role of YAP in cervical cancer cell proliferation. Briefly, ME180, HT3, and Ect1/E6E7 cells were cultured to 40% confluent and then transfected with retrovirus-based YAP overexpression constructs. The efficiency of these vectors has been reported previously (Dong *et al*, 2007). The following nine stable cell lines were established: (i) ME180-MXIV, HT3-MXIV, and Ect1-MXIV control cell lines were transfected with the empty control vector MXIV and express endogenous YAP; (ii) the ME180-YAP, HT3-YAP, and Ect1-YAP cell lines were transfected with a vector overexpressing wild-type YAP protein; and (iii) the ME180-YAP^S127A^, HT3-YAP^S127A^, and Ect1-YAP^S127A^ cell lines expressing a constitutively activated YAP mutant. Notably, mutation of YAP protein (serine to alanine at residue 127) prevents YAP phosphorylation, leading to its nuclear localization and constitutive activation. Stable YAP- and YAP^S127A^-expressing clones were selected using G418. YAP expression was confirmed by Western blot and RT–PCR analysis. The effect of YAP on cervical cancer cell proliferation was also determined by knockdown of YAP protein using the RNA interference technique. Briefly, 60% confluent ME180 cells were transfected with siNeg (a cy5-labeled non-target siRNA as control) or YAP siRNAs for 6 h using Lipofectamine 2000 (Invitrogen, Carlsbad, CA). Flowing the previous report (Rosenbluh *et al*, 2012), lentiviral-based shYAP#1 and shYAP#2 were used to establish two cell lines with YAP knockdown. Successful knockdown of YAP was also confirmed by RT–PCR and Western blot.

### Cell proliferation, cell migration, and colony formation assays

Cell proliferation was determined by counting cell number with an Invitrogen Countess® automated cell counter (Carlsbad, CA) and detecting cell cycle progression with flow cytometry. Cell migration was determined using wound-healing assay with a protocol established in our laboratory (Wang *et al*, 2012a). The ability of anchorage-free cell growth was assessed with soft agar cell colony formation assay using CytoSelect 96-Well Cell Transformation Assay kit (Cell Biolabs, Inc.).

### *In vivo* tumorigenicity

Animal handling and all experimental procedures were approved by the Institutional Animal Care and Use Committee (IACUC) of the University of Nebraska Medical Center. ME180-YAP and ME180-YAP^S127A^ cells (6 × 10^6^ cells suspended in 0.1 ml PBS) were injected subcutaneously into the left dorsal flank of 6-week-old female athymic nude mice. ME180-MXIV cells with control vectors were injected into right dorsal flank of these mice. The tumor diameter was measured and documented every 4 days. The tumor volume (mm^3^) was estimated by measuring the longest and shortest diameter of the tumor and calculating as follows: volume = (shortest diameter)^2^ × (longest diameter) × 3.14/6. All mice were euthanized 4 weeks after tumor cell inoculation. Tumors were collected, weighed, and processed for preparation of frozen sections, protein and RNA. Ki67 expression in the tumor xenografts was used as a cell proliferation index and was examined using fluorescent immunohistochemistry with a protocol described previously (Fu *et al*, 2014). Similarly, ME180-CTRL and ME180-shYAP#1 cells (5 × 10^6^ cells suspended in 0.1 ml PBS) were injected subcutaneously into the left or right dorsal flank of 6-week-old female athymic nude mice, and all mice were euthanized 1 month after tumor cell inoculation.

### Examination of the expression of YAP in K14E6/E7 transgenic mice and HPV16-contained organotypic “3D raft” cultures

Control (wild-type), HPV16 E6, and HPV16 E6/E7 cervical cancer mouse model has been reported previously (Brake & Lambert, 2005; Shai *et al*, 2007). Briefly, the keratin-14 (K14) promoter was used to direct E6 or E6/E7 expression in mice. All control K14E6 and K14E6/E7 transgenic mice were treated with estrogen for 6 months. Cervical cancer was observed in KE6 and K14E6/E7 mice (Brake & Lambert, 2005; Shai *et al*, 2007). Normal and cancerous cervical tissues from control and estrogen-induced K14E6 and K14E6/E7 mice were harvested and processed for preparation of paraffin section with the protocol described previously (Brake & Lambert, 2005).

Organotypic cultures were generated as previously described (Lambert *et al*, 2005; Zehbe *et al*, 2009). Briefly, the immortalized keratinocyte cells (NIKS) were transfected with or without HPV16 DNA, after selection with G418 (Invitrogen) for 4 days, the cells were cultured in fresh complete F medium for additional 3 or 4 days. Following the guiding line to prepare dermal equivalent (rafts) and plate Keratinocytes onto the rafts, each raft culture was initiated for 2 × 10^5^ NIKS cells and then cultured this organotypic “raft” for another 16 days. The cultures were fixed in 10% buffered formalin and embedded in paraffin. The expression and localization of YAP protein in organotypic cultures and normal and cancerous mouse cervical tissues were determined by using alkaline phosphatase-based VECTASTAIN® ABC-AP kit (Cat. No. AK-5000). Vector® Red Substrate kit (Cat. No. AK-5100) (Vector Laboratories, Burlingame, CA) was used to visualize immunosignal of YAP protein. Sections were scanned with an iSCAN Coreo Slide Scanner (Ventana Medical Systems, Inc. Oro Valley, AZ).

### Human cervical cancer genomic data analysis

Genomic data mining was performed using cBioPortal for Cancer Genomics (available at http://www.cbioportal.org) as described previously (Gao *et al*, 2013). For the analysis of YAP alterations in different cancer types, we selected all 90 available studies. For other analysis associated with cervical cancer, we used cervical cancer (CESC) dataset from The Cancer Genome Atlas (TCGA) Data Portal in accordance with the publication guidelines (TCGA, Provisional). YAP mRNA expression values are converted to z-scores to facilitate comparison and definition of alteration thresholds, and mRNA expression beyond z-score threshold ± 2 would be considered up- or down-regulation (Gao *et al*, 2013). Correlation analysis and network analysis were carried out following a recently described cBioPortal protocol (Gao *et al*, 2013).

### Statistical analysis

All experiments were repeated at least four times unless otherwise noted. Data are presented as mean ± SEM. Statistical analysis was conducted using GraphPad Prism software (GraphPad Software, Inc. La Jolla, CA). Data were analyzed for significance using one-way ANOVA with Tukey’s *post hoc* tests. A value of *P *<* *0.05 was considered statistically significant.

## For more information

Read more on cervical cancer:

http://www.cancer.org/cancer/cervicalcancer/

http://www.cancer.gov/types/cervical

Read more on the Hippo pathway and gynecological cancers:

http://www.unmc.edu/obgyn/research/ocl/index.html

http://www.nature.com/nrc/journal/v13/n4/full/nrc3458.html

## Supporting Information

**Appendix**

**Expanded View Figures PDF**

**Review Process File**
