# Supplementary material for: The Hippo/YAP pathway interacts with EGFR signaling and HPV oncoproteins to regulate cervical cancer progression
Source: EMBO Mol Med. 2015 Sep 28;7(11):1426–49. doi: 10.15252/emmm.201404976 (PMC4644376; doi:10.15252/emmm.201404976)
Supplement: Supplementary file 1 [file emmm0007-1426-sd1.pdf]

Appendix Figures

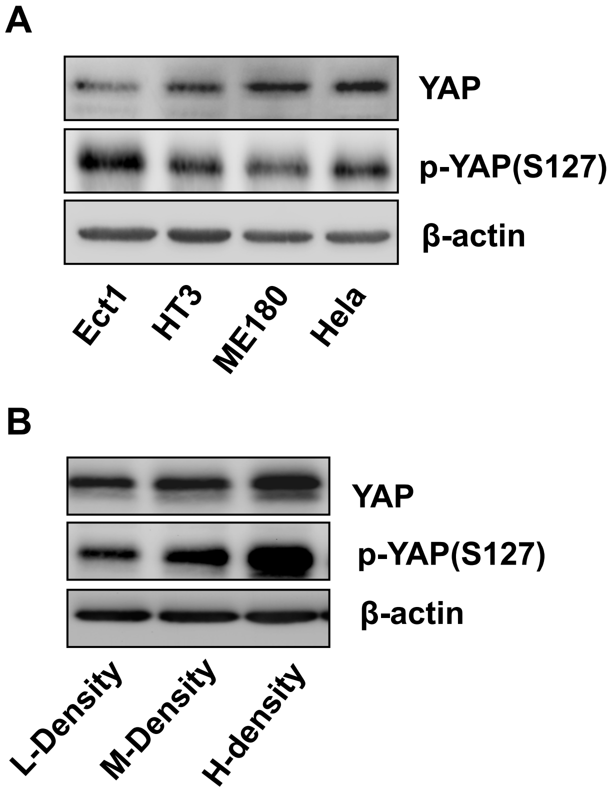

Appendix Figure S1. Expression and activation of YAP protein in normal and cancerous cervical cell lines. **(A)** Western blot detection of total and phosphorylated YAP1 [(p-YAP(S127)] in the Ect1/E6E7 (Ect1), ME180, HT3 and Hela cervical cell lines. **(B)** Western blot determine the cell density-associated YAP1 phosphorylation in ME180 cervical cancer cell line. Experiments were independently repeated for three times. Representative blots were presented. L-Density: Low density cells; M-Density: Moderate density cells. H-density: high density cells. β-actin was used as protein loading control.

Appendix Figures

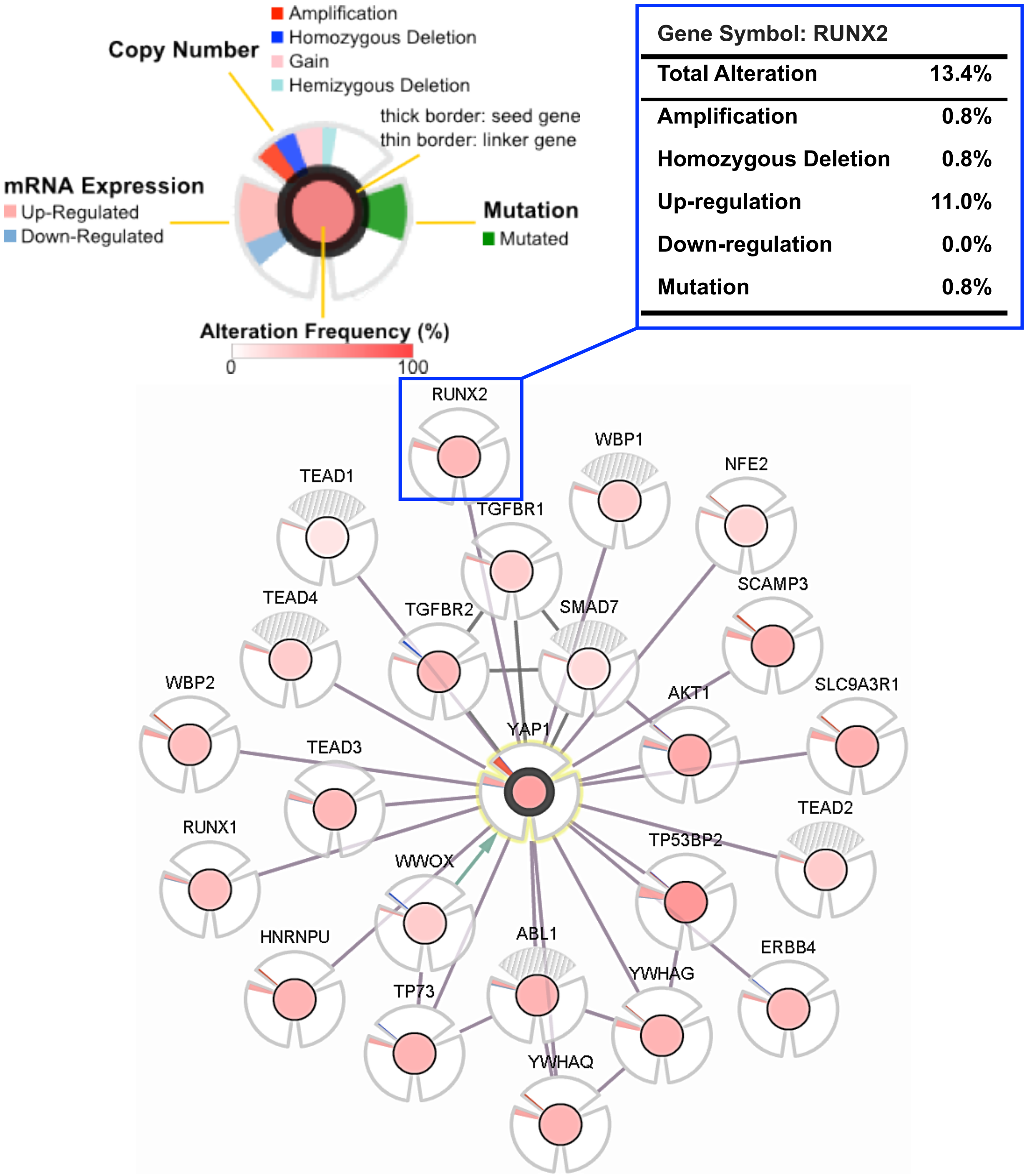

Appendix Figure S2. Network view of alterations of Y*AP1* linker genes in cervical cancer. Network analysis was used to examine alterations of *YAP* linker genes in cervical cancer. YAP is seed gene (indicated by the thick border). All other linker genes are automatically identified once altered in cervical cancer by the analysis software (cBioPortal, available at http://www.cbioportal.org/). The increase in red color gradient indicates the increase of the alteration frequency of a given gene (defined by changes in mutation, copy number amplification, or mRNA expression) in cervical cancer. *RUNX2* gene alteration in cervical cancer is taken as an example for data visualization .

Appendix Figures

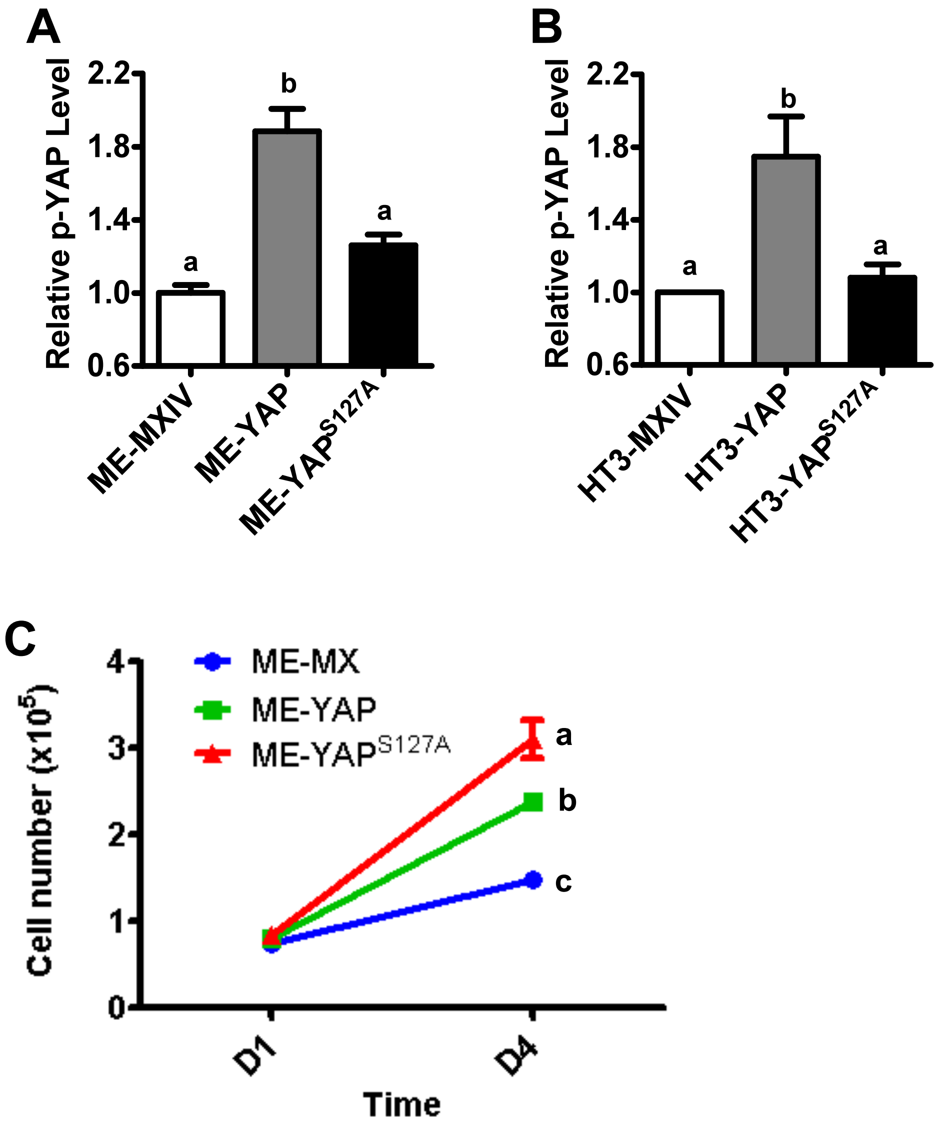

Appendix Figure S3. Effect of YAP on the proliferation of normal and cancerous cervical cells. (A &B) Quantitative data of phosphorylation YAP (Ser127) for Figure 2a &2c. Each bar represents the mean ± SEM of three independent experimental results. Bars with different letters are significantly different from each other (ME-MXIV VS. ME-YAP, P = 0.0013; HT3-MXIV VS. HT3-YAP, P = 0.0139). **(C)** Growth of ME180 derived cervical cancer cells in serum-reduced conditions. ME180-MXIV, ME180-YAP and ME180-YAP^{S127A} cells were incubated with reduced serum (1%). Cell number was counted with an Invitrogen Countness®cell counter. Each point represents mean ± SEM (n = 5). Points with different letters are significantly different from each other (ME-MX VS. ME-YAP, P = 0.0037; ME-MX VS. ME-YAP^{S127A}, P = 0.0007). Quantitative data in (A), (B) & (C) were analyzed for significance using one-way ANOVA in GraphPad Prism 5 with Tukey's post-hoc tests.

Appendix Figures

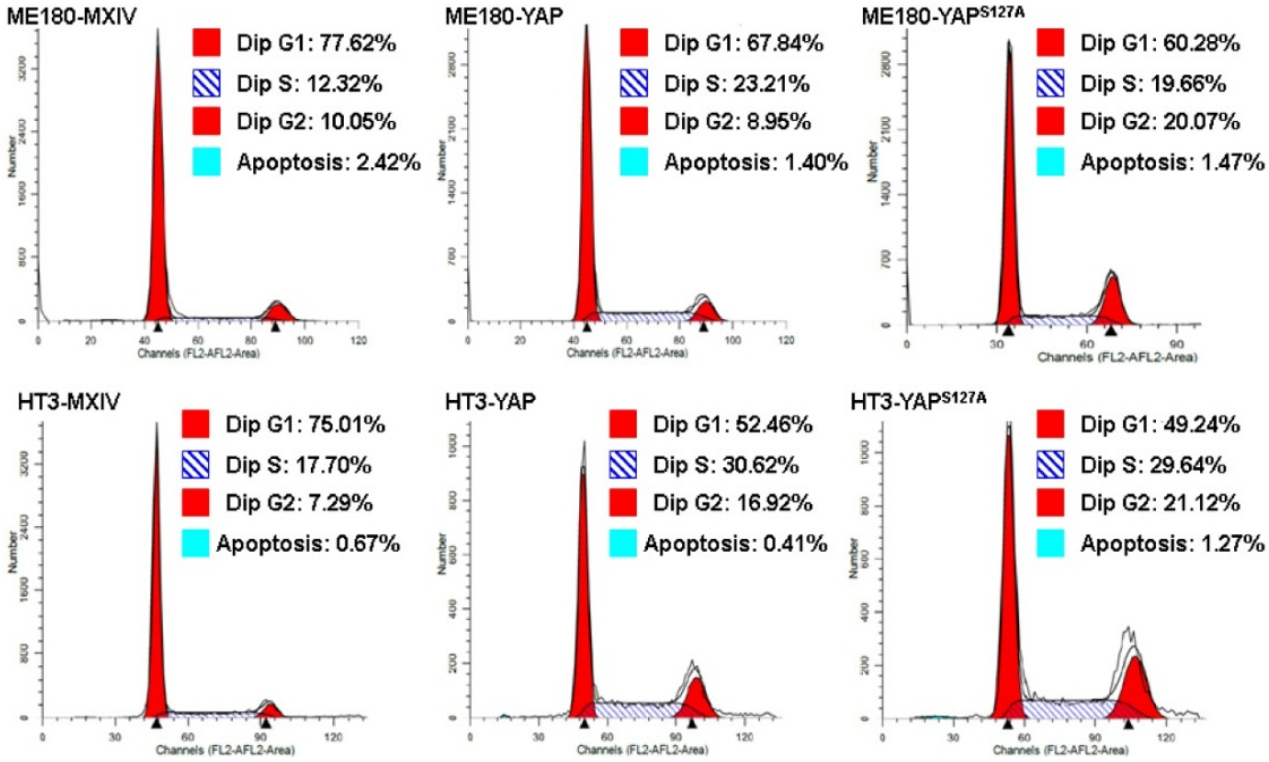

Appendix Figure S4. Effect of YAP on the cell cycle progression in ME-180 and HT3 cervical cancer cells. ME180-MXIV, ME180-YAP, ME180-YAP^{S127A}, HT3-MXIV, HT3-YAP, HT3-YAP^{S127A} cell lines were cultured in 6-well plate with 10% serum. Cell cycle progression was determined by flow cytometry after cell confluent. Results are representatives of five separate experiments. This study showed that overexpression and/or activation of YAP1 enable cervical cancer cells to overcome contact inhibition-induced slowdown of cell proliferation.

Appendix Figures

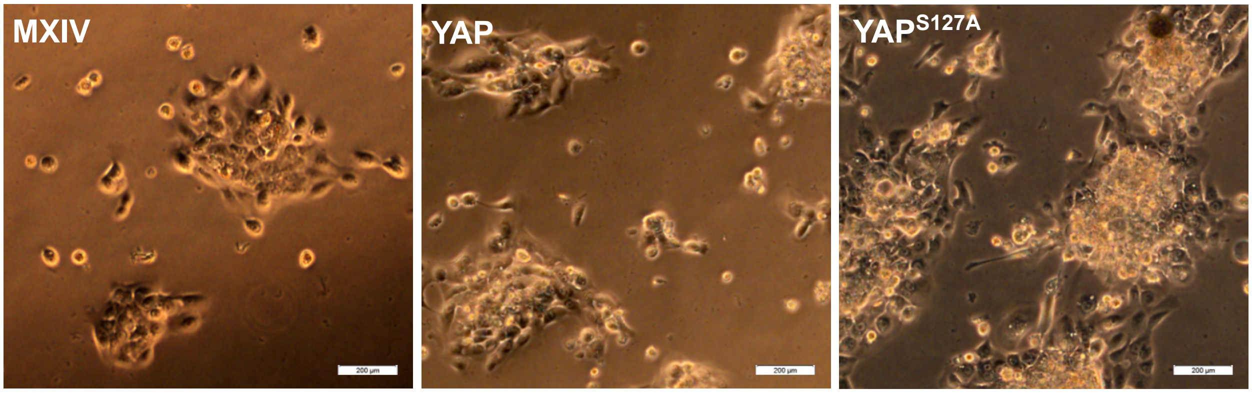

Appendix Figure S5. Effect of YAP on the morphology of Ect1 cells. Ect1/E6E7 cervical epithelial cells were transfected with empty control vector (MXIV), vectors that express wild-type YAP (YAP) or vectors that express mutated YAP (YAP^{S127A}, a constitutively active form). Three stable cell lines were established by G418 selection. Morphology of Ect1-MXIV, Ect1-YAP and Ect1-YAP^{S127A} cells was recorded after 96h culture in growth medium. Scale bar: 100µm.

Appendix Figures

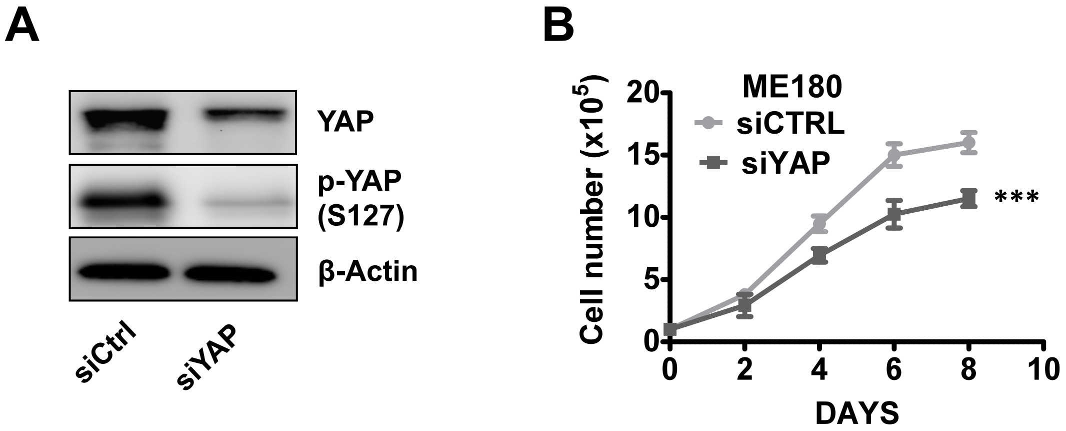

Appendix Figure S6. Effect of YAP on the proliferation of HT3 cervical cancer cells. (A) Western blot analysis of YAP levels in ME180 cells transfected with non-targeting control siRNA (siCtrl) and YAP siRNA (siYAP). (**B**) Growth curves of ME180 cells transfected with non-targeting control siRNA (siCtrl) and YAP siRNA (siYAP). Cells cultured in completed medium with 2.5% serum. Cell number was counted with an Invitrogen Countness®cell counter. . Data were analyzed for significance with unpaired t test in GraphPad Prism 5 with Welch's correctionEach point represents mean ± SEM (n = 5). Points with different letters are significantly different from each other on day 6 (*P* = 0.0027).

Appendix Figures

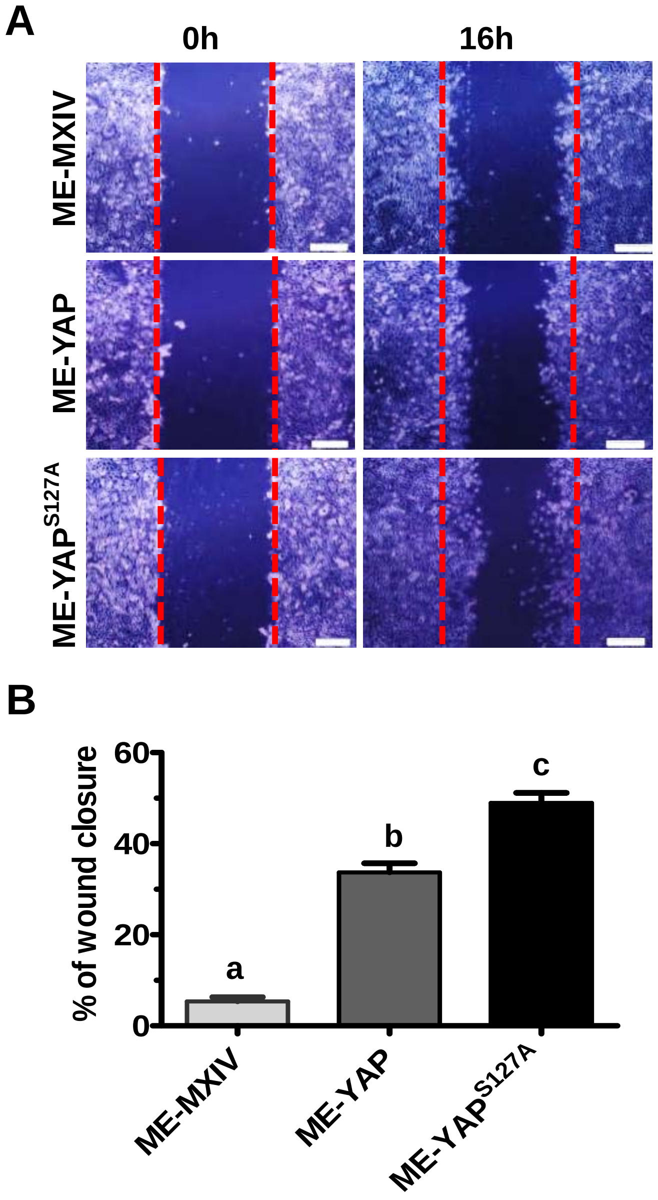

Appendix Figure S7. Effect of YAP on cervical cancer cell migration. A) Representative images showing the motility of ME180-MXIV (control), ME180-YAP1 and ME180-YAP1^{S127A} cells as determined by a wound healing assay. Note the rapid closure (16h) of the "wound" in ME180-YAP1^{S127A}. **B)** Quantification of wound closure data after 16h of incubation. Bars represent means ± SEM (n=5). Bars with different letters are significantly different from each other (ME-MXIV VS. ME-YAP, P < 0.0001; ME-MXIV VS. ME-YAP^{S127A}, P < 0.0001).

Appendix Figures

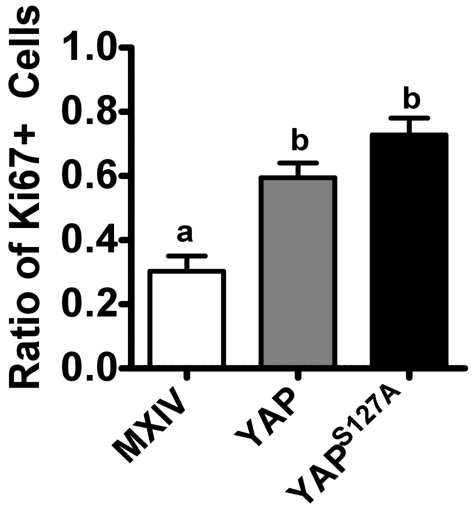

Appendix Figure S8. Effect of YAP on human cervical tumor xenograft growth. Quantitative data (for Figure 4G) showing the percentage of the Ki67 positive cells in tumor xenografts derived from ME180-MXIV (CTRL), ME180-YAP (YAP) and ME180-YAP^{S127A} (YAP^{S127A}) cells. Quantitative data were analyzed for significance using one-way ANOVA in GraphPad Prism 5 with Tukey's post-hoc tests. Each bar represents the mean ± SEM (n = 5). Bars with different letters are significantly different from each other (MXIV VS. YAP, *P* = 0.0044; MXIV VS. YAP^{S127A}, *P* = 0.0010).

Appendix Figures

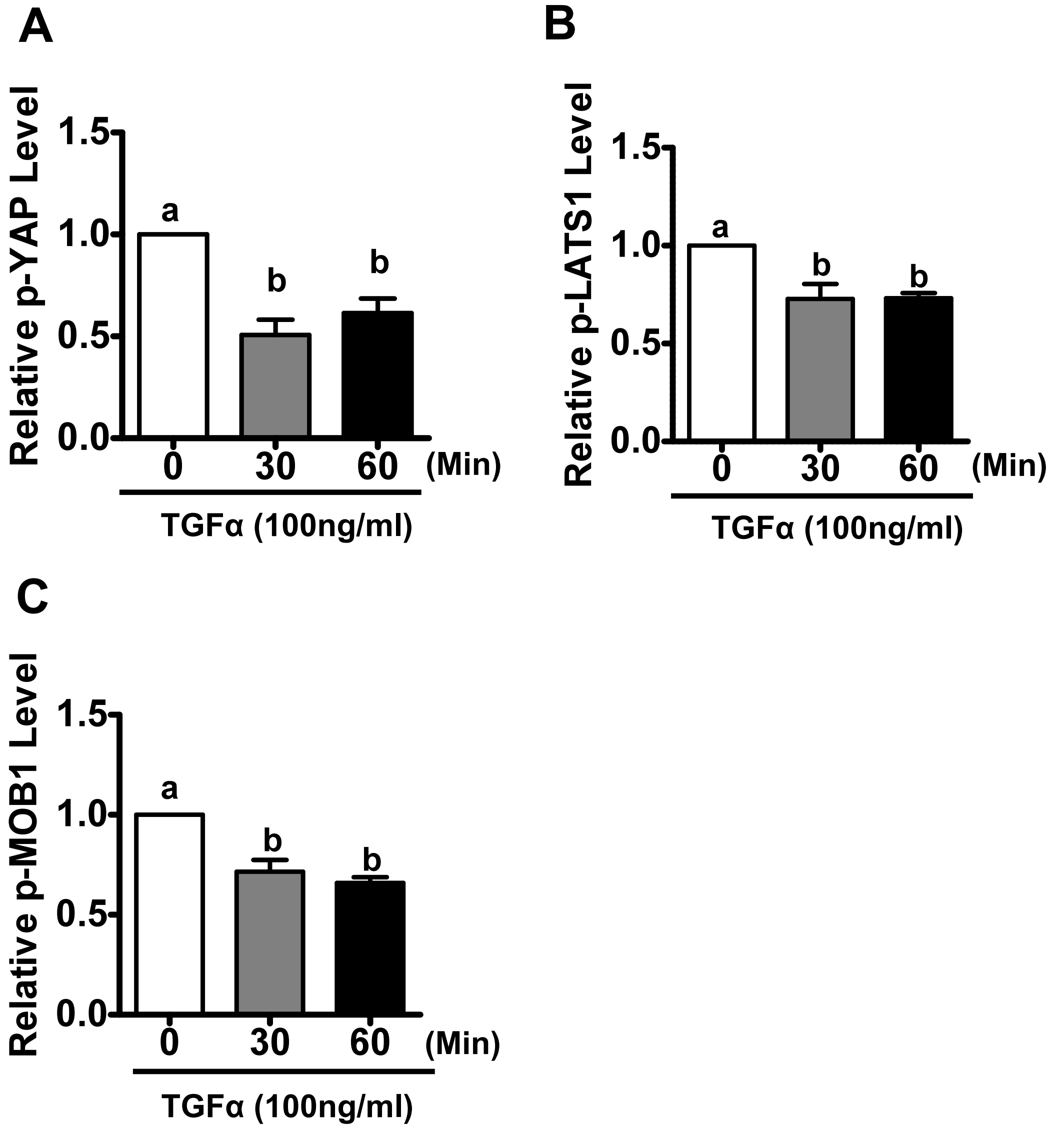

Appendix Figure S9. TGFα regulates the Hippo signaling pathway in cervical cancer cells. Quantitative results showing the relative protein level of (**A**) p-YAP (S127), (**B**) p-LAST1(S909) and (**C**) p-MOB1 (T35) in ME180 cells with or without TGFα treatment for Figure 6B. Quantitative data were analyzed for significance using one-way ANOVA in GraphPad Prism 5 with Tukey's post-hoc tests. Each bar represents the mean OD ± SEM of four independent experiments. Bars with different letters are significantly different from each other (For p-YAP in (A): 0 Min VS. 30 Min, P = 0.0014; 0 Min VS. 60 Min, P = 0.0029). For p-LATS1 in (B): 0 Min VS. 30 Min, P = 0.0062; 0 Min VS. 60 Min, P < 0.0001. For p-MOB1 in (C): 0 Min VS. 30 Min, P = 0.0040; 0 Min VS. 60 Min, P < 0.0001).

Appendix Figures

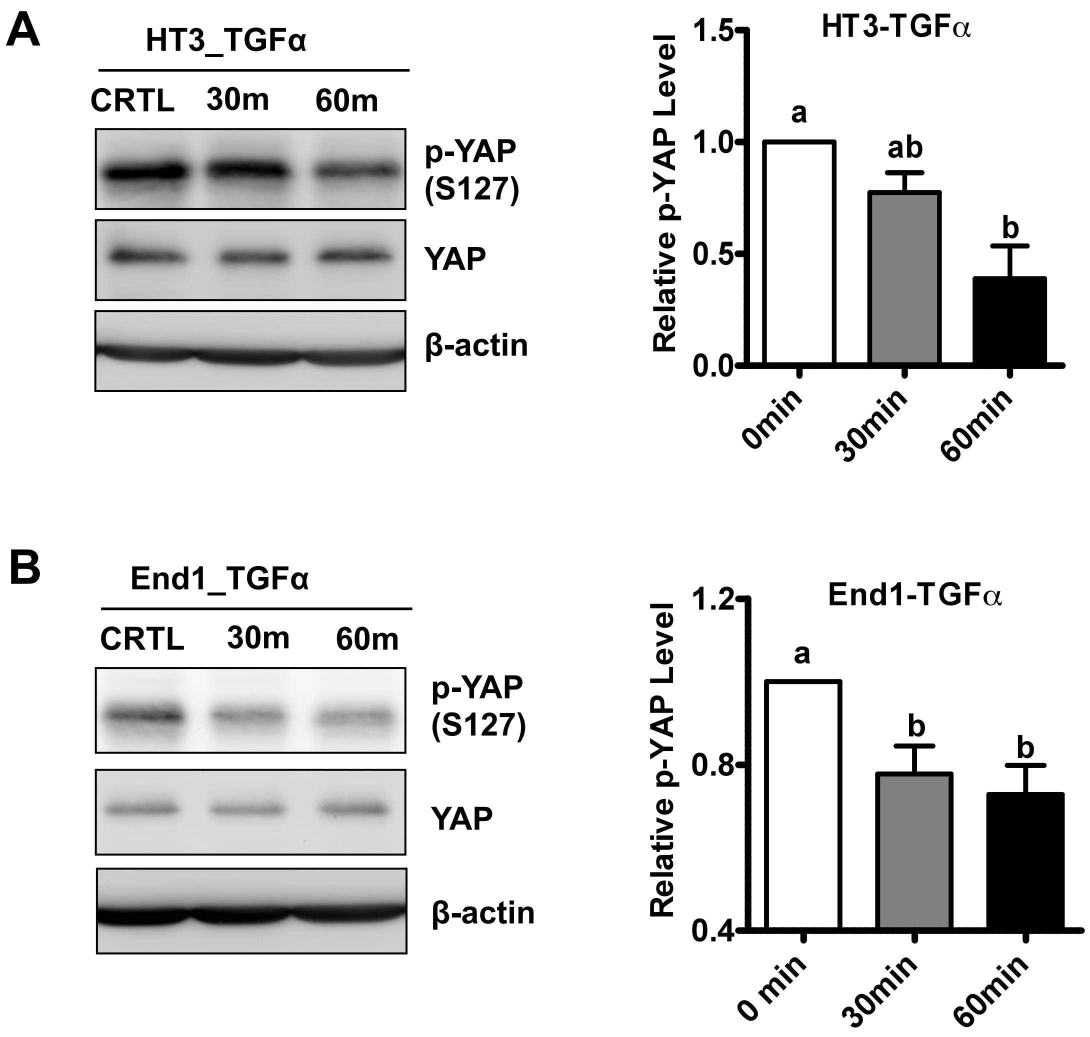

Appendix Figure S10. TGFα regulates YAP activity in HT3 cells and End1 cells. A) Western blot to determine the effect of TGFα (100ng/ml) treatment on the phosphorylation of YAP1 at serine127 in HT3 cervical cancer cells. **B)**) Western blot to determine the effect of TGFα (100ng/ml) treatment on the phosphorylation of YAP1 at serine127 in End1 cells (immortalized cervical cancer epithelial cells). Results showed that TGFα is able to suppressed of YAP1 phosphorylation at serine127 in both HT3 and End1 cervical cells. β-actin was used as protein loading control. Graphs in the right panels are quantitative data of (A) and (B), respectively. Quantitative data were analyzed for significance using one-way ANOVA in GraphPad Prism 5 with Tukey's post-hoc tests. Each bar represents the mean ± SEM of three independent experimental results. Bars with different letters are significantly different from each other (For p-YAP in (A): 0 Min VS. 30 Min, P = 0.0517; 0 Min VS. 60 Min, P = 0.0070. For p-YAP in (B): 0 Min VS. 30 Min, P = 0.0082; 0 Min VS. 60 Min, P = 0.0046).

Appendix Figures

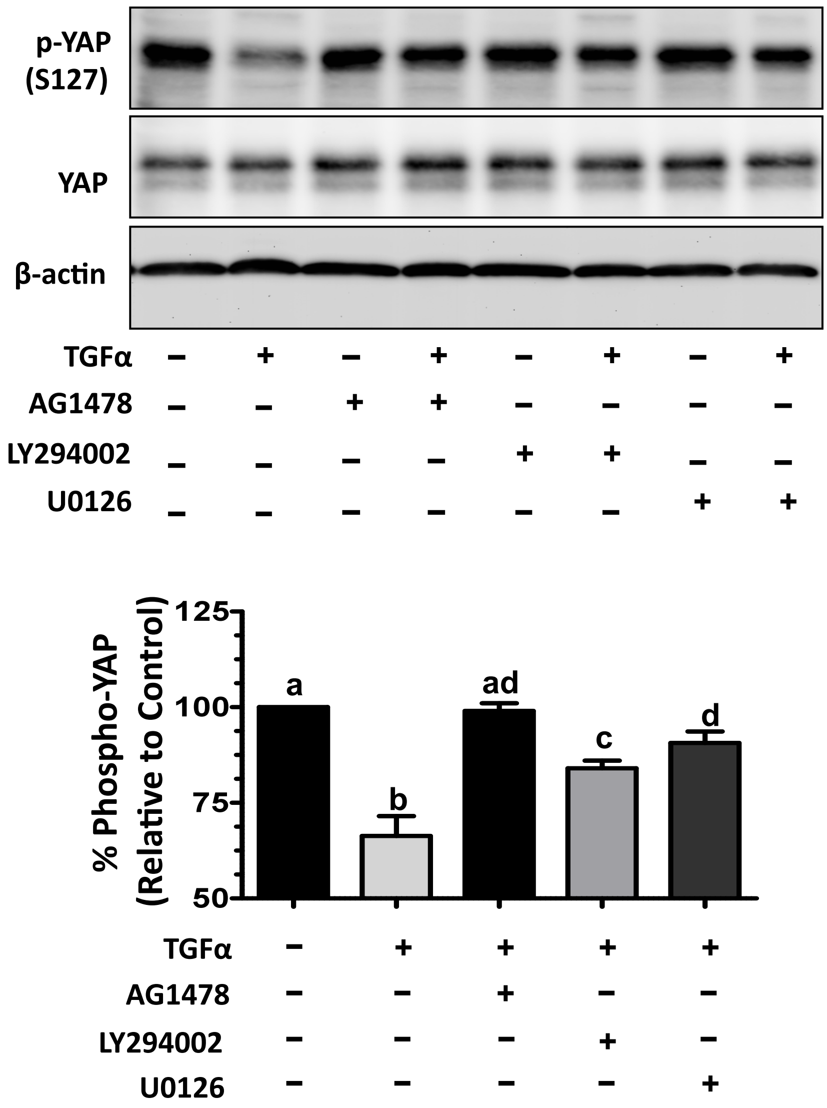

Appendix Figure S11. Involvement of PI3K/AKT pathway and MAPK pathway in TGFα regulation of YAP activation. Top panel: Western blot analysis showing that inhibition of EGFR (AG1478), MEK (UO126) and PI3K (LY294002) signaling by pathway specific inhibitors rescued TGFα-induced suppression of YAP1 phosphorylation. Lower panel: Quantitative data of Western blot result generated from an UVP gel document system. Quantitative data were analyzed for significance using one-way ANOVA in GraphPad Prism 5 with Tukey's post-hoc tests. Each bar represents the mean ± SEM (n = 3). Bars with different letters are significantly different from each other (Control VS. TGFα, P = 0.0029; Control VS. TGFα + AG, P = 0.6422; Control VS. TGFα + LY, P = 0.0013; Control VS. TGFα + UO, P = 0.0345).

Appendix Figures

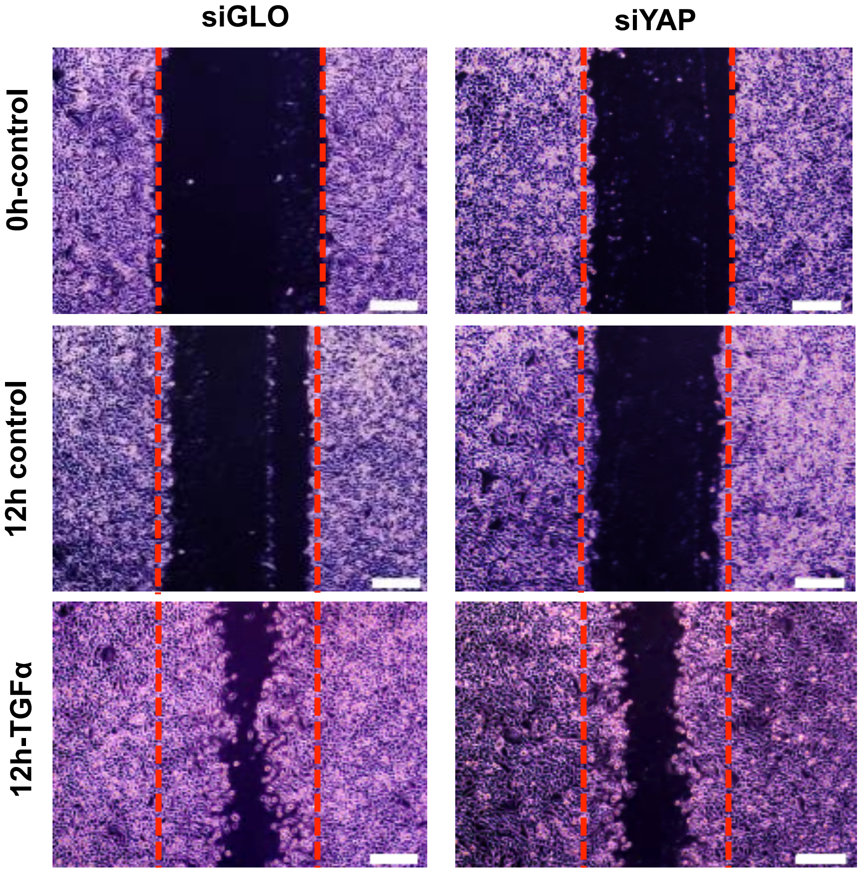

Appendix Figure S12. Involvement of YAP1 in TGFα-stimulated migration of ME180 cervical cancer cells. Wound healing assay was used to determine the role of YAP in TGFα-induced migration of ME180 cervical cancer cells. Confluent control ME180 cells (transfected with non-target control siRNA, siGLO) and YAP-knockdown ME180 cells (transfected with YAP siRNA, siYAP) were scratched with a sterile pipet tip to make a “wound”. Cells were then incubated in serum free medium in the presence or absence of TGFα (10ng/ml) for 12h. The wound closure was recorded under a microscope before and after treatment. Experiments were independently repeated for five times. Representative blots were presented. Knockdown of YAP significantly reduced TGFα-induced wound closure.

Appendix Figures

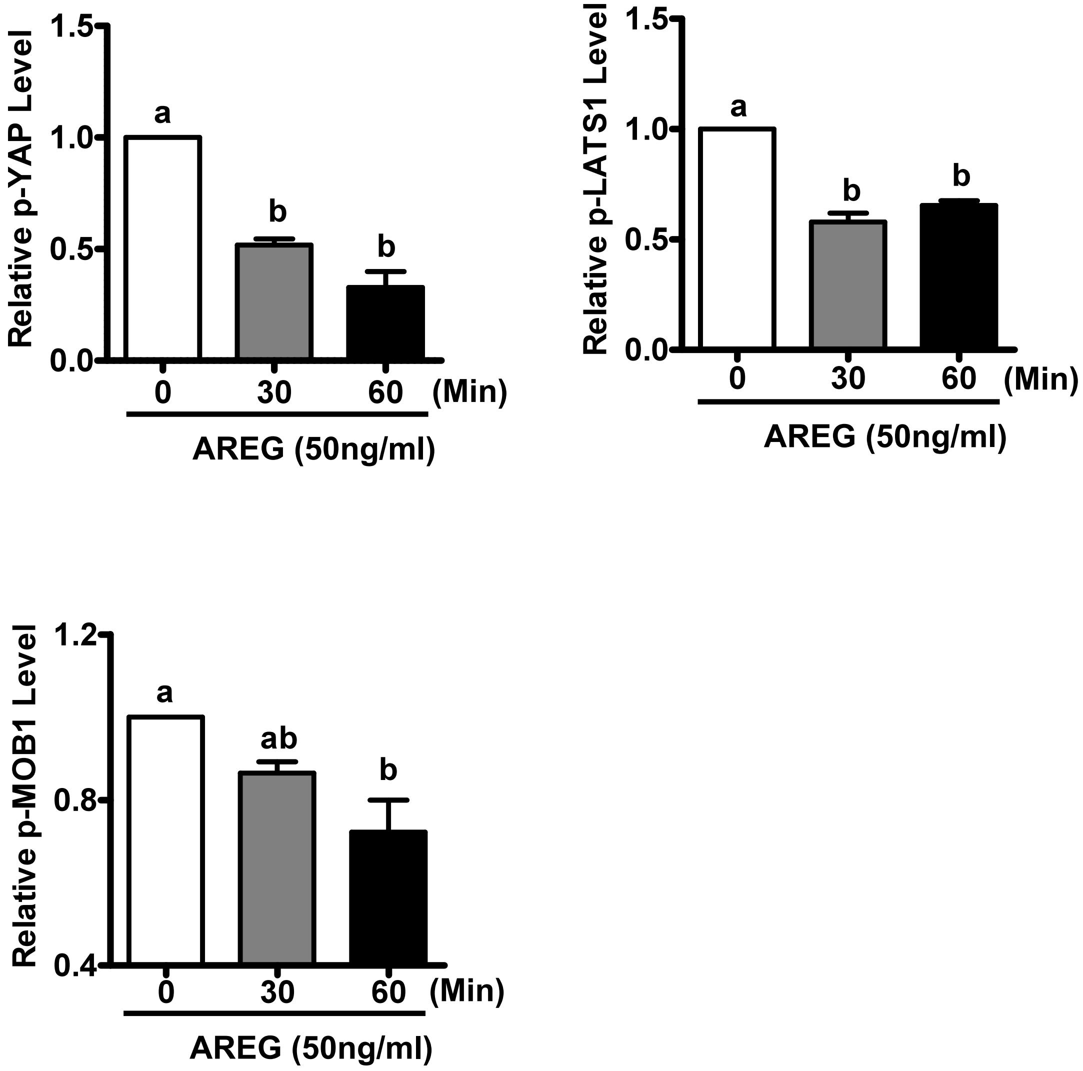

Appendix Figure S13. AREG regulates the Hippo signaling pathway in cervical cancer cells. Quantitative results showing the relative protein level of p-YAP(S127), p-LAST1(S909) and p-MOB1 (T35) in ME180 cells with AREG treatment for Figure 7a. Quantitative data were analyzed for significance using one-way ANOVA in GraphPad Prism 5 with Tukey's post-hoc tests. Each bar represents the mean ± SEM (n = 5). Bars with different letters are significantly different from each other (For p-YAP: 0 Min VS. 30 Min, $P < 0.0001$; 0 Min VS. 60 Min, $p = 0.0004$. For p-LATS1: 0 Min VS. 30 Min, $P < 0.0001$; 0 Min VS. 60 Min, $P < 0.0001$. For p-MOB1: 0 Min VS. 30 Min, $p < 0.1568$; 0 Min VS. 60 Min, $p = 0.0231$).
