# Supplementary material for: The Hippo/YAP pathway interacts with EGFR signaling and HPV oncoproteins to regulate cervical cancer progression
Source: EMBO Mol Med. 2015 Sep 28;7(11):1426–49. doi: 10.15252/emmm.201404976 (PMC4644376; doi:10.15252/emmm.201404976)
Supplement: Supplementary file 2 [file emmm0007-1426-sd2.pdf]

Expanded View Figures

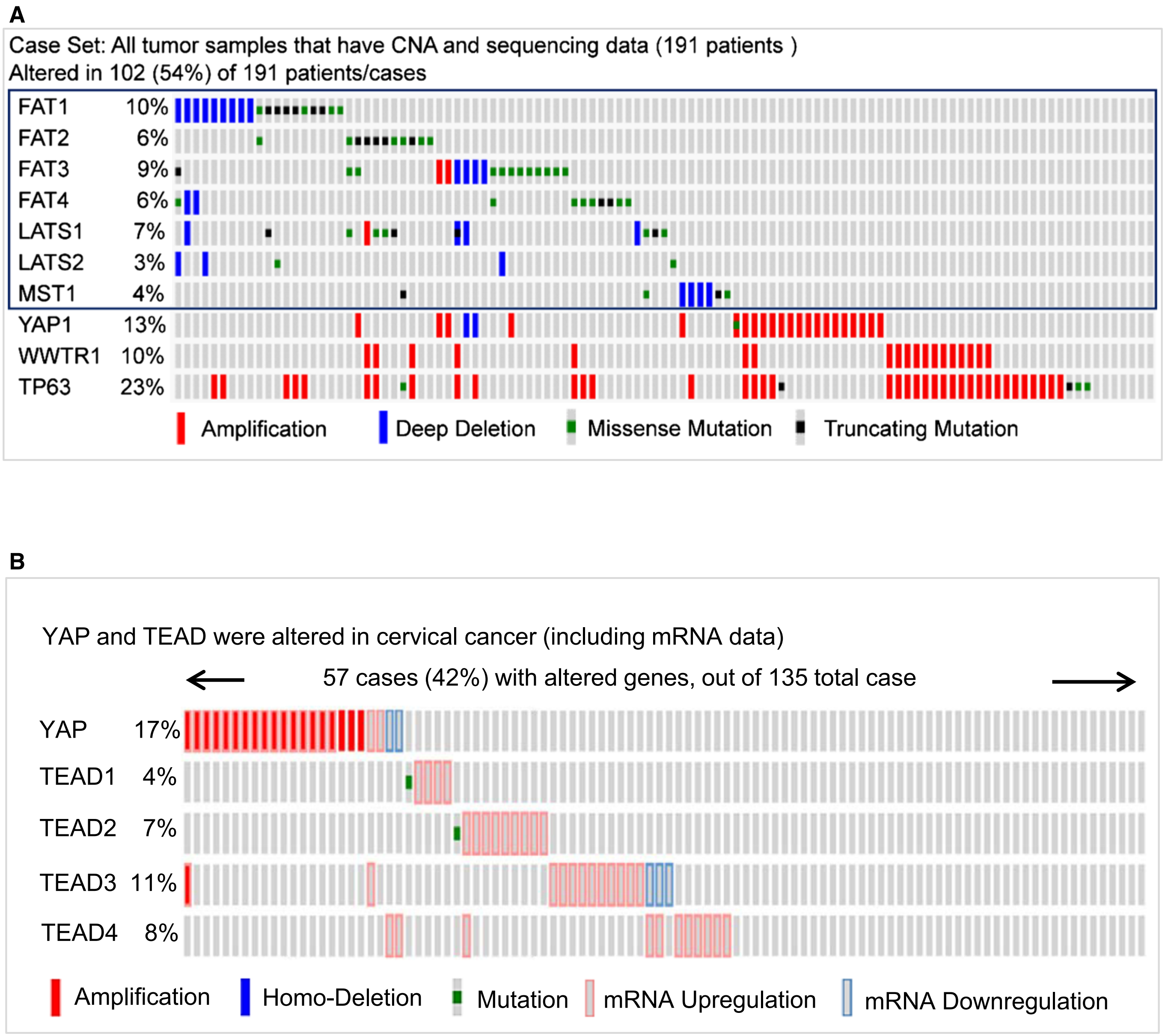

Figure EV1. Multidimensional cancer genomics data analysis showing alteration of the major genes involved in the Hippo/YAP pathway in cervical cancer.

A Alteration frequencies of major genes involved in the Hippo pathway. Genes in the blue box are upstream genes of the Hippo tumor suppressor pathway. Note the frequent deletion and mutation of these genes in cervical cancer. YAP and TAZ (WWTR1) genes are frequently amplified in cervical cancer (n = 191). TP63 are a known cervical cancer biomarker and are frequently amplified in cervical cancer.

B A visual summary of the different mechanisms of YAP1 and TEAD alteration across a set of cervical cancer samples based on a query of the five genes, *YAP*, *TEAD1*, *TEAD2*, *TEAD3*, and *TEAD4*. Each row represents a gene, and each column represents a tumor sample (n = 135). The YAP and TEADs gene alteration analyses were performed using online datasets and data mining tools (the cBioPortal for Cancer Genomics and the datasets from the TCGA Research Network).

© 2015 The Authors

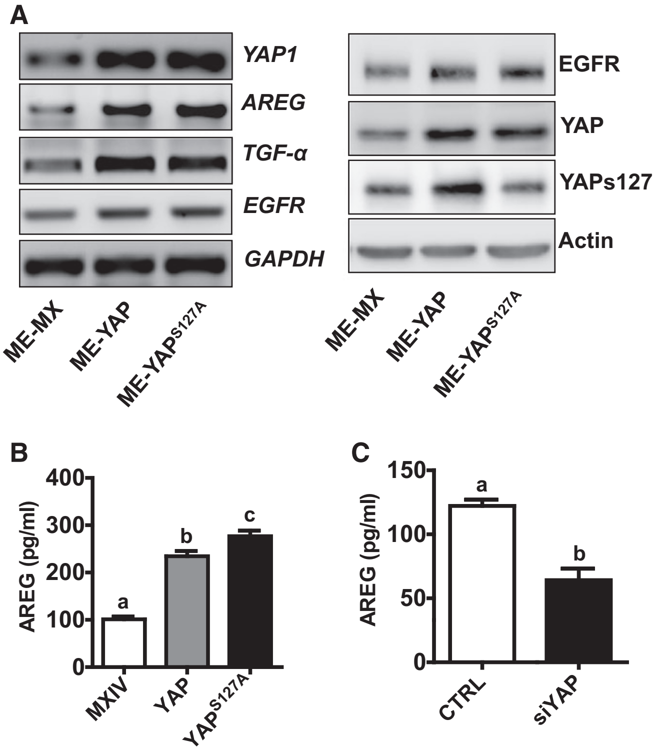

Figure EV2. YAP regulated EGFR, AREG, and TGF-α expression in cervical cancer cell.

A RT–PCR (left) and Western blot (right) were used to determine the expression of EGFR, AREG, and TGF-α in ME180-MXIV, ME180-YAP, and ME180-YAP^{S127A} cells. GAPDH and actin were used as controls.

B The concentration of AREG in cell culture medium from ME180-MXIV, ME180-YAP, and ME180-YAP^{S127A} cells. The concentration of AREG in the medium was determined by AREG ELISA kit. Each bar represents mean ± SEM (n = 5). Bars with different letters are significantly different from each other (ME-MX vs. ME-YAP, $P < 0.0001$; ME-MX vs. ME-YAP^{S127A}, $P < 0.0001$).

C The concentrations of AREG in ME180 cells with or without siYAP treatment. ME180-CTRL and ME180-siRNA cells were incubated in the FBS 1% medium for 48 h. Each bar represents mean ± SEM (n = 5). Bars with different letters are significantly different from each other (P = 0.0006).

Data information: Quantitative data in (B) were analyzed for significance using one-way ANOVA in GraphPad Prism 5 with Tukey's *post hoc* tests. Data in (C) were analyzed for significance with unpaired *t*-test in GraphPad Prism 5 with Welch's correction.

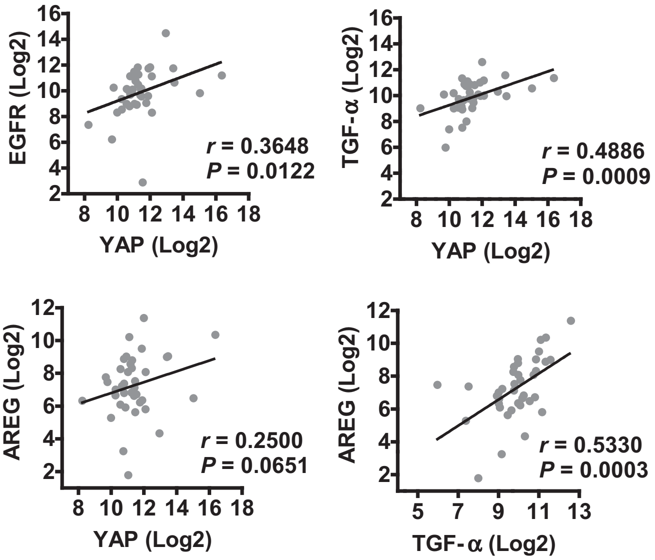

Figure EV3. *YAP* mRNA expression is correlated with TGF-α, EGFR, and AREG in cervical cancer tissues.

mRNA expression data (n = 39) were extracted from TCGA RNA sequencing datasets and were analyzed with linear regression analysis in GraphPad Prism 5 (GraphPad Software, Inc., La Jolla, CA). These data indicate that YAP mRNA expression is significantly correlated with TGF-α (P = 0.001) and EGFR (P = 0.0122) mRNA expression. Statistical analysis shows that YAP mRNA expression is not significantly correlated with AREG mRNA expression (P = 0.065). This may be attributed to the limited cervical cancer sample number used for RNA-seq (n = 39). TGF-α mRNA expression is significantly correlated with AREG mRNA expression in examined cervical cancer samples (P = 0.0003).

© 2015 The Authors

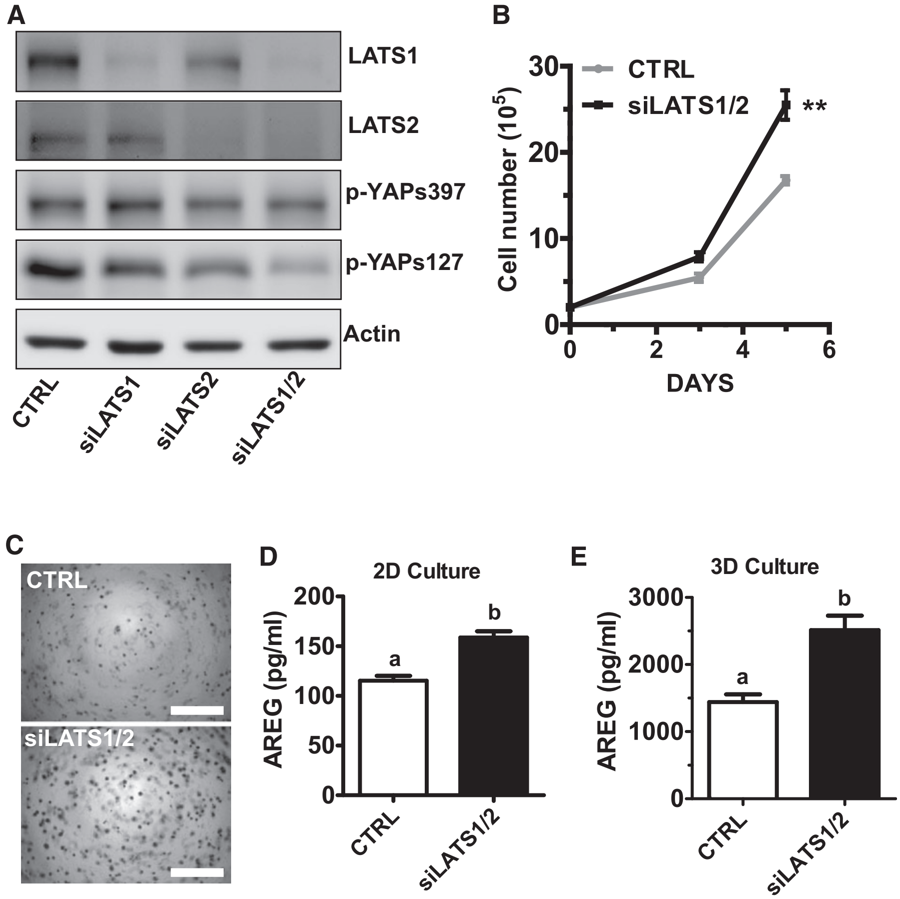

Figure EV4. Effect of LATS1/2 on the growth of cancerous cervical cells.

A Western blot analysis showed that LATS1/2 siRNAs successfully knocked down LATS1/2 and activated YAP in ME180 cells.

B Knockdown of LATS1/2 promoted proliferation of ME180 cells. Each point represents the mean ± SEM of five independent experimental results. $^{**}P < 0.01$ ($P = 0.0020$).

C Representative images showing the anchorage-independent growth of ME180 with or without LATS1/2 knockdown ($n = 5$). Scale bar: 1 mm.

D, E The concentrations of AREG in 2D (D) or 3D hanging-drop (E) culture medium from ME180 control and LATS1/2 knockdown cells. Each bar represents the mean ± SEM of five independent experimental results. Bars with different letters are significantly different from each other [CTRL vs. siLATS1/2 in (D), $P = 0.0017$; CTRL vs. siLATS1/2 in (E), $P = 0.0045$].

Data information: Data in (B), (D), and (E) were analyzed for significance with unpaired *t*-test in GraphPad Prism 5 with Welch's correction.

© 2015 The Authors

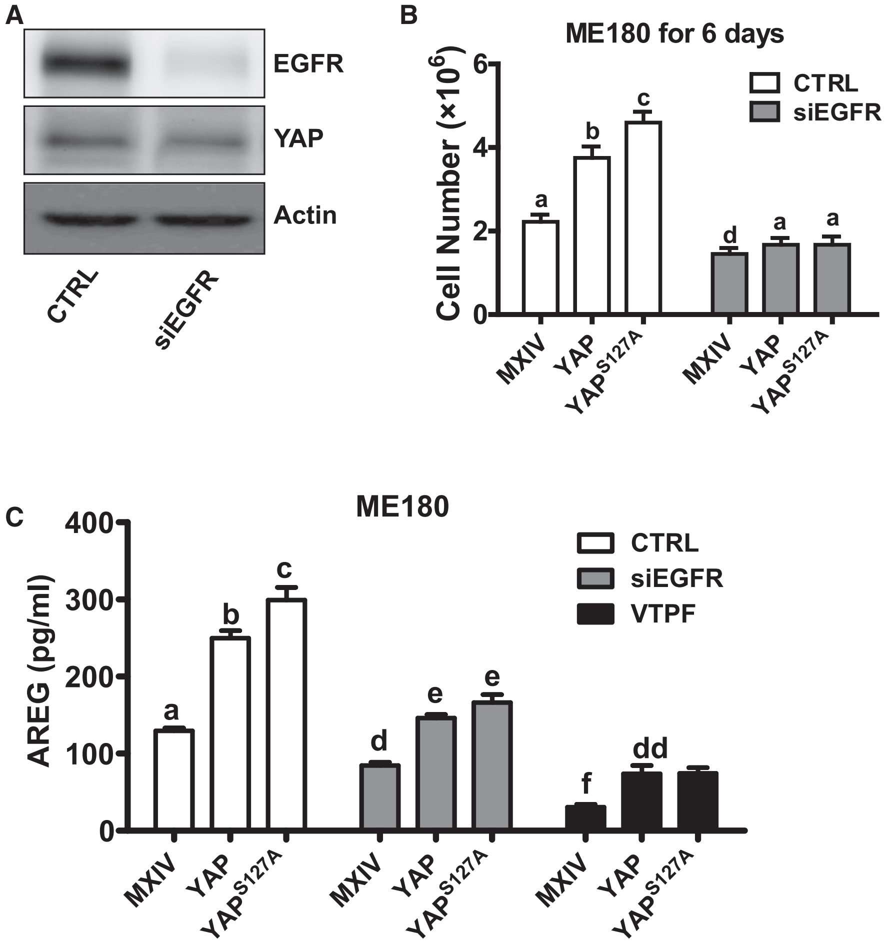

Figure EV5. Knockdown of EGFR suppressed YAP-induced cell growth in cancerous cervical cells.

A Western blot analysis showed that siRNAs of EGFR successfully knocked down EGFR in ME180 cells.

B Knockdown of EGFR blocked YAP-induced proliferation of ME180 cells. Each bar represents the mean ± SEM of five independent experimental results. Bars with different letters are significantly different from each other (CTRL-MXIV vs. siEGFR-MXIV, $P = 0.0167$; CTRL-YAP vs. siEGFR-YAP, $P < 0.0001$; CTRL-YAP^{S127A} vs. siEGFR-YAP^{S127A}, $P < 0.0001$).

C EGFR knockdown or verteporfin treatment dramatically decreased YAP-induced AREG secretion. Each bar represents the mean ± SEM of five independent experimental results. Bars with different letters are significantly different from each other (CTRL-MXIV vs. siEGFR-MXIV, $P = 0.0006$; CTRL-MXIV vs. VTPF-MXIV, $P < 0.0001$; CTRL-YAP vs. siEGFR-YAP, $P = 0.0007$; CTRL-YAP vs. VTPF-YAP, $P < 0.0001$; CTRL-YAP^{S127A} vs. siEGFR-YAP^{S127A}, $P < 0.0001$; CTRL-YAP^{S127A} vs. VTPF-YAP^{S127A}, $P < 0.0001$).

Data information: Data in (B) and (C) were analyzed for significance using two-way ANOVA in GraphPad Prism 5.

 © 2015 The Authors
