# Supplementary material for: The Hippo/YAP pathway interacts with EGFR signaling and HPV oncoproteins to regulate cervical cancer progression
Source: EMBO Mol Med. 2015 Sep 28;7(11):1426–49. doi: 10.15252/emmm.201404976 (PMC4644376; doi:10.15252/emmm.201404976)
Supplement: Supplementary file 3 [file emmm0007-1426-sd3.pdf]

The Hippo/YAP pathway interacts with EGFR signaling and HPV oncoproteins to regulate cervical cancer progression

Chunbo He, Dagan Mao, Guohua Hua, Xiangmin Lv, Xingcheng Chen, Peter C Angeletti, Jixin Dong, Steven W Remmenga, Kerry J Rodabaugh, Jin Zhou, Paul F Lambert, Peixin Yang, John S Davis, Cheng Wang

Corresponding author: Cheng Wang, University of Nebraska Medical Center

Review timeline:

Submission date:	19 December 2014
Editorial Decision:	04 February 2015
Additional Editorial Correspondence:	13 March 2015
Additional Author Correspondence :	16 March 2015
Editorial Decision:	23 March 2015
Revision received:	10 July 2015
Editorial Decision:	04 August 2015
Revision received:	18 August 2015
Accepted:	20 August 2015

Transaction Report:

(Note: With the exception of the correction of typographical or spelling errors that could be a source of ambiguity, letters and reports are not edited. The original formatting of letters and referee reports may not be reflected in this compilation.)

Editor: Roberto Buccione

1st Editorial Decision 04 February 2015

Thank you for the submission of your manuscript to EMBO Molecular Medicine. We have now heard back from the three Reviewers whom we asked to evaluate your manuscript. We are sorry that it has taken longer than we would have liked to get back to you on your manuscript. We experienced some difficulties in securing three expert and willing Reviewers and then obtaining a timely delivery of the evaluations also in connection with the submission just before the holiday season.

As you will see, while Reviewer 2 is more positive, Reviewers 1 and 3 point to significant and fundamental issues that, I am afraid, preclude publication of the manuscript in EMBO Molecular Medicine.

I will not discuss each point in detail as they are clearly stated. In brief and in aggregate and as you will see, the Reviewers 1 and 3 directly question 1) the solidity of the mechanistic foundation for the role of the Hippo pathway and also the role of HPV-E6, 2) the experimental strategies adopted and 3) the quality of the data. Reviewer 1 also notes that direct in vivo relevance of the function of YAP should be demonstrated. I should also mention that while indeed Reviewer 2 appears more positive, s/he does touch upon the issue of how supported the role of the Hippo/Yap pathway in cervical cancer is and also mentions the partially compromised novelty of the findings on clinical specimens.

Given these fundamental concerns and the overall lack of enthusiasm by the Reviewers, I have no

choice but to return the manuscript to you at this stage. In our assessment it is not realistic to expect to be able to fully address these issues experimentally and to the satisfaction of the Reviewers in a reasonable time frame.

I am sorry to have to disappoint you at this stage. I hope that the Reviewers' comments will be helpful in your continued work in this area.

***** Reviewer's comments *****

Referee #1 (Remarks):

In the present manuscript, He and colleagues identify a role for YAP, the pro-growth transcriptional co-activator protein controlled by Hippo signalling, in the progression of cervical cancer. The authors uncover an association between cervical cancer prognosis and deregulation of YAP using data from large cancer databases. Moreover, the authors identify a feed-forward signalling loop between YAP, the EGFR ligand Amphiregulin (AREG), TGF and EGFR signalling, which is reported to control cervical cancer proliferation and migration. The authors also propose that the E6-HPV protein, which is present in virtually all cervical cancers due to HPV infection, regulates YAP function by inhibiting its proteasome-dependent degradation. It is increasingly clear that YAP and Hippo signalling are crucial for the regulation of cancer progression and that they do so in coordination with other signalling pathways. As such, this manuscript is very timely. However, some of the conclusions presented in the manuscript are overstated and the supporting data is not entirely convincing, which is especially true with regards to the characterisation of the molecular mechanisms in play. If the authors address the major concerns, most notably the role of Hippo pathway signalling (and not simply YAP), the in vivo relevance of their proposed model and the molecular mechanism by which E6 regulates YAP, I believe the manuscript would be suitable for publication in EMBO Molecular Medicine.

Major Concerns

1. While the role of YAP in cervical cancer is clearly established in the manuscript, the experiments addressing the relevance of Hippo signalling fail to unequivocally support the conclusions presented by the authors. This is a crucial point the authors should address in light of recent reports that have identified YAP regulatory mechanisms that are believed to be independent of MST and LATS activity and are instead reliant on actin cytoskeleton-related signalling (Dupont et al. 2011). The data used by the authors to support their claim that Hippo signalling is involved is quite difficult to analyse. The significant protein level changes shown in Western blots are, in some instances, entirely unconvincing (Figs. 7 and 8 - in particular the decreased phosphorylation of MOB1). Moreover, there are no experiments directly addressing the role of MST or LATS (siRNA and/or overexpression) in the process. Therefore, it is possible that the Hippo pathway plays only a peripheral role in YAP regulation in this context and that alternative mechanisms are in play. Authors should extend their analyses of cell proliferation, cell migration and wound closure, and effects on YAP target genes (with and without TGF stimulation or EGFR stimulation) to siRNA or overexpression of MST or LATS kinases. If Hippo signalling is truly important, depleting or overexpressing the Hippo kinases should result in predictable changes in these parameters. In order to support their claim that the changes in protein levels are significant, Western blots should be quantified or, ideally, replaced with more convincing ones.

2. In the introduction, the sentence where Ser127 phosphorylation is related to YAP proteasomal degradation is misleading. Cytoplasmic retention of YAP and its proteasomal degradation, while both dependent on the action of LATS kinases, involve distinct phosphorylation sites (Ser127 or Ser381 and Ser384, respectively). In addition, YAP proteasomal degradation involves the action of Casein Kinase 1. This should be clearly stated in the manuscript. Also, the authors show no evidence that the regulation of YAP protein levels in cervical cancer cells is dependent on CK1 or the phosphorylation of Ser381 and Ser384. In light of their proposed mechanism of action for E6-HPV (see below), this should be addressed.

3. YAP siRNA blocks proliferation of ME-180 cells (shown in Fig. 3f). However, in this experiment cells treated with control siRNA proliferate at a much higher rate between day 4 and day 6 than in Fig. 3b, in fact reaching the level of YAP transfected cells in Fig. 3b. Does siRNA targeting YAP block proliferation of HT3 cells? Is there a similar change in the proliferation rate of cells treated with control siRNA when cells approach full confluency? What is the role of TEADs in this context?

4. Authors should provide further evidence of the in vivo relevance of YAP function in cervical cancer. For instance, in addition to overexpressing oncogenic YAP, authors should deplete YAP from cervical cancer cells and use the mouse xenograft model to analyse tumour formation. Ideally, to corroborate the role of YAP, an orthotopic cervical cancer model should be used in conjunction with a conditional YAP allele but this may be beyond the scope of the revision process. Alternatively, an in vivo evaluation of the levels of the proteins involved in YAP/EGFR signalling (i.e. AREG/TGF /EGFR) in serial sections of patient tumours would be important to support the authors' argument that a YAP/EGFR feed-forward loop is important in cervical cancer. Are AREG protein levels affected in cervical cancer? It is proposed that YAP increases AREG levels, which in turn activates YAP via EGFR but this is inferred from AREG mRNA levels.

5. The proposed model of YAP/EGFR function in cervical cancer predicts that activation of any of the main proteins would establish a feed-forward signalling loop. The model seems to imply that the feed-forward signalling loop is essential in cancer. If this is the case, why does activated YAP have a much milder phenotype than TGF treatment? Additionally, siRNA targeting YAP does not dramatically reduce the effect of TGF in cervical cancer cells, which would suggest that other pathways may be activated downstream of TGF . To what extent is EGFR signalling involved? There is no data regarding siRNA depletion of EGFR pathway proteins to complement chemical inhibition of the pathway. Conversely, does treatment with TGF , AREG or EGFR antagonists block the effects of YAP or does YAP control proliferation and migration independently of downstream EGFR activation? Finally, since the effect of YAP on EGFR signalling via AREG is thought to be non-cell autonomous, if this mechanism is essential, similar results should be obtained with conditioned medium from cells that overexpress YAP.

6. The effect of E6 on YAP stability has not been sufficiently explored and the molecular mechanism involved remains highly preliminary. Is YAP protected from degradation by E6? Evidence of E6-mediated stabilisation of YAP via the proteasome requires more support. Data from some of the experiments in this section are not very convincing (for instance, in Fig. S12, GFP-E6 has no effect on YAP levels; the effect of MG132 and CHX is clearly not as pronounced as the effect of E6 expression). Quantification of Western blot in these experiments would aid in their correct interpretation. Ideally, authors should address if E6 overexpression (in non-infected cells) or E6 siRNA (in infected cells) affects YAP ubiquitination. The in vivo results in E6/E7 infected tissue show extensive areas of HPV lesion that do not exhibit YAP upregulation and its subcellular localisation is unclear in the absence of a nuclear marker.
What is the molecular mechanism of E6-mediated YAP regulation? There is no data supporting the role of previously identified YAP regulators, i.e., TrCP (Zhao et al. 2010) or SOCS5/6 (Hong et al. 2014) and no alternative molecular mechanism is proposed. Authors should test whether TrCP or SOCS5/6 are involved in YAP regulation in this context, particularly in light of the fact that oncogenic Ras can stabilise YAP1 by downregulating SOCS5/6 expression (Hong et al. 2014). If this is the case, authors should discuss the implications of this mechanism for their YAP/EGFR feed-forward model.
The putative effect of E6 on YAP function raises interesting questions. How is YAP activated in this situation? YAP phosphorylation at Ser127 seems to be quite pronounced, which would presumably lead to its cytosolic retention and inhibition. If E6 stabilises YAP so dramatically, and considering that HPV infection rates are very prominent (~99%), why is YAP not upregulated in pre-cancerous lesions or indeed the prevalence of cervical cancer not higher than 1%?

7. Data presented in Table 1 and Fig. 1b is confusing. In both cases YAP positivity (as defined by the authors) from 69 tumour samples is being represented. However, in Table 1 the percentage of each class of tumours positive for YAP expression (weak, moderate or strong) is being represented (amounting to 97.1% of tumours), while in Fig. 1b the actual value of positivity is being represented (approximately 35% across all tumours).

8. Readouts presented in several figures should be quantified to determine their significance:
a) YAP nuclear localisation in tissue samples;
b) Phosphorylation levels of YAP (Fig. 3a and 3c; Fig. S9)
c) Ki67 staining (Fig. 5g)

9. The upregulation of YAP target genes in ME180 cells (WT, YAP and YAP S127A) shown in Fig. S7 is not entirely convincing (particularly AREG and EGFR). Is there a concomitant increase in protein levels? Are these effects dependent on TEADs? This could be tested using YAP-TEAD interaction inhibitors or by depleting the relevant TEADs.

Minor Concerns

1. Do the cervical cancer cell lines used throughout the manuscript have mutations in YAP?

2. Is there a role for TGF in this process? TGF -related proteins feature heavily in Fig. S2 where YAP linker genes are analysed in cervical cancer and the pathway is known to influence Hippo signalling (Varelas et.al, Dev Cell 2010).

3. It is proposed that TGF /EGFR signalling is involved in cervical cancer cell proliferation and migration, but is there a role for Ras? It has recently been reported that YAP1 compensates for loss of oncogenic Ras in certain contexts (Zhang et al. 2014; Shao et al. 2014; Kapoor et al. 2014). Oncogenic Ras is thought to promote YAP stabilisation via the regulation of SOCS proteins (Hong et al. 2014). Is this mechanism compatible with the proposed action of E6 on YAP in cervical cancer?

4. The authors state that the transfection efficiency of YAP plasmids is high on the basis of increased expression of YAP in the selected cell lines (Fig. 3e). However, the fact that endogenous YAP is expressed in these cells masks the transfection efficiency. Transfection efficiency would be more readily assessed by quantifying the percentage of cells expressing a co-transfected marker gene (i.e GFP); if this has not been done, authors should remove the reference to transfection efficiency.

5. Page 8 should read "TEADs are the major mediators of YAP transcriptional activities". In the following sentence Supplementary Fig. S2 is wrongly referenced and the subsequent sentence should be rephrased: "Moreover, network analysis shows that almost all genes interacted with YAP,...".

6. Page 9 should read "and reduced the proportion of cells in G1 phase in both ME180 and HT3..."

7. Page 10 should read "which may be attributed to multi-layered cell growth". Moreover, Fig. 3g is incorrectly referenced as Fig. 2g.

8. Page 17 Fig. 9g is incorrectly references as Fig. 8g.

9. Supplementary Fig. S1 and S9 denotes Ect1 cells as End1 cells. Is this a mistake or are they indeed different cell lines to Ect1 cells?

10. Panels in Figure 1 should be organised differently. If 1g and 1h are magnified images of 1d and 1f, this should be indicated in 1d and 1f. If not, organising panels alphabetically would be advisable as, as it is, the data can be confusing.

Referee #2 (Remarks):

The manuscript by He et al. describes the functional analysis of the Hippo signaling pathway in HPV-induced cervical carcinogenesis.
YAPp overexpression in cervical carcinomas is presented as well as YAP function in cervical cancer

cell lines in relation to EGFR and TGFa signalling.
This is a clearly written manuscript with an impressing amount of in silico and functional data, which are well presented.
To date very little knowledge exists on the Hippo signaling pathway in cervical cancer, which underlines the novelty of the data presented. Moreover, the findings on clinical specimens presented herein are supported by recently published data by Xiao et al. (Expression of Yes-associated protein in cervical squamous epithelium lesions. Xiao H, Wu L, Zheng H, Li N, Wan H, Liang G, Zhao Y, Liang J.Int J Gynecol Cancer. 2014 Nov;24(9):1575-82. doi: 10.1097/IGC.0000000000000259). This paper most likely came out after submission and needs to be incorporated in the revision.
The data presented certainly contribute to our understanding of cervical cancer development, but whether it plays a central role, as suggested in the title is a matter of debate. I would suggest to omit the word central or to rephrase the title.

Specific comments:

-Figure 1 shows immunohistochemical staining of tissue micro arrays and demonstrates an increased expression in cervical cancers. As the two cervical cancer histotypes (squamous cell carcinomas and adenocarcinomas)often display different expression patterns, it needs to be indicated how the staining patterns relate to tumor histotype. Moreover, as the functional data show a role of E6 in Hippo signalling, the regulation YAP overexpression may be related to HPV presence rather than or in addition to tumor stage. To this end it is worthwhile to know whether the normal samples were HPV-positive or negative and to learn about YAP staining in cervical cancer precursor lesions (CIN lesions). As described by Xiao et al, increased YAP expression may represent an early event also detectable in HPV-positive CIN lesions. Additionally, it is unclear whether nuclear staining was taken into account when scoring the micro arrays (as was described for Figure 11).

- I have some fundamental problems with the E6E7 immortalized endocervical and ectorcervical cell line being referred to a normal cervical cells (Fig S1 and further). First of all they express the viral oncogenes in dividing cells, a characteristic of high-grade CIN lesions and cancer cells and not normal cells. Moreover, they are most likely genetically instable and telomerase positive. I would suggest to refer to them as HPV-immortalized cells, rather than normal cells. It would be of interest, but probably undoable to include primary cervical cells in the analysis.

-Figure 4: are the differences in colony formation statistically significant?

-Data are presented on HPV-positive (ME-180) and HPV-negative cell lines (HT-3), showing similar expression levels and functional effects (Fig 1-4 and ME-180 vs suppl figures). Therefore it is questionable whether there is a specific role for E6 in YAP activation in the ME-180 cell line. For the E6 knock-down experiments shown in Figure 9 HeLa cells were used. Why not ME-180 cells? Related to this it would be interesting to know what mechanism drives YAP activation in the HT-3 cells (see Figure 12b)?

-The effect of E6 on YAP phosphorylation is studied in HT-3 cells, but not E7. To strengthen a specific role of E6, E7 function needs to be studied as well as well as a combination of both.

-The stainings shown in Figure 11 are not very clear.

Referee #3 (Comments on Novelty/Model System):

The experiments are poorly designed and poorly controlled.

Referee #3 (Remarks):

In this manuscript, He and colleagues investigate the YAP pathway in a variety of cervical cancer cell lines. They propose that the pathway is important in the progression of cervical cancer. Although this pathway may indeed play an important role in the progression of cervical cancer (and many other cancers), the study mainly defines phenomena in certain cell lines that are difficult to

extend to other cell lines and/or to cervical cancer in general. The experiments that attempt to implicate HPV E6 in the maintenance of high YAP levels are poorly conceived and controlled, making the results difficult to interpret. Overall, the use of cell lines more typically employed in Hippo pathway studies, perhaps compared to some of these cervical cancer cell lines, would have made the study more useful.

Comments:

The Hippo pathway has been well defined in several cell types, including MCF10A cells. Has the pathway ever been examined in any of the cell types used here? Have any of the findings presented here been tested in MCF10A or other well-established cell models, either by this group or other researchers?

Figure 1: The results section corresponding to this figure should begin with a description of the experiment, rationale, and source of tissue.

Table 2: This is very difficult to interpret as presented.

Figure S1 and throughout the work: End1 cells are endocervical cells that have been immortalized via the introduction of HPVE6 and E7. These are a poor choice as a negative control for experiments examining HPV-associated cancers, as they express the transforming proteins of HPV16. Later in the study, the authors highlight the observation that E6 induces an increased level of YAP protein - if this is the case, an E6-negative control should be included in this experiment and in all other experiments. Additionally, these Western blots and nearly all the Western blots included in the manuscript are overexposed. It is difficult to appreciate many of the differences in protein level suggested in the text.

Figure S2 is also difficult to understand. What is the definition of a YAP linker gene?

Figures 3-4 and Figures S3-S6: The authors observe that YAP overexpression drives increased cellular proliferation and migration. Indeed, this is a consequence of YAP overexpression that has been appreciated since YAP was first identified as an oncogene (Overholtzer et al, PNAS 2006). The observation that YAP overexpression drives increased proliferation of the cell lines characterized here is not surprising.

Figures 6-7, S7-S10. The link proposed by the authors between YAP signaling and TGFalpha expression many be very indirect. They extend this to perform a series of experiments in which various cancer cells are treated with TGFalpha and in general, are more proliferative after TGFalpha treatment. This does not seem to be a surprising result nor does it add much to an understanding of YAP and/or TGFalpha signaling. Do the authors mean to imply that this is the case only in cervical cancer cells? Technically, many of these blots are too overexposed to interpret. Also, siGLO is not an appropriate non-targeting control in siRNA experiments, as it is not incorporated into or processed by the RISC complex. It is useful only as a transfection control.

Figure 9A-E, Figure 10: Here, the authors use an unorthodox method to express HPV16 E6 in cells - they treat the cells with recombinant E6 for as little as 1 hr. Presumably, they attribute the effects they observe to the intracellular functions of E6. What is the evidence that E6 is internalized into the target cells and that it is functional once there? Many more controls are needed and a different system of E6 expression would be preferable. There is also no attempt to examine the effects of HPV E7 nor is there a reason given for the focus on E6.

Figure 9F-H: For how long were the cells treated with siRNAs? The 6h treatments described for other experiments in the Materials and Methods may be much too short to have any meaningful effect on protein levels.

Additional Editorial Correspondence 13 March 2015

I am sorry for the delay in getting back to you on your request to reconsider our post-review decision on your manuscript. Firstly, as a matter of natural priority, new submissions must take precedence over appeals, and we have been recently receiving many new submissions which only recently allowed me to take on your case; secondly, I wished to consult with an expert advisor on your manuscript but I have been unable to find a willing advisor with the necessary expertise over the last few days.

Although I will still keep working on this and am confident that over the next week a decision can be made, I have further discussed your appeal with a colleague and we came to the conclusion, in any case, it needs to be reformulated.

Please prepare as quickly as possible a new rebuttal where you simply and clearly state what you are prepared to do and/or provide concerning each point raised by each Reviewer and stating your case where you do not agree. If you feel that a Reviewer comment is too general for you to offer a direct action, please say so clearly and as a matter of fact! Please send me this re-formulated rebuttal letter by return email.

This would work much better for your case and eventually, would allow us to discuss internally if I do not succeed in gaining further advice in a reasonable time frame.

Again, I apologise that it is taking a little but I hope you appreciate that we are trying to accommodate your request as fairly as possible.

Appeal 16 March 2015

Thank you very much for re-considering our manuscript. Attached is the re-formulated rebuttal letter. I believe that our point-by-point response will make you and reviewers give us an opportunity to revise our manuscript.

The cervical cancer patient deep-sequencing datasets in TCGA database have been updated recently and the deep-sequencing cancer patient samples have increased from 36 to 191 cases. These deep-sequencing data derived from cervical cancer patients (191 cases) clearly indicated that the Hippo pathway is de-regulated and YAP oncogene is amplified and activated (please see the figure in the new rebuttal letter for detail). The perfect match between these multi-dimensional genomic data and our novel findings, as well as the strong clinical relevance of our study, clearly indicate that our manuscript is suitable to be published in the EMBO Molecular Medicine.

Thanks again for your kind help.

Reviewer #1

Reviewer #1 general comments: *In the present manuscript, He and colleagues identify a role for YAP, the pro-growth transcriptional co-activator protein controlled by Hippo signalling, in the progression of cervical cancer. The authors uncover an association between cervical cancer prognosis and deregulation of YAP using data from large cancer databases. Moreover, the authors identify a feed-forward signalling loop between YAP, the EGFR ligand Amphiregulin (AREG), TGFα and EGFR signalling, which is reported to control cervical cancer proliferation and migration. The authors also propose that the E6-HPV protein, which is present in virtually all cervical cancers due to HPV infection, regulates YAP function by inhibiting its proteasome-dependent degradation. It is increasingly clear that YAP and Hippo signalling are crucial for the regulation of cancer progression and that they do so in coordination with*

other signalling pathways. As such, this manuscript is very timely. However, some of the conclusions presented in the manuscript are overstated and the supporting data is not entirely convincing, which is especially true with regards to the characterisation of the molecular mechanisms in play. If the authors address the major concerns, most notably the role of Hippo pathway signalling (and not simply YAP), the in vivo relevance of their proposed model and the molecular mechanism by which E6 regulates YAP, I believe the manuscript would be suitable for publication in EMBO Molecular Medicine.

Authors' response: We thank reviewer #1 for his comments that the manuscript are "timely" and "If the authors address the major concerns, most notably the role of Hippo pathway signalling (and not simply YAP), the in vivo relevance of their proposed model and the molecular mechanism by which E6 regulates YAP, I believe the manuscript would be suitable for publication in EMBO Molecular Medicine". We also appreciate his constructive comments on how to improve our work. Below is the point-to-point response to reviewer's concerns.

Reviewer's comments: *While the role of YAP in cervical cancer is clearly established in the manuscript, the experiments addressing the relevance of Hippo signalling fail to unequivocally support the conclusions presented by the authors. This is a crucial point the authors should address in light of recent reports that have identified YAP regulatory mechanisms that are believed to be independent of MST and LATS activity and are instead reliant on actin cytoskeleton-related signalling (Dupont et al. 2011). The data used by the authors to support their claim that Hippo signalling is involved is quite difficult to analyse. The significant protein level changes shown in Western blots are, in some instances, entirely unconvincing (Figs. 7 and 8 - in particular the decreased phosphorylation of MOB1). Moreover, there are no experiments directly addressing the role of MST or LATS (siRNA and/or overexpression) in the process. Therefore, it is possible that the Hippo pathway plays only a peripheral role in YAP regulation in this context and that alternative mechanisms are in play. Authors should extend their analyses of cell proliferation, cell migration and wound closure, and effects on YAP target genes (with and without TGFα stimulation or EGFR stimulation) to siRNA or overexpression of MST or LATS kinases. If Hippo signalling is truly important, depleting or overexpressing the Hippo kinases should result in predictable changes in these parameters. In order to support their claim that the changes in protein levels are significant, Western blots should be quantified or, ideally, replaced with more convincing ones.*

Authors' response: We thank reviewer for the constructive suggestion. We conclude that the Hippo pathway plays role in the progression of cervical cancer because of phosphorylation of LATS1/2, MOB1 and dephosphorylation of YAP in response to TGFα, which in turn is regulated by YAP expression levels. Moreover, the deep-sequencing data from TCGA dataset clearly indicated that the Up-stream genes of the Hippo/YAP signaling are frequently down-regulated, while the YAP and the downstream genes are frequently up-regulated in cervical cancer cells. However, our

data do not exclude the possibility of the existence of the Hippo pathway-independent activation of YAP gene. Therefore, following reviewer's suggestion, we will use shRNA to knock down MST1/2 and LATS1/2 from these cell lines and then determine the phosphorylation status of the major kinases in the Hippo/YAP pathway and cell proliferation, differentiation and cell migration. As suggested, we will also quantify all the western bands and make sure that results are presented clearly.

As mentioned by reviewer, the role of YAP in cervical cancer is clearly established in the manuscript. If we find that the Hippo component MST and

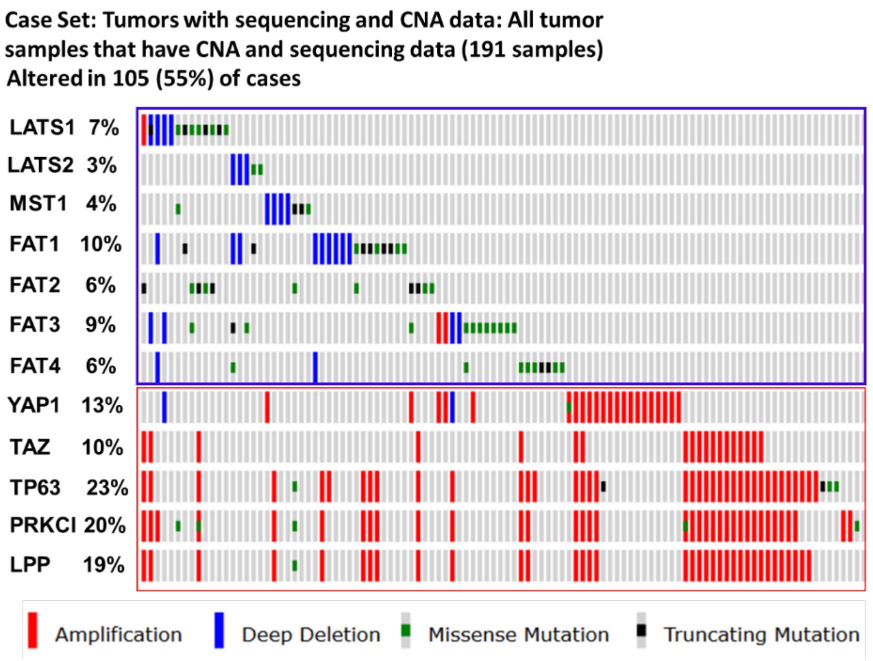

LATS do not plays a major role, we will modify the title and conclusion to reflect the role of YAP oncogene in the progression of cervical cancer.
Reviewer's comments: In the introduction, the sentence where Ser127 phosphorylation is related to YAP proteasomal degradation is misleading. Cytoplasmic retention of YAP and its proteasomal degradation, while both dependent on the action of LATS kinases, involve distinct phosphorylation sites (Ser127 or Ser381 and Ser384, respectively). In addition, YAP proteasomal degradation involves the action of Casein Kinase 1. This should be clearly stated in the manuscript. Also, the authors show no evidence that the regulation of YAP protein levels in cervical cancer cells is dependent on CK1 or the phosphorylation of Ser381 and Ser384. In light of their proposed mechanism of action for E6-HPV (see below), this should be addressed.
Authors' response: we will modify the introduction of the manuscript to reflect these facts. Moreover, we will perform experiments to determine whether YAP is phosphorylated on serine 381. We will also use CK1 siRNA or CK1 inhibitor (such as D4476 and IC261) to determine the involvement of CK1 in E6-mediated stabilization of YAP. We believe that acute treatment with these selective inhibitors of CK1 will prove more useful than knockdown of CK1 with siRNA, which is anticipated to have detrimental effects on the cells.

Reviewer's comments: *YAP siRNA blocks proliferation of ME-180 cells (shown in Fig. 3h). However, in this experiment cells treated with control siRNA proliferate at a much higher rate between day 4 and day 6 than in Fig. 3b, in fact reaching the level of YAP transfected cells in Fig. 3b. Does siRNA targeting YAP block proliferation of HT3 cells? Is there a similar change in the proliferation rate of cells treated with control siRNA when cells approach full confluency? What is the role of TEADs in this context?*
Authors' response: First, we notice that YAP regulation of cervical cancer cell proliferation in a cell density-dependent manner. In fig 3h, cells reached a higher density, cells with normal YAP (siGLO group) still grow, while cells with YAP-knockdown stop growing. Similarly, in figure 3b, before cell reach confluence, the growth rates of the control and YAP-overexpressing cells are almost the same. However, when cell reached confluence, YAP-overexpressing cells keep growing, while the growth rate of the control cells drops. Moreover, cells in fig. 3h experienced cell transfection procedure (with different culture condition), while cells in the fig 3b were established stable cell lines with MXIV vectors. Therefore, the growth rates of these two groups of cells are not directly comparable. The experiments were also performed at different times during the preparation of the manuscript. To address this issue, we will repeat these experiments with both wild type (+/- siRNA) and ME-MXIV cells under identical conditions to allow more direct comparisons.
HT-3 cells are not infected by HPV. YAP level in this cell line is lower than that in ME180 cells. That may explain why HT-3 cells grow slower than ME-180 cells. To answer reviewer's question, we will knock down YAP in HT3 cells to see it effect on cell proliferation. We believe that the similar results will be observed.
Since verteporfin is an antagonist of YAP and TEAD interaction, we will use verteporfin to verify the role of TEAD in mediating YAP action in cervical cancer cells.
Reviewer's comments: Authors should provide further evidence of the in vivo relevance of YAP function in cervical cancer. For instance, in addition to overexpressing oncogenic YAP, authors should deplete YAP from cervical cancer cells and use the mouse xenograft model to analyse tumour formation. Ideally, to corroborate the role of YAP, an orthotopic cervical cancer model should be used in conjunction with a conditional YAP allele but this may be beyond the scope of the revision process.
Alternatively, an in vivo evaluation of the levels of the proteins involved in YAP/EGFR signalling (i.e. AREG/TGFα/EGFR) in serial sections of patient tumours would be important to support the authors' argument that a YAP/EGFR feed-forward loop is important in cervical cancer. Are AREG protein levels affected in cervical cancer? It is proposed that YAP increases AREG levels, which in turn activates YAP via EGFR but this is inferred from AREG mRNA levels.
Authors' response: As mentioned by the reviewer "the *role of YAP in cervical cancer is clearly established in the manuscript"* We agree with the review that these experiments are useful but we feel that we have already established a role for YAP. Actually, we are developing cervical tissue-specific YAP-overexpression mouse using KRT14-CRE mice, ROSA26-rtTA

mice and tetO-YAP mice in our laboratory. As indicated by the reviewer, conducting extra xenograft experiments with stable YAP knockdown cell lines (need several months) and creating conditional YAP knockout animal models (need one to two years) are beyond the scope of the revision process. We will detect the expression of AREG and EGFR proteins in the serial patient sections using IHC. These results will be reported in the future manuscripts.

Reviewer's comments: The proposed model of YAP/EGFR function in cervical cancer predicts that activation of any of the main proteins would establish a feed-forward signalling loop. The model seems to imply that the feed-forward signalling loop is essential in cancer. If this is the case, why does activated YAP have a much milder phenotype than TGFα treatment? Additionally, siRNA targeting YAP does not dramatically reduce the effect of TGFα in cervical cancer cells, which would suggest that other pathways may be activated downstream of TGFα. To what extent is EGFR signalling involved? There is no data regarding siRNA depletion of EGFR pathway proteins to complement chemical inhibition of the pathway. Conversely, does treatment with TGFα, AREG or EGFR antagonists block the effects of YAP or does YAP control proliferation and migration independently of downstream EGFR activation? Finally, since the effect of YAP on EGFR signalling via AREG is thought to be non-cell autonomous, if this mechanism is essential, similar results should be obtained with conditioned medium from cells that overexpress YAP.
Authors' response: Due to the complex downstream signaling network of EGFR, we believe that TGFa may regulate cervical cancer cell proliferation via multiple signaling pathways. Our present finding, together with a previous report that HPV16-E6 protein is able to stabilize EGFR protein in the cervical cancer cells, clearly indicate that the EGFR-Hippo feed-forward loop is an important mechanism that drives the cervical cancer progression. Following the reviewer's suggestion, we will use EGFR siRNA to knock down EGFR to complement chemical inhibition of the pathway. We will also use EGFR antagonists to further confirm the functionality of these feed-forward loop. We will also use conditioned medium from the ME180-YAP^{S127A} cells to examine the existence and the functionality of this loop.

Reviewer's comments: The effect of E6 on YAP stability has not been sufficiently explored and the molecular mechanism involved remains highly preliminary. Is YAP protected from degradation by E6? Evidence of E6-mediated stabilisation of YAP via the proteasome requires more support. Data from some of the experiments in this section are not very convincing (for instance, in Fig. S12, GFP-E6 has no effect on YAP levels; the effect of MG132 and CHX is clearly not as pronounced as the effect of E6 expression). Quantification of Western blot in these experiments would aid in their correct interpretation. Ideally, authors should address if E6 overexpression (in non-infected cells) or E6 siRNA (in infected cells) affects YAP ubiquitination. The in vivo results in E6/E7 infected tissue show extensive areas of HPV lesion that do not exhibit YAP upregulation and its subcellular localisation is unclear in the absence of a nuclear marker. What is the molecular mechanism of E6-mediated YAP regulation? There is

no data supporting the role of previously identified YAP regulators, i.e., βTrCP (Zhao et al. 2010) or SOCS5/6 (Hong et al. 2014) and no alternative molecular mechanism is proposed. Authors should test whether βTrCP or SOCS5/6 are involved in YAP regulation in this context, particularly in light of the fact that oncogenic Ras can stabilise YAP1 by downregulating SOCS5/6 expression (Hong et al. 2014). If this is the case, authors should discuss the implications of this mechanism for their YAP/EGFR feed-forward model.
The putative effect of E6 on YAP function raises interesting questions. How is YAP activated in this situation? YAP phosphorylation at Ser127 seems to be quite pronounced, which would presumably lead to its cytosolic retention and inhibition. If E6 stabilises YAP so dramatically, and considering that HPV infection rates are very prominent (~99%), why is YAP not upregulated in pre-cancerous lesions or indeed the prevalence of cervical cancer not higher than 1%?
Authors' response:
1. In fig S12, the effect of E6 on YAP1 expression is not so pronounced because of the low transfection efficiency of E6-expressing vector. We will use newly established E6-overexpressing vectors to transfect HT3 cells in the revised manuscript.
2. We will also quantify data in Fig 10 as requested by reviewer.
3. We will provide better images to present the expression level and localization of YAP in the transgenic mouse tissues.
4. In the present study, we found that the Hippo/YAP pathway plays a role in the initiation and progression of cervical cancer using both *in vivo* and *in vitro* models. We also found that YAP is required for HPV E6 induced cervical cancer cell growth. Currently, we are focusing on uncovering the molecular mechanism of E6-mediated YAP protein stabilization. One of our ongoing projects in the laboratory is focusing on the E6-regulated ubiquitination and deubiquitination of the key kinases of the Hippo/YAP pathway. Our preliminary studies indicate that that E6 potentially interacts with other kinases in the Hippo/YAP signaling pathway to regulate YAP protein levels. Moreover, it seems that E3 ubiquitin ligases other than βTrCP and SOCS5/6 are involved in this process. Except for the in vitro studies with cell lines, we are also trying to develop endogenous animal models to figure out the molecular mechanisms underlying HPV oncoprotein regulation of YAP protein level and activity. We believe that large amount of interesting and important data will be produced from this project and these data will be critical for us to more completely understand the molecular events associated HPV E6-mediated YAP oncoprotein regulation. We will include some additional data in the revised version of our manuscript as described in the response to reviewer 1.However, detailed in depth experimental approaches will require considerable time and are likely to form the basis of a separate manuscript.
To the question that "If E6 stabilises YAP so dramatically, and considering that HPV infection rates are very prominent (~99%), why is YAP not upregulated in pre-cancerous lesions or indeed the prevalence of cervical cancer not higher than 1%?", we have to say that we are also puzzled by this phenomenon. Similarly, although high risk HPVs have been identified as causative reagents by many laboratory, clinical, and epidemiological

studies, the same question still exists for high risk HPV virus. However, we feel that our findings support the idea that targeting the Hippo/YAP pathway will provide a promising therapeutic strategy for the treatment of cervical cancer. We also believe that results from our present study significantly deepen our understanding on the molecular mechanisms underlying the initiation and progression of cervical cancer.

Reviewer's comments: *Data presented in Table 1 and Fig. 1b is confusing. In both cases YAP positivity (as defined by the authors) from 69 tumour samples is being represented. However, in Table 1 the percentage of each class of tumours positive for YAP expression (weak, moderate or strong) is being represented (amounting to 97.1% of tumours), while in Fig. 1b the actual value of positivity is being represented (approximately 35% across all tumours).*

Authors' response: We are sorry for the confusing data presentation. Table 1 presents YAP immunosignal intensity in normal and tumor tissues. The Immunosignal intensity was classified into negative, weak, medium and strong according to the YAP staining positivity in normal and cancerous tissues.

Reviewer's comments: Readouts presented in several figures should be quantified to determine their significance:

a) YAP nuclear localisation in tissue samples;
b) Phosphorylation levels of YAP (Fig. 3a and 3c; Fig. S9)
c) Ki67 staining (Fig. 5g)

Authors' response: we will provide the quantification according to reviewer's suggestion.

Reviewer's comments: The upregulation of YAP target genes in ME180 cells (WT, YAP and YAP S127A) shown in Fig. S7 is not entirely convincing (particularly AREG and EGFR). Is there a concomitant increase in protein levels? Are these effects dependent on TEADs? This could be tested using YAP-TEAD interaction inhibitors or by depleting the relevant TEADs.

Authors' response: We will provide quantification of our data. We will also perform analysis of protein levels with western blot or ELISA (only for TGFa and AREG if antibody is not good for western blot). As mentioned earlier, we will use vertiporfin (antagonist for YAP and TEADs interaction) to determine the role of TEADs in regulating EGFR, TGFA, and AREG.

Reviewer's minor concern #1. Do the cervical cancer cell lines used throughout the manuscript have mutations in YAP?

Authors' response: Five cell lines are in use in this study. YAP protein levels in each cell are different. YAP gene mutation in these cells are unknown. However, after analyzing the TCGA deep-sequencing datasets, we found that 1 out of 391 cervical cancer patients (~0.26%) carry mutated YAP, suggesting that YAP mutation does not play important role in cervical cancer. Moreover, our observations show that all cell lines used in this study respond to TGFα and AREG stimulation (with a dephosphorylation of YAP at serine 127), suggesting that YAP in these cell lines is not mutated, or mutation status of YAP do not affect the function of this oncogene in these cells.

Reviewer's minor concern #2 Is there a role for TGFβ in this process? TGFβ-related proteins feature heavily in Fig. S2 where YAP linker genes

© EMBO

are analysed in cervical cancer and the pathway is known to influence Hippo signalling (Varelas et.al, Dev Cell 2010).
Authors' response: We did not check the function of TGFβ in these cells. However, a previous report has shown the interaction between TGFβ signaling and the Hippo/YAP signaling. Moreover, we found that TGFβ signaling pathway interacts with the Hippo/YAP signaling pathways in ovarian cancer cells. This will be an area of future investigation.

Reviewer's minor concern #3. It is proposed that TGFα/EGFR signalling is involved in cervical cancer cell proliferation and migration, but is there a role for Ras? It has recently been reported that YAP1 compensates for loss of oncogenic Ras in certain contexts (Zhang et al. 2014; Shao et al. 2014; Kapoor et al. 2014). Oncogenic Ras is thought to promote YAP stabilisation via the regulation of SOCS proteins (Hong et al. 2014). Is this mechanism compatible with the proposed action of E6 on YAP in cervical cancer?
Authors' response: Since our manuscript was under consideration by EMBO J, several reports has shown interaction between Ras and YAP. As an important part of the EGFR signaling, Ras might be involved in this process. We will present this argument in our discussion.

Reviewer's minor concern #4 The authors state that the transfection efficiency of YAP plasmids is high on the basis of increased expression of YAP in the selected cell lines (Fig. 3e). However, the fact that endogenous YAP is expressed in these cells masks the transfection efficiency. Transfection efficiency would be more readily assessed by quantifying the percentage of cells expressing a co-transfected marker gene (i.e GFP); if this has not been done, authors should remove the reference to transfection efficiency.
Authors' response: We did not use GFP to determine the transfection efficiency since our cells are stably transfected. These cell lines were selected with G418. The transfection efficiency should be very high because cells without vector will be eliminated by the culture system.
Reviewer's minor concern #5. Page 8 should read "TEADs are the major mediators of YAP transcriptional activities". In the following sentence Supplementary Fig. S2 is wrongly referenced and the subsequent sentence should be rephrased: "Moreover, network analysis shows that almost all genes interacted with YAP,..".
Authors' response: Thanks, this will be corrected in the revised version.

Reviewer's minor concern #6. Page 9 should read "and reduced the proportion of cells in G1 phase in both ME180 and HT3..."
Authors' response: Thanks, this will be corrected in the revised version.

Reviewer's minor concern #7. Page 10 should read "which may be attributed to multi-layered cell growth". Moreover, Fig. 3g is incorrectly referenced as Fig. 2g.
Authors' response: Thanks, this will be corrected in the revised version.

Reviewer's minor concern #8. Page 17 Fig. 9g is incorrectly references as

Fig. 8g.
Authors' response: Thanks, this will be corrected in the revised version.

Reviewer's minor concern #9. Supplementary Fig. S1 and S9 denotes Ect1 cells as End1 cells. Is this a mistake or are they indeed different cell lines to Ect1 cells?
Authors' response: Thanks, this will be corrected in the revised version.

Reviewer's minor concern #10. Panels in Figure 1 should be organised differently. If 1g and 1h are magnified images of 1d and 1f, this should be indicated in 1d and 1f. If not, organising panels alphabetically would be advisable as, as it is, the data can be confusing.
Authors' response: Thanks, this will be corrected in the revised version.

Reviewer #2:

Reviewer's general comments: The manuscript by He et al. describes the functional analysis of the Hippo signaling pathway in HPV-induced cervical carcinogenesis. YAP overexpression in cervical carcinomas is presented as well as YAP function in cervical cancer cell lines in relation to EGFR and TGFa signalling.
This is a clearly written manuscript with an impressing amount of in silico and functional data, which are well presented. To date very little knowledge exists on the Hippo signaling pathway in cervical cancer, which underlines the novelty of the data presented. Moreover, the findings on clinical specimens presented herein are supported by recently published data by Xiao et al. (Expression of Yes-associated protein in cervical squamous epithelium lesions. Xiao H, Wu L, Zheng H, Li N, Wan H, Liang G, Zhao Y, Liang J.Int J Gynecol Cancer. 2014 Nov;24(9):1575-82. doi: 10.1097/IGC.0000000000000259). This paper most likely came out after submission and needs to be incorporated in the revision. The data presented certainly contribute to our understanding of cervical cancer development, but whether it plays a central role, as suggested in the title is a matter of debate. I would suggest to omit the word central or to rephrase the title.

Authors' response: We appreciate reviewer #2 for his positive comments. We are happy that a recent paper published by Xiao *et al.* supports our work. We will cite this paper in the revised manuscript. We will also revise our title and conclusion and remove the word "central".
Reviewer's specific comments: -Figure 1 shows immunohistochemical staining of tissue micro arrays and demonstrates an increased expression in cervical cancers. As the two cervical cancer histotypes (squamous cell carcinomas and adenocarcinomas) often display different expression patterns, it needs to be indicated how the staining patterns relate to tumor histotype. Moreover, as the functional data show a role of E6 in Hippo signalling, the regulation YAP overexpression may be related to HPV presence rather than or in addition to tumor stage. To this end it is worthwhile to know whether the normal samples were HPV-positive or

negative and to learn about YAP staining in cervical cancer precursor lesions (CIN lesions). As described by Xiao et al, increased YAP expression may represent an early event also detectable in HPV-positive CIN lesions. Additionally, it is unclear whether nuclear staining was taken into account when scoring the micro arrays (as was described for Figure 11).
Author's response: The samples used in this study are commercial microarray. In keeping with the observations that around 80% of cervical cancers are squamous cell carcinoma, the tissue microarray contains close to 90% squamous cell carcinomas and the remainder are adenocarcinoma samples. We will provide an analysis of YAP expression and perform statistical analysis using the immunosignal positivity and intensity data derived from each tumor histotype. The stated histotypes will be validated by a Pathologist associated with the project.
According to our finding, we agree with reviewer that "the regulation YAP overexpression may be related to HPV presence rather than or in addition to tumor stage". Although we cannot directly confirm this inference with our own samples (because no HPV infection information was recorded in the information page of these microarrays), we are really happy to see that this was confirmed by Xiao and colleagues recently (Int J Gynecol Cancer. 2014). We will cite this paper in the revised manuscript. Our staining results showed that in the cervical tumor cells, YAP was mainly localized to the nucleus regardless of stage or grade.
Reviewer's specific comments: I have some fundamental problems with the E6E7 immortalized endocervical and ectorcervical cell line being referred to a normal cervical cells (Fig S1 and further). First of all they express the viral oncogenes in dividing cells, a characteristic of high-grade CIN lesions and cancer cells and not normal cells. Moreover, they are most likely genetically instable and telomerase positive. I would suggest to refer to them as HPV-immortalized cells, rather than normal cells. It would be of interest, but probably undoable to include primary cervical cells in the analysis.
Author's response: We appreciate this suggestion. We will refer these cells as HPV-immortalized cells in the revised manuscript.
Reviewer's specific comments: Figure 4: are the differences in colony formation statistically significant?
Author's response: Yes, we will clearly mark this in the revised manuscript.
Reviewer's specific comments: Data are presented on HPV-positive (ME-180) and HPV-negative cell lines (HT-3), showing similar expression levels and functional effects (Fig 1-4 and ME-180 vs suppl figures). Therefore it is questionable whether there is a specific role for E6 in YAP activation in the ME-180 cell line. For the E6 knock-down experiments shown in Figure 9 HeLa cells were used. Why not ME-180 cells? Related to this it would be interesting to know what mechanism drives YAP activation in the HT-3 cells (see Figure 12b)?
Author's response: The characteristics of HT3 and ME180 cell lines actually support our conclusion. ME180 cells (HPV positive) grow faster than HT3 cells (HPV negative). Actually, ME180 cells grows so fast that we have to use serum-reduced medium (1%~2.5% FBS in medium) to culture these cells. HT3 cells grow slowly even in the regular growth medium (with 10% FBS). Moreover, The HPV positive ME180 cells have higher YAP levels

compared to the HPV-free HT3 cells. Furthermore, ME180 cells form tumors faster than HT3 cells in the athymic nude mice. The tumor xenografts derived from ME180 cells also grow more rapidly than that of HT3 cells.
We used HeLa cells in the E6 knock-down experiments because this cell line is derived from invasive cervical cancer cells and are HPV E6 positive. This is now stated in the manuscript. Both YAP and E6 protein levels are higher in this cell line, making it an ideal cellular model to study HPV E6 and YAP interaction using gene knockdown technique. Importantly, our findings have been replicated with multiple cell lines, which serves to help us to make reasonable conclusions. Finally, use of multiple cell lines in a study is encouraged and sometimes required by many scientific Journals. To address reviewer's concern, we will repeat this experiment with ME180 cells and present data in the supplementary information of the revised manuscript.

Reviewer's specific comments: The effect of E6 on YAP phosphorylation is studied in HT-3 cells, but not E7. To strengthen a specific role of E6, E7 function needs to be studied as well as well as a combination of both.
Author's response: We also believe that inclusion of HPV16 E7, or other HPV early gene products such as E2 and E5 proteins, may produce very interesting data. Reagents other than E6 are not readily available. However, we also believe that expanding the current study to include HPV16 E7 and other early genes will significantly expand the manuscript and dilute the already large amount of research presented in this manuscript. Please note that we have already presented 12 busy figures and two tables in the manuscript. We have also presented 12 figures in the supplementary information section. After completing the experiments that are necessary for us to answer the questions raised by reviewers, the revised manuscript will have more than 40 figures and two tables. Inclusion of even one more factor (such as E7) means that we have to use this new factors to repeat all experiments that we have done with E6 in this manuscript. Moreover, we need to assess combined effects. However, we agree with reviewer that future studies using HPV16 E7, or other HPV early gene products such as E2 and E5 proteins, may produce very interesting data.
Reviewer's specific comments: The stainings shown in Figure 11 are not very clear.
Author's response: We will provide better images to present the expression level and localization of YAP in the transgenic mouse tissues.

Reviewer #3:

Reviewer's comments: *Although this pathway may indeed play an important role in the progression of cervical cancer (and many other cancers), the study mainly defines phenomena in certain cell lines that are difficult to extend to other cell lines and/or to cervical cancer in general.*
Authors' response: In this study we employed (1) five established cervical cancer cell lines; (2) TCGA data sets (with convincing deep sequencing

data) that clearly show that across all tested cancers (88 studies), cervical cancer has the highest level of alterations in YAP, and (3) an established transgenic mouse model of cervical cancer to indicate the potential clinical relevance. In summary, we feel that this study is highly relevant to cervical cancer.

Reviewer's comments: *The experiments that attempt to implicate HPV E6 in the maintenance of high YAP levels are poorly conceived and controlled, making the results difficult to interpret".*

Authors' response: We appreciate reviewer #3 for his time. However, we do not agree with the reviewer for the following reasons:

1) The observation that HPV16 E6 stabilizes YAP was an unexpected and novel finding. Before designing our experiments, we analyzed the protein structure of HPV and Hippo pathway components. Based on our analysis, we did not expect any direct interaction between the YAP and HPV16 E6 proteins, and accordingly, did not put any efforts towards this direction. However, in experiments designed to test whether E6 treatment would alter expression of YAP we observed that treatment of HPV-negative cervical cancer cells (HT-3 cells) with E6 led to an increase in YAP protein but not YAP mRNA. This interesting finding directed us to explore the potential mechanisms behind the observed discrepancy in YAP protein and mRNA levels. As mentioned in response to reviewer #1, we will provide additional data to uncover the molecular mechanism underlying E6 stabilization of YAP protein in cervical cancer cells.

2) Our experiments are well-controlled. We first used MG132, the proteasome inhibitor, to inhibit the proteasome activity. Inhibition of proteasome led to accumulation of YAP and EGFR protein in HT-3 cells (HPV-free cervical cancer cells) within 4 hours, suggesting the involvement of proteasome in YAP protein degradation. We then used cycloheximine (CHX) to inhibit protein synthesis. We found that both YAP and EGFR protein decreased within 4 hours in cells treated with CHX. However, if HPV16 E6 was added to the culture, YAP protein level did not decrease in cells treated with CHX. These results clearly indicated that treatment with HPV16 E6 protein prevents the YAP protein from protease-induced protein degradation. This is a standard design for the protein degradation analysis (see examples: Genes & Dev. 2010. 24: 72-85; *Molecular cell.* 2008, 29: 350–361).

3) Our manuscript has been read by several well-established scientists in this field, including Dr. John S Davis (professor, UNMC), Dr. Peter Angeletti (associated professor, university of Nebraska-Licoin), Dr. Paul Lambert (university of Wisconsin –Madison), Dr, Kerry Rodabaugh (associated professor, UNMC), Dr. Sobudha Lele (professor, UNMC) and Dr. Steve Remenga (professor, UNMC). Everyone believes that we have very interesting and important findings that deserves publication in a decent Journal.

The general comments " this work is *poorly conceived and controlled,*" Do not provide useful information for us to identify the specific weaknesses of our manuscript and thereby the opportunity for us to improve our manuscript.

Reviewer's comments: *Overall, the use of cell lines more typically employed in Hippo pathway studies, perhaps compared to some of these cervical cancer cell lines, would have made the study more useful.*
Authors' response: *W*e think that this suggestion is ambiguous. First, the present study is focused on cervical cancer. HPV causes more than 99% of the cervical squamous cell carcinomas. Therefore, it is appropriate to use cervical cancer cells. The Hippo pathway has been proven to play critical roles in many different types of organ and tissues. One pioneering study in this field was conducted by our co-author, Dr. Jixin Dong. He used drosophila and liver cells to investigate the role of YAP in organ size control and tumorigenesis (Dong et al. *cell*, 2007, 130: 1120-1133). However, we do not believe that drosophila and liver hepatocytes have clinical relevance to cervical cancer and HPV infection. Our studies used HT-3 cervical cell line (HPV negative) and ME-180 cervical cells (HPV positive), which we feel are relevant for cervical cancer research.
Reviewer's comments: *The Hippo pathway has been well defined in several cell types, including MCF10A cells. Has the pathway ever been examined in any of the cell types used here? Have any of the findings presented here been tested in MCF10A or other well-established cell models, either by this group or other researchers?*
Authors' response: in the introduction, we indicated that the Hippo/YAP signaling pathway has not been investigated in cervical cancer. Therefore, the study provides novel information about this important pathway in cervical cancer using five immortalized and cancerous cervical epithelial cell lines. In the present studies, our main focus was the role of the Hippo/YAP pathway in cervical cancer and the potential interaction YAP and HPV, the major causative agents of cervical cancer. We do not believe that using MCF10A (an immortalized human mammary gland epithelial cell line) is a useful model for cervical cancer. Importantly, a previous study suggested that YAP may function as a tumor suppressor in certain types of mammary cancer (Yuan et al, 2008).
Reviewer's comments: *Figures 3-4 and Figures S3-S6: The authors observe that YAP overexpression drives increased cellular proliferation and migration. Indeed, this is a consequence of YAP overexpression that has been appreciated since YAP was first identified as an oncogene (Overholtzer et al, PNAS 2006). The observation that YAP overexpression drives increased proliferation of the cell lines characterized here is not surprising.*
Authors' response: As stated above, to date there is nothing known about the role of YAP in cervical cancer. YAP functions as an oncogene in some tissues and a tumor suppressor in others. Therefore, identifying components of the Hippo/YAP signaling pathway and provide novel information on its regulation and function in cervical cancer is new and makes an important contribution to the field. Importantly, we our focus was not just that YAP promotes cervical cancer cell proliferation; we provide new insight on a previously unprecedented mechanism underlying cervical cancer initiation and progression.
Reviewer's comments: *Figures 6-7, S7-S10. The link proposed by the authors between YAP signaling and TGFalpha expression many be very indirect. They extend this to perform a series of experiments in which*

various cancer cells are treated with TGFalpha and in general, are more proliferative after TGFalpha treatment. This does not seem to be a surprising result nor does it add much to an understanding of YAP and/or TGFalpha signaling. Do the authors mean to imply that this is the case only in cervical cancer cells?
Authors' response: Our study indicated that expression of YAP or constitutively active YAP in cervical cancer cells lead to significant increase in expression of EGFR and its two ligands, TGFα and AREG. Indeed, we found that treatment of cervical cancer cells with TGFα and AREG stimulated proliferation and migration. Importantly, we also report in our study that the stimulatory actions of TGFα on the cervical cancer cells was dependent on the presence of YAP. This evidence, together with the observation that TGFα and AREG, via activating EGFR, suppressed the Hippo pathway and activated YAP protein, clearly indicate that the EGFR signaling pathway and the Hippo/YAP signaling pathway interact to form an autocrine loop that promotes cervical cancer cell proliferation. As mentioned above, we will perform additional experiments to further establish the proposed feed-forward loop.
The finding that HPV E6 stabilizes the YAP protein connects this feedback loop with the cervical cancer causative agent and strengthens the clinical relevance of our finding. In the context of the whole study, we conducted a logical series of experiments and have provided mechanistic data showing how the Hippo/YAP pathway contributes to cervical cancer cell proliferation.
Reviewer's comments: *Figure 1: The results section corresponding to this figure should begin with a description of the experiment, rationale, and source of tissue.*
Authors' response: Although this information was presented in the MATERIALS AND METHODS section we will add additional description in the Results section as requested.
Reviewer's comments: *Figure S1 and throughout the work: End1 cells are endocervical cells that have been immortalized via the introduction of HPVE6 and E7. These are a poor choice as a negative control for experiments examining HPV-associated cancers, as they express the transforming proteins of HPV16. Later in the study, the authors highlight the observation that E6 induces an increased level of YAP protein - if this is the case, an E6-negative control should be included in this experiment and in all other experiments.*
Authors' response: End1 cells are immortalized, "non-tumorigenic" cervical epithelial cells that express HVP proteins. We used the End1 cells in select experiments. We observed that overexpression of YAP induced colony formation in these cells. This experiment successfully verified our observations that YAP is a oncogene in the cervical epithelial cells. As shown in our study we also employed HT3, the only available HPV negative cervical cancer cell line. We used these cells to show that HPV16 E6 increased the YAP protein. We will define the cell types more clearly in the results section to clarify the rationale for the experiments.
Reviewer's comments: *Figure 9A-E, Figure 10: Here, the authors use an unorthodox method to express HPV16 E6 in cells - they treat the cells with recombinant E6 for as little as 1 hr. Presumably, they attribute the effects they observe to the intracellular functions of E6. What is the evidence that*

E6 is internalized into the target cells and that it is functional once there? Many more controls are needed and a different system of E6 expression would be preferable. There is also no attempt to examine the effects of HPV E7 nor is there a reason given for the focus on E6.

Authors' response: Recombinant HPV16 E6 protein has been frequently used in cervical cancer research (*The Journal of Immunology,* 2001, 167: 497–504; Cancer research, 1999, 59: 1184–1187). These recombinant HPV16 E6 and E7 proteins are translocated to the nucleus within minutes (*Journal of Virology*, 2003, 77: 2330–2337). In this study, we treated cells with recombinant HPV16 E6 protein for multiple time points (1h, 8h, 24h, 48h and 96h). We found that E6 was able to affect the protein level of YAP as early as 1h. Therefore, we used 1h time point to determine if HPV16 E6 acutely stimulates YAP gene transcription (mRNA). We also used 8h and 48h to determine the effect of E6 on YAP mRNA expression (Fig 10). For YAP protein expression, we treated HT3 cells with HPV16 E6 for 48h. Moreover, we confirmed our findings by knocking down E6 protein with E6 siRNA in HeLa cells.

We selected E6 protein because the recombinant E6 protein has been frequently used in cervical cancer research and it is the only commercially available HPV early gene product. That is why we focused on HPV E6 protein in this study. We believe that future studies using HPV16 E7, or other HPV early gene products such as E2 and E5 proteins, may produce very interesting data.

Reviewer's comments: *Figure 9F-H: For how long were the cells treated with siRNAs? The 6h treatments described for other experiments in the Materials and Methods may be much too short to have any meaningful effect on protein levels.*

Authors' response: Sorry for any confusion; the siRNA transfection procedure typically takes about 6h (depending on the siRNA used). The cells are allowed a 48h recovery time before treatment. The treatment time of recombinant HPV16 E6 is 48h or 72h after siRNA transfection. However, our data showed that 8h treatment of HT-3 cells with recombinant HPV16 E6 can affect the protein levels of YAP (see Fig 10c-10f).

Reviewer's comments: *Figures 6-7, S7-S10. Also, siGLO is not an appropriate non-targeting control in siRNA experiments, as it is not incorporated into or processed by the RISC complex. It is useful only as a transfection control.*

Authors' response: When we first perform knockdown experiments, we usually use five controls to optimize our transfection efficiency. These controls include: the non-transfected cells, the siGLO RISC-free negative control group, the lipofectene2000 group, the target siRNA only group, and lipofectene + target siRNA group. These pre-transfection tests have shown that siGLO negative control has no effect on gene expression, cell viability and cell proliferation (as reported by the manufacture. http://dharmacon.gelifesciences.com/sirna/siglo-risc-free-control/). The advantage of using siGLO RISC-free negative control is that it serves not only as a non-targeting siRNA control, but also a transfection efficiency marker. siGLO non-targeting siRNA makes it possible for us to monitor transfection efficiencies between experiment groups and necessary

repeats. We will add a new non-targeting siRNA as a control in the revised version.

2nd Editorial Decision 23 March 2015

Thank you for your request to reconsider our post-review reject decision and your March 16, 2015 rebuttal letter.

After discussion, and considered the points you make and the further experimentation you plan to undertake, we would be happy to re-evaluate a revised manuscript, with the understanding that your revision will undergo a further round of review and that acceptance or rejection of the manuscript will depend on the completeness of your responses included in the next, final version of the manuscript and as outlined in your appeal rebuttal letter.

As you know, EMBO Molecular Medicine has a "scooping protection" policy, whereby similar findings that are published by others during review or revision are not a criterion for rejection. I do ask you however, to get in touch with us after three months if you have not completed your revision, to update us on the status. Please also contact us as soon as possible if similar work is published elsewhere.

EMBO Molecular Medicine now requires a complete author checklist (http://embomolmed.embopress.org/authorguide#editorial3) to be submitted with all revised manuscripts. Provision of the author checklist is mandatory at revision stage; The checklist is designed to enhance and standardize reporting of key information in research papers and to support reanalysis and repetition of experiments by the community. The list covers key information for figure panels and captions and focuses on statistics, the reporting of reagents, animal models and human subject-derived data, as well as guidance to optimise data accessibility. Please make sure that the relevant information is also included in the main manuscript text.

I look forward to receiving your revised manuscript in due time.

1st Revision - authors' response 10 July 2015

We appreciate the reviewer's positive and constructive comments about our manuscript. We also appreciate the issues raised by reviewers to improve our manuscript. We have addressed the reviewer's concerns point by point and edited the manuscript very carefully (changes in the manuscript are marked in red). We believe that these changes have met the reviewer's concerns and have improved the impact of our paper.

Reviewer #1

Reviewer #1 general comments: *In the present manuscript, He and colleagues identify a role for YAP, the pro-growth transcriptional co-activator protein controlled by Hippo signalling, in the progression of cervical cancer. The authors uncover an association between cervical cancer prognosis and deregulation of YAP using data from large cancer databases. Moreover, the authors identify a feed-forward signalling loop between YAP, the EGFR ligand Amphiregulin (AREG), TGFα and EGFR signalling, which is reported to control cervical cancer proliferation and*

migration. The authors also propose that the E6-HPV protein, which is present in virtually all cervical cancers due to HPV infection, regulates YAP function by inhibiting its proteasome-dependent degradation. It is increasingly clear that YAP and Hippo signalling are crucial for the regulation of cancer progression and that they do so in coordination with other signalling pathways. As such, this manuscript is very timely. However, some of the conclusions presented in the manuscript are overstated and the supporting data is not entirely convincing, which is especially true with regards to the characterisation of the molecular mechanisms in play. If the authors address the major concerns, most notably the role of Hippo pathway signalling (and not simply YAP), the in vivo relevance of their proposed model and the molecular mechanism by which E6 regulates YAP, I believe the manuscript would be suitable for publication in EMBO Molecular Medicine.

Authors' response: We thank reviewer for his/her comments that the manuscript are "timely" and "If the authors address the major concerns, most notably the role of Hippo pathway signalling (and not simply YAP), the *in vivo* relevance of their proposed model and the molecular mechanism by which E6 regulates YAP, I believe the manuscript would be suitable for publication in *EMBO Molecular Medicine*". We also appreciate his constructive comments on how to improve our work. Below is the point-to-point response to reviewer's concerns.

Reviewer's comments: *While the role of YAP in cervical cancer is clearly established in the manuscript, the experiments addressing the relevance of Hippo signalling fail to unequivocally support the conclusions presented by the authors. This is a crucial point the authors should address in light of recent reports that have identified YAP regulatory mechanisms that are believed to be independent of MST and LATS activity and are instead reliant on actin cytoskeleton-related signalling (Dupont et al. 2011). The data used by the authors to support their claim that Hippo signalling is involved is quite difficult to analyse. The significant protein level changes shown in Western blots are, in some instances, entirely unconvincing (Figs. 7 and 8 - in particular the decreased phosphorylation of MOB1). Moreover, there are no experiments directly addressing the role of MST or LATS (siRNA and/or overexpression) in the process. Therefore, it is possible that the Hippo pathway plays only a peripheral role in YAP regulation in this context and that alternative mechanisms are in play. Authors should extend their analyses of cell proliferation, cell migration and wound closure, and effects on YAP target genes (with and without TGFα stimulation or EGFR stimulation) to siRNA or overexpression of MST or LATS kinases. If Hippo signalling is truly important, depleting or overexpressing the Hippo kinases should result in predictable changes in these parameters. In order to support their claim that the changes in protein levels are significant, Western blots should be quantified or, ideally, replaced with more convincing ones.*

Authors' response: We thank reviewer for the very constructive suggestion. We conclude that the Hippo pathway plays role in the progression of cervical cancer because of decreased phosphorylation of LATS1/2, MOB1 and dephosphorylation of YAP in response to TGFα and AREG, which are in turn regulated by YAP. Although these data imply the involvement of the

Hippo pathway in the process, we agree with the reviewer that these data do not exclude the possibility of the existence of the Hippo pathway-independent activation of YAP gene. To provide the direct evidence for the involvement of the Hippo pathway, following the reviewer's suggestion, we knocked down LATS1/2 in ME180 cells with LATS1/2 siRNA. We found that knockdown of LATS1/2 suppressed YAP phosphorylation, enhanced the anchorage-independent growth, promoted cell proliferation both in 2D and 3D cultured systems and increased AREG secretion in cervical cancer cells. These data are presented in Fig 8a, Fig 8b, and supplementary figure S17. As suggested, we also replaced the Western blot bands and quantified all the western results to make sure that our results are solid. Most importantly, we analyzed deep-sequencing data from 191 cervical cancer patients, which was extracted from the TCGA database to confirm the clinical relevance of this study. The results from this multidimensional cancer genomic data clearly indicate that the genes coding for the up-stream regulators of the Hippo/YAP signaling pathway are frequently down-regulated (mutation and deletion), while YAP and its downstream target genes are frequently up-regulated in cervical cancer cells (Supplementary Fig. S2). The sequencing data from 191 patients not only supports the involvement of the Hippo pathway in cervical carcinogenesis, but also provides strong evidence for the clinical relevance of the present study.

Reviewer's comments: *In the introduction, the sentence where Ser127 phosphorylation is related to YAP proteasomal degradation is misleading. Cytoplasmic retention of YAP and its proteasomal degradation, while both dependent on the action of LATS kinases, involve distinct phosphorylation sites (Ser127 or Ser381 and Ser384, respectively). In addition, YAP proteasomal degradation involves the action of Casein Kinase 1. This should be clearly stated in the manuscript. Also, the authors show no evidence that the regulation of YAP protein levels in cervical cancer cells is dependent on CK1 or the phosphorylation of Ser381 and Ser384. In light of their proposed mechanism of action for E6-HPV (see below), this should be addressed.*

Authors' response: We appreciate the constructive suggestion. We have modified the introduction of the manuscript to reflect the fact that distinct phosphorylation sites are involved in YAP cytoplasmic retention and protease-dependent degradation. Moreover, we performed experiments to examine the relationship between phosphorylation sites and the degradation of YAP in the cervical cancer cells. Our results indicated that YAP was also dephosphorylated at Ser397 (Ser381 in YAP protein isoform 2) by both TGFα (Figure 6b) and AREG treatment (Fig. 7a). This suggests that activation of EGFR may also affect YAP degradation. Surprisingly, we also found that expression of HPV16E6 in HT-3 cells greatly increased YAP and CK1ε expression (Fig. 10f). This indicates that in cervical cancer cells, the Hippo pathway-induced, CK1-mediated βTrCP-SCF ubiquitin ligase complex may not be the key pathway that induces YAP protein degradation, although Casein Kinase 1 has been reported to be critical for proteasome-dependent YAP degradation. Interestingly, we found that ubiquitin ligase complex substrate recognition factor SOCS6 may be involved in the stabilizing YAP in cervical cancer cells (Fig. 10f, 10g, and 10h). However, many more experiments are required to uncover the factor(s) underlying this critical biological event. The fact that we do not

understand exactly how YAP stability is regulated does not detract from the important and novel findings presented in this study.

Reviewer's comments: *YAP siRNA blocks proliferation of ME-180 cells (shown in Fig. 3h). However, in this experiment cells treated with control siRNA proliferate at a much higher rate between day 4 and day 6 than in Fig. 3b, in fact reaching the level of YAP transfected cells in Fig. 3b. Does siRNA targeting YAP block proliferation of HT3 cells? Is there a similar change in the proliferation rate of cells treated with control siRNA when cells approach full confluency? What is the role of TEADs in this context?*

Authors' response: The cells in original Fig. 3b were cultured with medium containing relatively lower concentrations of serum (2.5% FBS, because the ME180 cells grow very fast). Cells in the original Fig. 3h were cultured with regular serum (10%). Therefore, the growth rates of these two groups of cells are not directly comparable. Second, we find that YAP regulates cervical cancer cell proliferation in a cell density-dependent manner. In original Fig 3h, cells reached a higher density, cells with normal YAP (siCtrl group) still grow, while cells with YAP-knockdown stop growing. Similarly, in original figure 3b, before cells reach confluence, the growth rates of the control and YAP-overexpressing cells are almost the same. However, when cells reached confluence, YAP-overexpressing cells kept growing, while the growth rate of the control cells decreased.

To address this issue, we repeated these experiments (2.5% FBS was also used for knockdown cells) and obtained the similar results (Fig. 2 & Fig. S7). Since FBS is able to induce the activation of YAP, we examined the proliferation of all these cell lines under serum-reduced conditions (1% FBS). We found the growth rate of the ME180-YAP^{S127A} cells was significantly higher than that of the ME180-YAP cells, while growth rate of the ME180-YAP cells was significant higher than that of ME180-MXIV cells, even before the cell reach confluence (Fig. S4). These result suggested that YAP not only plays a role in overcoming cell contact inhibition, but also can promote cell proliferation under the conditions of nutritional deficiency.

HT3 cells are not infected by HPV. YAP levels in this cell line are lower than that in ME180 cells. Our previous data (Fig. 9) showed that depleting YAP in HT3 cells not only suppressed cell proliferation, but also blocked HPV16 E6 induced cell growth. To answer reviewer's question "Is there a similar change in the proliferation rate of cells treated with control siRNA when cells approach full confluence?" We knocked down YAP in HT3 cells and observed results similar to those which were observed in ME180 cells (Fig. 2h).

Since verteporfin (VTPF) is an antagonist of YAP and TEAD interaction (Liu-Chittenden et al, 2012), we used verteporfin to verify the role of TEAD in mediating YAP action in cervical cancer cells. We found VTPF not only blocked basal and YAP-induced AREG production in ME180 cells (Supplementary Fig. S18), but also inhibited basal and YAP-stimulated anchorage-independent growth of ME180 cells (Fig. 8d). In addition, VTPT disrupt cell-cell communication and suppressed cell proliferation in the 3D culture system (Fig.8d). These results indicate that TEADs-YAP complex is required for the regulation of the growth of cervical cancer cells.

Reviewer's comments: *Authors should provide further evidence of the in vivo relevance of YAP function in cervical cancer. For instance, in addition to overexpressing oncogenic YAP, authors should deplete YAP from cervical cancer cells and use the mouse xenograft model to analyse tumour formation. Ideally, to corroborate the role of YAP, an orthotopic cervical cancer model should be used in conjunction with a conditional YAP allele but this may be beyond the scope of the revision process.*

Alternatively, an in vivo evaluation of the levels of the proteins involved in YAP/EGFR signalling (i.e. AREG/TGFα/EGFR) in serial sections of patient tumours would be important to support the authors' argument that a YAP/EGFR feed-forward loop is important in cervical cancer. Are AREG protein levels affected in cervical cancer? It is proposed that YAP increases AREG levels, which in turn activates YAP via EGFR but this is inferred from AREG mRNA levels.

Authors' response: As mentioned by the reviewer "the *role of YAP in cervical cancer is clearly established in the manuscript"* We agree with the reviewer that these experiments are useful but we feel that we have already established a role for YAP. Actually, we are developing cervical tissue-specific YAP-overexpression mouse using KRT14-CRE mice, ROSA26-rtTA mice and tetO-YAP mice in our laboratory. As indicated by the reviewer, creating conditional YAP knockout/knock-in animal models (needing one to two years) are beyond the scope of the revision process. We thank reviewer for understanding that *in vivo* mouse models are time-consuming (may take years some time) and it is not feasible to put all data in one manuscript.

Following the reviewer's suggestion, we conducted xenograft experiments with stable YAP knockdown (Lentivirus-based YAP shRNA) cell lines. We found that knockdown of YAP almost eliminated the ability of ME180 cancer cell to form tumors *in vivo* (Fig 4h, 4i &4j), providing further evidence that the YAP protein plays an important role in cervical cancer progression.

Although EGFR as a predictor of response to therapy is controversial, the up-regulation of EGFR expression has been well defined in cervical cancer (T Soonthornthum et al, 2011). We found that the expression of EGF-like ligands such as AREG and TGFα, as well as EGFR were up-regulated by YAP in cervical cancer cells (Figure 5a). Analysis of RNA-seq data extracted from the TCGA multidimensional cancer genomic database clearly indicate that YAP mRNA expression is significantly correlated with EGFR in cervical cancer patients (supplementary Fig. S11).

To further confirm this conclusion, we used immunofluorescence to detect the expression of EGFR in in human cervical tumor xenografts (derived from ME180-MXIV, ME180-YAP & ME180-YAP^{S127A}) in the athymic nude mice. As expected, the EGFR immunosignal in tumors derived from ME180-YAP and ME180-YAP^{S127A} cells were dramatically stronger than that in ME180-MXIV tumor tissues (see below images).

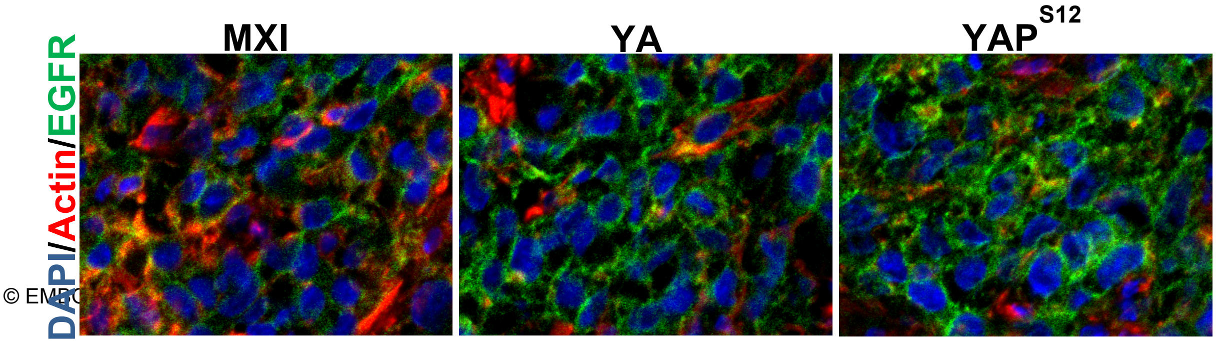

© EM

Finally, we tried to use several AREG antibodies to detect the expression of AREG in patient sections by using IHC, but the sensitivity and specificity of these AREG antibodies was a significant concern. To further confirm the involvement of the AREG in YAP-induced cervical cancer growth, we used an ELISA kit to detect the secretion of AREG in cervical cancer cell conditioned medium. Results showed that overexpression of YAP increased AREG secretion, while knockdown of YAP inhibited AREG secretion (Supplementary Fig. S10b & S10c). Most importantly, knockdown of LATS1/2 significantly induced the production of AREG, while knockdown of EGFR or HPV18E6, or blocking YAP-TEAD interaction using verteporfin, suppressed AREG production (Fig. 9g, 9i, Supplementary Fig. S17d, S17e & S18c).

Therefore, we believe that our results from cellular models, xenograft mouse models and human patient samples (TCGA sequencing data) convincingly suggest the existence of *YAP/EGFR feed-forward loop*.

Reviewer's comments: *The proposed model of YAP/EGFR function in cervical cancer predicts that activation of any of the main proteins would establish a feed-forward signalling loop. The model seems to imply that the feed-forward signalling loop is essential in cancer. If this is the case, why does activated YAP have a much milder phenotype than TGFα treatment? Additionally, siRNA targeting YAP does not dramatically reduce the effect of TGFα in cervical cancer cells, which would suggest that other pathways may be activated downstream of TGFα. To what extent is EGFR signalling involved? There is no data regarding siRNA depletion of EGFR pathway proteins to complement chemical inhibition of the pathway. Conversely, does treatment with TGFα, AREG or EGFR antagonists block the effects of YAP or does YAP control proliferation and migration independently of downstream EGFR activation? Finally, since the effect of YAP on EGFR signalling via AREG is thought to be non-cell autonomous, if this mechanism is essential, similar results should be obtained with conditioned medium from cells that overexpress YAP.*

Authors' response: Due to the complex downstream signaling network of EGFR, we believe that TGFα may regulate cervical cancer cell proliferation via multiple signaling pathways. Our present finding, together with a previous report that HPV16-E6 protein is able to activate EGFR protein in the cervical cancer cells, clearly indicate that the EGFR-Hippo feed-forward loop is an important mechanism that drives the cervical cancer progression.

Following the reviewer's suggestion, we used EGFR siRNA to knock down EGFR to complement chemical inhibition of the pathway. We found that knockdown of EGFR blocked YAP-induced cells proliferation and AREG secretion in ME180 cells (Supplementary Fig. S18b &S18c). We also used EGFR inhibitor (AG1478) to further confirm the functionality of this feed-forward loop. Results showed that AG1478 successfully blocked YAP-enhanced anchorage-independent growth of cervical cancer cells (Fig. 8d). Finally, we used AREG ELISA kit to detect the production of AREG in conditioned medium and results were descripted in last response (Fig. 9i, Supplementary Fig. S10b, S10c S17d, S17e &S18c). Combining with RNA sequencing data from TCGA (Fig. S11), we believed that the Hippo/YAP-EGFR/Ligands loop is present in cervical cancer cells and it may be critical for cervical cancer cell growth.

Reviewer's comments: *The effect of E6 on YAP stability has not been sufficiently explored and the molecular mechanism involved remains highly preliminary. Is YAP protected from degradation by E6? Evidence of E6-mediated stabilisation of YAP via the proteasome requires more support. Data from some of the experiments in this section are not very convincing (for instance, in Fig. S12, GFP-E6 has no effect on YAP levels; the effect of MG132 and CHX is clearly not as pronounced as the effect of E6 expression). Quantification of Western blot in these experiments would aid in their correct interpretation. Ideally, authors should address if E6 overexpression (in non-infected cells) or E6 siRNA (in infected cells) affects YAP ubiquitination. The in vivo results in E6/E7 infected tissue show extensive areas of HPV lesion that do not exhibit YAP upregulation and its subcellular localisation is unclear in the absence of a nuclear marker.*
What is the molecular mechanism of E6-mediated YAP regulation? There is no data supporting the role of previously identified YAP regulators, i.e., βTrCP (Zhao et al. 2010) or SOCS5/6 (Hong et al. 2014) and no alternative molecular mechanism is proposed. Authors should test whether βTrCP or SOCS5/6 are involved in YAP regulation in this context, particularly in light of the fact that oncogenic Ras can stabilise YAP1 by downregulating SOCS5/6 expression (Hong et al. 2014). If this is the case, authors should discuss the implications of this mechanism for their YAP/EGFR feed-forward model.
The putative effect of E6 on YAP function raises interesting questions. How is YAP activated in this situation? YAP phosphorylation at Ser127 seems to be quite pronounced, which would presumably lead to its cytosolic retention and inhibition. If E6 stabilises YAP so dramatically, and considering that HPV infection rates are very prominent (~99%), why is YAP not upregulated in pre-cancerous lesions or indeed the prevalence of cervical cancer not higher than 1%?

Authors' response:

1) In fig S12, the effect of E6 on YAP1 expression is not so pronounced because of the low transfection efficiency of E6-expressing vector. So we used a new Lentivirus based HPV16E6-overexpressing vectors to transfect HT3 cells and established the HT3-E6 cell line in the revised manuscript. We found E6 expression in HT3 cells dramatically increased YAP protein level (Fig. 10f).

2) We provided new images to exhibit the expression level and localization of YAP in the K14-WT, K14-HPV16E6 and K14-HPV16E6/E7 transgenic mouse tissues (Fig. 11a-11f).

3) In the present study, we found that the Hippo/YAP pathway plays a role in the initiation and progression of cervical cancer using both *in vivo* and *in vitro* models. We also found that YAP is required for HPV E6 induced cervical cancer cell growth. Currently, we are focusing on uncovering the molecular mechanism of E6-mediated YAP protein stabilization. One of our ongoing projects in the laboratory is focusing on the E6-regulated ubiquitination and deubiquitination of the key kinases of the Hippo/YAP pathway. In addition to the *in vitro* studies with cell lines, we are also trying to develop endogenous animal models to figure out the molecular mechanisms underlying HPV oncoprotein regulation of YAP protein and activity. We believe that large amount of interesting and important data will be produced from this project and these data will be critical for us to more

completely understand the molecular events associated HPV E6-mediated YAP oncoprotein regulation. We also believe that the present manuscript in its revised form provides a sufficient body of work worthy of publication.

Following the reviewer's suggestion, we checked the effect of HPV E6 expression on LAST1/2, βTrCP, SOCS6 and CK1 protein levels. Our data indicated that E6 potentially interacts with SOCS6 rather than β-TrCP and CK1 to regulate YAP protein levels (Fig. 10f, 10g &10h). As mentioned by the reviewer, SOCS6 also was involved in Ras-mediated YAP stability (Hong et al. 2014). However, our very recent preliminary studies indicated that in cervical cancer, except for the ubiquitin ligase, deubiquitinases are also involved in cervical cancer progression and HPV16 E6-mediated YAP protein stabilization. We believe that the molecular mechanisms underlying HPV E6-mediated YAP stabilization are much more complex and many more experiments are required to understand this important and intriguing biological event in cervical cancer initiation and progression.

In our experiments we noticed that HPV E6 increased both total YAP and phosphorylation YAP at Ser127, but decreased phosphorylation YAP at Ser397 (at Ser318 in YAP isoform 2). As mentioned by the reviewer, previous studies showed that phosphorylation YAP at Ser127 would lead to its cytosolic retention and inhibition. But it is still unknown that whether the phosphor-YAP (S127) has any additional functional roles in the cytoplasm. A recent study showed that the middle T antigen of polyomavirus (PyMT) physically interacts with TAZ and YAP to promote cell transformation (Shanzer M et al, 2014). The authors showed that YAP and TAZ were required for cell transformation by PyMT, but counter-intuitively, both Yap and TAZ are exclusively cytoplasmic in the presence of PyMT. The authors showed the physical interaction between YAP/TAZ and PyMT, but these interactions are dispensable for PyMT-induced TAZ and YAP cytoplasmic localization. The authors demonstrated that a LATS refractory TAZ/YAP mutant did not undergo cytoplasmic retention induced by PyMT. These findings describe a noncanonical activation of LATS, and an unprecedented TEADs-independent role for TAZ and YAP in viral-mediated oncogenesis, suggesting that the phospho-YAP (S127) may have some unknown function to regulate cell growth. Combined with our findings, we believe that the phospho-YAP (S127) may have a dual function: 1) directly regulate cell growth via an unknown mechanism; 2) maintain a reservoir of YAP protein in cells for emergent situations.

To further confirm our findings, we used human foreskin keratinocytes (HFKs) raft cultures, which is a core experimental model for HPV investigation, to test the interaction between HPV16 and YAP. As expected, compared to normal HFKs rafts, HPV16 plasmid-containing raft cultures were apparently hyper-proliferative (Fig. 11g & 11h). Moreover, we found that the signal intensity of YAP in HPV16 raft cultures was much higher than that in the normal HFKs raft cultures (Fig. 11g, 11h, 11i & 11j). More importantly, the staining results revealed intense YAP signals in a high percentage of mid-to upper spinous cells (Fig. 11h). This abrupt increase in YAP signal is identical to both the pattern consistently observed in naturally occurring HPV lesions and distribution of HPV DNA in HFKs raft cultures (Stoler & Broker, 1986; Wang et al, 2009). We also noticed that HPV16 increased YAP signals both in the nucleus and cytoplasm in this culture system. Results from this

model system further confirm our observation that the interaction between YAP and HPV are very important for HPV induction of cervical epithelial neoplasia.

To the question that "If E6 stabilizes YAP so dramatically, and considering that HPV infection rates are very prominent (~99%), why is YAP not upregulated in pre-cancerous lesions or indeed the prevalence of cervical cancer not higher than 1%?". We have to say that we are also puzzled by this phenomenon. Similarly, although high risk HPVs have been identified as causative reagents by many laboratory, clinical, and epidemiological studies, the same question still exists for high risk HPV virus. Moreover, under physiological conditions, more factors should be considered, rather than the results observed in our simplified model. For example, the role of the immune system (due to viral infection) in the interaction between the Hippo pathway and HPV oncoprotein should be clarified before we answer that question. However, we feel that our findings support the idea that targeting the Hippo/YAP pathway will provide a promising therapeutic strategy for the treatment of cervical cancer. We also believe that results from our present study will attract more scientist in different research field to uncover the molecular mechanisms underlying the initiation and progression of cervical cancer.

Reviewer's comments: *Data presented in Table 1 and Fig. 1b is confusing. In both cases YAP positivity (as defined by the authors) from 69 tumour samples is being represented. However, in Table 1 the percentage of each class of tumours positive for YAP expression (weak, moderate or strong) is being represented (amounting to 97.1% of tumours), while in Fig. 1b the actual value of positivity is being represented (approximately 35% across all tumours).*

Authors' response: We are sorry for the confusing data presentation. Table 1 presents YAP immunosignal intensity in normal and tumor tissues. The Immunosignal intensity was classified into negative, weak, medium and strong according to the YAP staining positivity in normal and cancerous tissues.

Reviewer's comments: *Readouts presented in several figures should be quantified to determine their significance:*

a) YAP nuclear localisation in tissue samples;

b) Phosphorylation levels of YAP (Fig. 3a and 3c; Fig. S9)

c) Ki67 staining (Fig. 5g)

Authors' response: In the revised version, we provide the quantitative data for Phosphorylation levels of YAP (Fig. 3a and 3c; Fig. S9) and Ki67 staining (Fig. 5g). For the IHC quantification, we used software (Aperio ImageScope) to analyze the YAP the staining signal to avoid potential subjective errors. However, this software can not to distinguish the nucleus and cytoplasm.

Reviewer's comments: *The up-regulation of YAP target genes in ME180 cells (WT, YAP and YAP S127A) shown in Fig. S7 is not entirely convincing (particularly AREG and EGFR). Is there a concomitant increase in protein levels? Are these effects dependent on TEADs? This could be tested using YAP-TEAD interaction inhibitors or by depleting the relevant TEADs.*

Authors' response: We provided quantitative data (Fig. 5a) for the original Fig. S7 (Fig. S10a in the revised version). We also analyzed protein levels with western blot (for EGFR, Fig. S10b) or

ELISA (for AREG, Fig. S10b & S10c). As mentioned earlier, we also used vertiporfin (antagonist for YAP and TEADs interaction) to determine the role of TEADs in regulating AREG.

Reviewer's minor concern #1*. Do the cervical cancer cell lines used throughout the manuscript have mutations in YAP?*

Authors' response: Five cell lines are in use in this study. YAP protein levels in each cell are different. YAP gene mutations in these cells are unknown. However, after analyzing the TCGA deep-sequencing datasets, we found that 1 out of 391 cervical cancer patients (~0.26%) carry mutated YAP, suggesting that YAP mutation may not play an important role in cervical cancer. Moreover, our observations show that all cell lines used in this study respond to TGFα and AREG stimulation (with a dephosphorylation of YAP at serine 127), suggesting that mutation(s), if any, in YAP do not affect the function of this oncogene in these cells; or YAP is not mutated in these cell lines.

Reviewer's minor concern #2: *Is there a role for TGFβ in this process? TGFβ-related proteins feature heavily in Fig. S2 where YAP linker genes are analysed in cervical cancer and the pathway is known to influence Hippo signalling (Varelas et.al, Dev Cell 2010).*

Authors' response: We did not check the role or function of TGFβ in these cells. However, a number of recent studies have shown the functional interaction between TGFβ signaling and the Hippo/YAP signaling (Fujii et. al, 2012; Beyer et. al, 2013; Attisano et. Al, 2013) and a few studies suggested TGFβ and Smads may play roles in cervical cancer progression (Karunagaran & Jinesh, 2008). This will be an area for our future study.

Reviewer's minor concern #3: *It is proposed that TGFα/EGFR signalling is involved in cervical cancer cell proliferation and migration, but is there a role for Ras? It has recently been reported that YAP1 compensates for loss of oncogenic Ras in certain contexts (Zhang et al. 2014; Shao et al. 2014; Kapoor et al. 2014). Oncogenic Ras is thought to promote YAP stabilisation via the regulation of SOCS proteins (Hong et al. 2014). Is this mechanism compatible with the proposed action of E6 on YAP in cervical cancer?*

Authors' response: Since our manuscript was under consideration by EMBO J, several reports has shown interaction between Ras and YAP. As an important part of the EGFR signaling, Ras might be involved in this process. We presented this argument in our discussion.

Reviewer's minor concern #4: *The authors state that the transfection efficiency of YAP plasmids is high on the basis of increased expression of YAP in the selected cell lines (Fig. 3e). However, the fact that endogenous YAP is expressed in these cells masks the transfection efficiency. Transfection efficiency would be more readily assessed by quantifying the percentage of cells expressing a co-transfected marker gene (i.e GFP); if this has not been done, authors should remove the reference to transfection efficiency.*

Authors' response: We did not use GFP to determine the transfection efficiency since our cells are stably transfected. These cell lines were selected with G418. The transfection efficiency should be very high because cells without vector will be eliminated by the culture system. However, we have removed the sentences about transfection efficiency in our manuscript.

Reviewer's minor concern #5. *Page 8 should read "TEADs are the major mediators of YAP transcriptional activities". In the following sentence Supplementary Fig. S2 is wrongly referenced and the subsequent sentence should be rephrased: "Moreover, network analysis shows that almost all genes interacted with YAP."*

Authors' response: Thank you; this has been corrected in the revised version.

Reviewer's minor concern #6. *Page 9 should read "and reduced the proportion of cells in G1 phase in both ME180 and HT3..."*

Authors' response: Thank you; this has been corrected in the revised version.

Reviewer's minor concern #7. *Page 10 should read "which may be attributed to multi-layered cell growth". Moreover, Fig. 3g is incorrectly referenced as Fig. 2g.*

Authors' response: Thank you; this has been corrected in the revised version.

Reviewer's minor concern #8. *Page 17 Fig. 9g is incorrectly references as Fig. 8g.*

Authors' response: Thank you; this has been corrected in the revised version.

Reviewer's minor concern #9. *Supplementary Fig. S1 and S9 denotes Ect1 cells as End1 cells. Is this a mistake or are they indeed different cell lines to Ect1 cells?*

Authors' response: End1 is another immortalized cervical epithelial cell line. This information has been added to the manuscript in the revised version.

Reviewer's minor concern #10. *Panels in Figure 1 should be organised differently. If 1g and 1h are magnified images of 1d and 1f, this should be indicated in 1d and 1f. If not, organising panels alphabetically would be advisable as, as it is, the data can be confusing.*

Authors' response: Thank you, Figure 1 has been reorganized.

Reviewer #2:

Reviewer's general comments: *The manuscript by He et al. describes the functional analysis of the Hippo signaling pathway in HPV-induced cervical carcinogenesis. YAP overexpression in cervical carcinomas is presented as well as YAP function in cervical cancer cell lines in relation to EGFR and TGFa signalling.*

This is a clearly written manuscript with an impressing amount of in silico and functional data, which are well presented. To date very little knowledge exists on the Hippo signaling pathway in cervical cancer, which underlines the novelty of the data presented. Moreover, the findings on clinical specimens presented herein are supported by recently published data by Xiao et al. (Expression of Yes-associated protein in cervical squamous epithelium lesions. Xiao H, Wu L, Zheng H, Li N, Wan H, Liang G, Zhao Y, Liang J.Int J Gynecol Cancer. 2014 Nov;24(9):1575-82.

© EMBO

doi: 10.1097/IGC.0000000000000259). This paper most likely came out after submission and needs to be incorporated in the revision. The data presented certainly contribute to our understanding of cervical cancer development, but whether it plays a central role, as suggested in the title is a matter of debate. I would suggest to omit the word central or to rephrase the title.

Authors' response: We appreciate reviewer #2 for his positive comments. We are happy that a recent paper published by Xiao *et al.* supports our work. We cited this paper and discussed their work in the revised manuscript. We also revised our title and conclusion and remove the word "central".

Reviewer's specific comments: *-Figure 1 shows immunohistochemical staining of tissue micro arrays and demonstrates an increased expression in cervical cancers. As the two cervical cancer histotypes (squamous cell carcinomas and adenocarcinomas) often display different expression patterns, it needs to be indicated how the staining patterns relate to tumor histotype. Moreover, as the functional data show a role of E6 in Hippo signalling, the regulation YAP overexpression may be related to HPV presence rather than or in addition to tumor stage. To this end it is worthwhile to know whether the normal samples were HPV-positive or negative and to learn about YAP staining in cervical cancer precursor lesions (CIN lesions). As described by Xiao et al, increased YAP expression may represent an early event also detectable in HPV-positive CIN lesions. Additionally, it is unclear whether nuclear staining was taken into account when scoring the microarrays (as was described for Figure 11).*

Author's response: The samples used in this study are commercial tissue microarrays. In keeping with the observations that around 80% of cervical cancers are squamous cell carcinoma, the tissue microarrays contains close to 94% squamous cell carcinomas and the remaining are adenocarcinoma samples (only 5 cases). So it is inaccurate to analyze YAP expression data derived from different tumor histotypes. Fortunately, in one of our ongoing projects, we will collaborate with gynecological pathologists to investigate HPV-regulated ubiquitination and deubiquitination of the key kinases of the Hippo/YAP pathway, and we hope to obatin enough different histotype tissues, including cervical intraepithelial neoplasia (CNI1, 2&3), cervical squamous cell carcinoma and endocervical adenocarcinoma.

According to our findings, we agree with reviewer that "the regulation YAP overexpression may be related to HPV presence rather than or in addition to tumor stage". Although we cannot directly confirm this inference with our own samples (because no HPV infection information was recorded in the information page of these microarrays), we are really happy to see that this was confirmed by Xiao and colleagues recently (Int J Gynecol Cancer. 2014). We will cite this paper in the revised manuscript. Our staining results showed that in the cervical tumor cells, YAP was mainly localized to the nucleus regardless of stage or grade. We used software (Aperio ImageScope) to analyze the YAP staining signal both in nucleus and cytoplasm, because we noticed that HPV increased both total YAP and phosphorylation YAP at Ser127.

Reviewer's specific comments: *I have some fundamental problems with the E6E7 immortalized endocervical and ectorcervical cell line being referred to a normal cervical cells (Fig S1 and*

further). First of all they express the viral oncogenes in dividing cells, a characteristic of high-grade CIN lesions and cancer cells and not normal cells. Moreover, they are most likely genetically instable and telomerase positive. I would suggest to refer to them as HPV-immortalized cells, rather than normal cells. It would be of interest, but probably undoable to include primary cervical cells in the analysis.

Author's response: We appreciate this suggestion. We have referred these cells as HPV-immortalized cells in the revised manuscript.

Reviewer's specific comments: *Figure 4: are the differences in colony formation statistically significant?*

Author's response: Yes, we marked this in the revised manuscript.

Reviewer's specific comments: *Data are presented on HPV-positive (ME-180) and HPV-negative cell lines (HT-3), showing similar expression levels and functional effects (Fig 1-4 and ME-180 vs suppl figures). Therefore it is questionable whether there is a specific role for E6 in YAP activation in the ME-180 cell line. For the E6 knock-down experiments shown in Figure 9 HeLa cells were used. Why not ME-180 cells? Related to this it would be interesting to know what mechanism drives YAP activation in the HT-3 cells (see Figure 12b)?*

Author's response: The characteristics of HT3 and ME180 cell lines actually support our conclusion. ME180 cells (HPV positive) grow faster than HT3 cells (HPV negative). Actually, ME180 cells grow so fast that we have to use serum-reduced medium (1%~2.5% FBS in medium) to culture these cells. HT3 cells grow slowly even in growth medium (with 10% FBS). Moreover, the HPV positive ME180 cells have higher YAP levels compared to the HPV-free HT3 cells. Furthermore, ME180 cells form tumors faster than HT3 cells in the athymic nude mice. The tumor xenografts derived from ME180 cells also grow more rapidly than that of HT3 cells.

ME180 was also derived from a highly invasive squamous cell carcinoma and is HPV positive. The cells contain HPV DNA, but the HPV type is not clear. A previous study showed the DNA is greater homology to HPV39 than HPV18 or HPV16 (Reuter et al. 1991**)**. So it is hard to design the specific siRNA to silence E6 protein in ME180 cells. We used HeLa cells in the E6 knock-down experiments because this cell line is derived from invasive cervical cancer cells and are HPV18 positive. This is now stated in the manuscript. Both YAP and E6 protein levels are higher in this cell line, making it an ideal cellular model to study HPV E6 and YAP interaction using gene knockdown techniques. Importantly, our findings have been replicated with multiple cell lines, which facilitates our ability to make reasonable conclusions

Reviewer's specific comments: *The effect of E6 on YAP phosphorylation is studied in HT-3 cells, but not E7. To strengthen a specific role of E6, E7 function needs to be studied as well as well as a combination of both.*

Author's response: We also believe that inclusion of HPV16 E7, or other HPV early gene products such as E2 and E5 proteins, may produce very interesting data. However, we also believe that expanding the current study to include HPV16 E7 and other early genes will significantly expand the manuscript and dilute the already large amount of research presented in this manuscript. Please note that we have already presented 12 busy figures and two tables in the manuscript. We have also

presented 18 figures in the supplementary information. Inclusion of even one more factor (such as E7) means that we have to use this new factors to repeat all experiments that we have done with E6 in this manuscript. Moreover, we need to assess combined effects. However, we agree with reviewer that future studies using HPV16 E7, or other HPV early gene products such as E2 and E5 proteins, may produce very interesting data.

Reviewer's specific comments: The stainings shown in Figure 11 are not very clear.

Author's response: We provided better images to present the expression level and localization of YAP in the transgenic mouse tissues.

Reviewer #3:

Reviewer's comments: *Although this pathway may indeed play an important role in the progression of cervical cancer (and many other cancers), the study mainly defines phenomena in certain cell lines that are difficult to extend to other cell lines and/or to cervical cancer in general.*

Authors' response: In this study we employed (1) **five established cervical cancer cell lines** (non-cancerous Vs. cancerous, HPV negative Vs. HPV positive); (2) **TCGA multidimensional cancer genomic datasets** (with deep sequencing data from **191** human cervical cancer patients); (3) xenograft mouse models; (4) an **established transgenic mouse model** of cervical cancer. We feel that this study is highly relevant to human cervical cancer.

Reviewer's comments: *The experiments that attempt to implicate HPV E6 in the maintenance of high YAP levels are poorly conceived and controlled, making the results difficult to interpret".*

Authors' response: We appreciate reviewer #3 for his time. However, we do not agree with the reviewer for the following reasons:

1) The observation that HPV16 E6 stabilizes YAP was an unexpected and novel finding. Before designing our experiments, we analyzed the protein structure of HPV and Hippo pathway components. Based on our analysis, we did not expect any direct interaction between the YAP and HPV16 E6 proteins, and accordingly, did not put any efforts toward this direction. However, in experiments designed to test whether E6 treatment would alter expression of YAP, we observed that treatment of HPV-negative cervical cancer cells (HT-3 cells) with E6 led to an increase in YAP protein but not YAP mRNA. This interesting finding directed us to explore the potential mechanisms behind the observed discrepancy in YAP protein and mRNA levels. As mentioned in response to reviewer #1, HPV E6 stabilizes YAP protein in a unique and complicated way (different from CK1 mechanism, which is employed by the Hippo pathway, and from the SOCS5/6 pathway, which is employed by RAS to manipulate YAP protein level). More experiments are needed for us to uncover the molecular mechanism underlying HPV E6 stabilization of YAP protein in cervical cancer cells.

2) Our experiments are well-controlled. We first used MG132, the proteasome inhibitor, to inhibit the proteasome activity. Inhibition of proteasome led to accumulation of YAP and EGFR protein in HT-3 cells (HPV-free cervical cancer cells) within 4 hours, suggesting the involvement of proteasome in YAP protein degradation. We then used cycloheximine (CHX) to inhibit protein

synthesis. We found that both YAP and EGFR protein decreased within 4 hours in cells treated with CHX. However, if HPV16 E6 was added to the culture, YAP protein level did not decrease in cells treated with CHX for 8h. These results clearly indicate that treatment with HPV16 E6 protein prevents the YAP protein from protease-induced protein degradation. This is a standard design for the protein degradation analysis (see examples: Genes & Dev. 2010. 24: 72-85; *Molecular Cell*. 2008, 29: 350–361).

3) Our manuscript has been read by several well-established scientists in this field, including Dr. John S Davis (Professor, at UNMC), Dr. Peter Angeletti (Associate Professor, expert in virology, University of Nebraska-Lincoln), Dr. Paul Lambert (Professor, top scientist in the cervical cancer research, University of Wisconsin–Madison), Dr, Kerry Rodabaugh (Professor and gynecological oncologist, UNMC), Dr. Sobudha Lele (Professor, gynecological pathologist, UNMC) and Dr. Steve Remmenga (Professor, gynecological oncologist , UNMC). Everyone believes that we have very novel and important observations that warrant publication in this Journal.

To further confirm our findings, in the revised version, we transfected HT3 cells with lentivirus-based empty vector (Ctrl) and HPV16 E6 expression vectors to establish HT3-Ctrl and HT3-E6 cell lines. Western blotting results showed that E6 expression in HT3 cells also increased the proteins level of total YAP and phosphorylated YAP (Ser127) (Fig. 10f). Our data showed that SOCS6 may be an important mediator for E6 stabilization of YAP protein in HT-3 cervical cancer cells. Moreover, we used human foreskin keratinocytes (HFKs) raft cultures, which faithfully recapitulates epithelial differentiation and HPV life cyce *in vivo*, to test the interaction between HPV16 and YAP. We found the signal intensity of YAP in HPV16 raft cultures was much higher than the normal HFKs raft cultures (Fig. 11g, 11h, 11i & 11j). More importantly, the staining results revealed intense YAP signals in a high percentage of mid-to upper spinous cells (Fig. 11h). The increase in YAP signal in these cells is identical to both the pattern consistently observed in naturally occurring HPV lesions and distribution of HPV DNA in HFKs raft cultures (Stoler & Broker, 1986; Wang et al, 2009), further supporting the conclusion and clinical relevance of this study.

Reviewer's comments: *Overall, the use of cell lines more typically employed in Hippo pathway studies, perhaps compared to some of these cervical cancer cell lines, would have made the study more useful.*

Authors' response: *W*e think that this suggestion is ambiguous. First, the present study is focused on cervical cancer. HPV causes more than 99% of the cervical squamous cell carcinomas. Therefore, it is appropriate to use cervical cancer cells. The Hippo pathway has been proven to play critical roles in many different types of organ and tissues. One pioneering study in this field was conducted by our co-author, Dr. Jixin Dong. He used drosophila and liver cells to investigate the role of YAP in organ size control and tumorigenesis (Dong et al. *cell*, 2007, 130: 1120-1133). However, we do not believe that drosophila and liver hepatocytes have clinical relevance to cervical cancer and HPV infection. Our studies used HT-3 cervical cell line (HPV negative) and ME-180 cervical cells (HPV positive), which we feel are relevant for cervical cancer research.

Reviewer's comments: *The Hippo pathway has been well defined in several cell types, including MCF10A cells. Has the pathway ever been examined in any of the cell types used here? Have any of*

the findings presented here been tested in MCF10A or other well-established cell models, either by this group or other researchers?

Authors' response: in the introduction, we indicated that the Hippo/YAP signaling pathway has not been investigated in cervical cancer. Therefore, the study provides novel information about this important pathway in cervical cancer using five immortalized and cancerous cervical epithelial cell lines. In the present studies, our main focus was the role of the Hippo/YAP pathway in cervical cancer and the potential interaction YAP and HPV, the major causative agents of cervical cancer. We do not believe that using MCF10A (an immortalized human mammary gland epithelial cell line) is a useful model for cervical cancer. Importantly, a previous study suggested that YAP may function as a tumor suppressor in certain types of mammary cancer (Yuan et al, 2008).

Reviewer's comments: *Figures 3-4 and Figures S3-S6: The authors observe that YAP overexpression drives increased cellular proliferation and migration. Indeed, this is a consequence of YAP overexpression that has been appreciated since YAP was first identified as an oncogene (Overholtzer et al, PNAS 2006). The observation that YAP overexpression drives increased proliferation of the cell lines characterized here is not surprising.*

Authors' response: As stated above, currently there is nothing known about the role of YAP in cervical cancer. YAP functions as an oncogene in some tissues and a tumor suppressor in others. Therefore, identifying components of the Hippo/YAP signaling pathway and provide novel information on its regulation and function in cervical cancer is new and makes an important contribution to the field. Importantly, our focus was not just that YAP promotes cervical cancer cell proliferation; studies in this manuscript provide new insight on a previously unprecedented mechanism underlying cervical cancer initiation and progression.

Reviewer's comments: *Figures 6-7, S7-S10. The link proposed by the authors between YAP signaling and TGFalpha expression many be very indirect. They extend this to perform a series of experiments in which various cancer cells are treated with TGFalpha and in general, are more proliferative after TGFalpha treatment. This does not seem to be a surprising result nor does it add much to an understanding of YAP and/or TGFalpha signaling. Do the authors mean to imply that this is the case only in cervical cancer cells?*

Authors' response: Our study indicated that expression of YAP or constitutively active YAP in cervical cancer cells lead to significant increase in expression of EGFR and its two ligands, TGFα and AREG. Indeed, we found that treatment of cervical cancer cells with TGFα and AREG stimulated proliferation and migration. Importantly, we also report in our study that the stimulatory actions of TGFα on the cervical cancer cells was dependent on the presence of YAP. This evidence, together with the observation that TGFα and AREG, via activating EGFR, suppressed the Hippo pathway and activated YAP protein, clearly indicate that the EGFR signaling pathway and the Hippo/YAP signaling pathway interact to form an autocrine loop that promotes cervical cancer cell proliferation. As mentioned above, in the revised version, additional experiments (cellular model, xenograft model, as well as large-scale patient sequencing data) has been presented to further support the existence and function of the proposed feed-forward loop.

© EMBO

The finding that HPV E6 stabilizes the YAP protein connects this feedback loop with the cervical cancer causative agent and strengthens the clinical relevance of our finding. In the context of the whole study, we conducted a logical series of experiments and have provided mechanistic data showing how the Hippo/YAP pathway contributes to cervical cancer cell proliferation.

Reviewer's comments: *Figure 1: The results section corresponding to this figure should begin with a description of the experiment, rationale, and source of tissue.*

Authors' response: Although this information was presented in the MATERIALS AND METHODS section we have added additional description in the Results section as requested.

Reviewer's comments: *Figure S1 and throughout the work: End1 cells are endocervical cells that have been immortalized via the introduction of HPVE6 and E7. These are a poor choice as a negative control for experiments examining HPV-associated cancers, as they express the transforming proteins of HPV16. Later in the study, the authors highlight the observation that E6 induces an increased level of YAP protein - if this is the case, an E6-negative control should be included in this experiment and in all other experiments.*

Authors' response: End1 cells are immortalized, "non-tumorigenic" cervical epithelial cells that express HPV proteins. We used the End1 cells in select experiments. We observed that overexpression of YAP induced colony formation in these cells. This experiment successfully verified our observations that YAP is an oncogene in the cervical epithelial cells. As shown in our study we also employed HT3, one of the few available HPV negative cervical cancer cell line. We used these cells to show that HPV16 E6 increased the YAP protein. In the revised version, we defined the cell types more clearly in the results section to clarify the rationale for the experiments.

Reviewer's comments: *Figure 9A-E, Figure 10: Here, the authors use an unorthodox method to express HPV16 E6 in cells - they treat the cells with recombinant E6 for as little as 1 hr. Presumably, they attribute the effects they observe to the intracellular functions of E6. What is the evidence that E6 is internalized into the target cells and that it is functional once there? Many more controls are needed and a different system of E6 expression would be preferable. There is also no attempt to examine the effects of HPV E7 nor is there a reason given for the focus on E6.*

Authors' response: Recombinant HPV16 E6 protein has been frequently used in cervical cancer research (*The Journal of Immunology,* 2001, 167: 497–504; Cancer research, 1999, 59: 1184–1187). These recombinant HPV16 E6 and E7 proteins are translocated to the nucleus within minutes (*Journal of Virology*, 2003, 77: 2330–2337). In this study, we treated cells with recombinant HPV16 E6 protein for multiple time points (1h, 8h, 24h, 48h and 96h). We found that E6 was able to affect the protein level of YAP as early as 1h. Therefore, we used 1h time point to determine if HPV16 E6 acutely stimulates YAP gene transcription (mRNA). We also used 8h and 48h to determine the effect of E6 on YAP mRNA expression (Fig 10). For YAP protein expression, we treated HT3 cells with HPV16 E6 for 48h. Moreover, we confirmed our findings by knocking down E6 protein with E6 siRNA in HeLa cells.

As descripted above, in the revised version, we used lentivirus-based E6 expression vector and human foreskin keratinocytes (HFKs) raft cultures to confirm our findings.

Reviewer's comments: *Figure 9F-H: For how long were the cells treated with siRNAs? The 6h treatments described for other experiments in the Materials and Methods may be much too short to have any meaningful effect on protein levels.*

Authors' response: Sorry for any confusion; the siRNA transfection procedure typically takes about 6h (depending on the siRNA used). The cells are allowed a 24h recovery time before treatment. The treatment time of recombinant HPV16 E6 is 48h or 72h after siRNA transfection. However, our data showed that 8h treatment of HT-3 cells with recombinant HPV16 E6 can affect the protein levels of YAP (see Fig 10d).

Reviewer's comments: *Figures 6-7, S7-S10. Also, siGLO is not an appropriate non-targeting control in siRNA experiments, as it is not incorporated into or processed by the RISC complex. It is useful only as a transfection control.*

Authors' response: When we first perform knockdown experiments, we usually use five controls to optimize our transfection efficiency. These controls include: the non-transfected cells, the siGLO RISC-free negative control group, the lipofectene2000 group, the target siRNA only group, and lipofectene + target siRNA group. These pre-transfection tests have shown that siGLO negative control has no effect on gene expression, cell viability and cell proliferation (as reported by the manufacture. http://dharmacon.gelifesciences.com/sirna/siglo-risc-free-control/). Another advantage of using siGLO RISC-free negative control is that it serves not only as a non-targeting siRNA control, but also a transfection efficiency marker. siGLO non-targeting siRNA makes it possible for us to monitor transfection efficiencies between experiment groups and necessary repeats.

3rd Editorial Decision 04 August 2015

Thank you for the submission of your revised manuscript to EMBO Molecular Medicine. We have now received the enclosed reports from the referees that were asked to re-assess it.

As you will see the reviewers are now globally supportive (aside for a few issues that I will mention below) and I am pleased to inform you that we will be able to accept your manuscript pending the following final amendments:

1) Reviewer 1, while now satisfied that the manuscript has improved substantially, it still not happy with the provided western blotting experiments (referred to in his/her original revaluation). S/he acknowledges the provision of quantification data, but would like to see improved quality of data or blots that are more representative of the quantification data. Reviewer 2 is also now satisfied, but would like you to discuss the potential role of other viral proteins. Provided you deal with both issues carefully, I will be able to proceed with an editorial decision.

2) Please provide "The Paper Explained" and "Author Contributions" sections (http://embomolmed.embopress.org/authorguide).

3) As per our Author Guidelines, the description of all reported data that includes statistical testing must state the name of the statistical test used to generate error bars and P values, the number (n) of independent experiments underlying each data point (not replicate measures of one sample), and the actual P value for each test (not merely 'significant' or 'P < 0.05').

4) We are now encouraging the publication of source data, particularly for electrophoretic gels and blots, with the aim of making primary data more accessible and transparent to the reader. Would you be willing to provide one PDF file per figure that contains the original, uncropped and unprocessed scans of all or at least the key gels used in the manuscript? The PDF files should separated by figure and be labeled with the appropriate figure/panel number, and should have molecular weight markers; further annotation may be useful but is not essential. The PDF files will be published online with the article as supplementary "Source Data" files. If you have any questions regarding this just contact me.

5) I note that the quality of some images is not ideal. The resolution appears low especially for figures 10 (h), and 11 (i, j) Please provide better images to avoid problems with production.

6) We now provide enhanced supplementary information for our readers (Expanded view). Please carefully read and adhere to our guidelines for the provision of supplementary information (http://embomolmed.embopress.org/authorguide#expandedview).

7) Please remove the red lettering from the manuscript, as it is no longer needed.

Please submit your revised manuscript as soon as possible.

I look forward to reading your final revised manuscript.

***** Reviewer's comments *****

Referee #1 (Remarks):

In their revised report, the authors provide additional data to address several of the major points raised in my first review. Specifically, they provide further evidence that the Hippo signaling pathway is involved in their proposed mechanism (by addressing the effect of LATS siRNA), and there is additional evidence suggesting that YAP is working with TEADs in this context (by virtue of the use of an inhibitor of YAP-TEAD interaction). Moreover, there is more detail regarding the mechanism by which HPV E6 regulates YAP levels and a role for SOCS5/6 is proposed.

However, despite the inclusion of quantification data for several WB-based experiments that were not entirely convincing, in some cases the blots themselves seem to still be the original ones, albeit reformatted. I would have preferred the inclusion of entirely new blots or ones that clearly reflect the quantification.

Overall, the revised manuscript contains sufficient data that enhances its original message and therefore merits publication in EMBO Molecular Medicine. Minor revisions regarding the aforementioned WB data would be advisable.

Referee #2 (Remarks):

The authors responded well to the reviewers comments and added additional data supporting a role of the Hippo/YAP pathways in cervical carcinogenesis.
Although the focus was on E6, a potential role of other viral proteins cannot be excluded, as was also stated in the response letter. I understand that testing other viral proteins is out of the current scope, but would suggest to add a remark in the Discussion.

2nd Revision - authors' response 18 August 2015

I really appreciate your decision that our manuscript are acceptable for publication in *EMM*. We also appreciate reviewers for their time and constructive suggestions. We have addressed two minor concerns from reviewers in the revised version. We also reformatted our manuscript according to *EMM* "author guidelines". Below is a list of major revisions that we have made to improve our manuscript.

1. Reviewer 1, while now satisfied that the manuscript has improved substantially, it still not happy with the provided western blotting experiments (referred to in his/her original revaluation). S/he acknowledges the provision of quantification data, but would like to see improved quality of data or blots that are more representative of the quantification data. Reviewer 2 is also now satisfied, but would like you to discuss the potential role of other viral proteins.

Authors' response: According to suggestions from editor and reviewers, we have replaced following blots:

1) Figure 9d: p-YAP (S127); total YAP; β-actin.
2) Figure 10c: total YAP, EGFR, and β-actin in both CHX and MG132 groups.
3) Figure 10d: total YAP
4) Figure 10h: total YAP; SCCS6; p-YAP (s127); P-YAP (s397); β-actin.
5) Figure S10a (Figure EV2a in revised manuscript): AREG; EGFR.
6) Figure S13b (Appendix figure S10b in revised manuscript): p-YAP (S127).
7) Figure S14 (Appendix figure S11 in revised manuscript): p-YAP (S127).

Following reviewer's suggestion, we have modified the discussion section to reveal potential role other high risk HPV viral proteins.

We believe that these modifications have greatly improved data quality of this manuscript.

2. Please provide "The Paper Explained" and "Author Contributions" sections (http://embomolmed.embopress.org/authorguide).

Authors' response: Added to the main text.

3. As per our Author Guidelines, the description of all reported data that includes statistical testing must state the name of the statistical test used to generate error bars and P values, the number (n) of independent experiments underlying each data point (not replicate measures of one sample), and the actual P value for each test (not merely 'significant' or '$P < 0.05$').

Authors' response: We have added the name of the statistical test, the number of independent experiments, and the actual P value information in the figure legends.

4. We are now encouraging the publication of source data, particularly for electrophoretic gels and blots, with the aim of making primary data more accessible and transparent to the reader. Would you be willing to provide one PDF file per figure that contains the original, uncropped and unprocessed scans of all or at least the key gels used in the manuscript? The PDF files should be separated by figure and be labeled with the appropriate figure/panel number, and should have molecular weight markers; further annotation may be useful but is not essential. The PDF files will be published online with the article as supplementary "Source Data" files. If you have any questions regarding this just contact me.

Authors' response: We are happy to provide the source data. Several PPT or PDF files with the original electrophoretic gels and blots has been uploaded with the revised manuscript (a total of 87 original images).

5. I note that the quality of some images is not ideal. The resolution appears low especially for figures 10 (h), and 11 (i, j) Please provide better images to avoid problems with production.

© EMBO

Authors' response: Thanks for your reminder. As mention above, Figure 10h has been replaced with high quality blots. Images in Figure 11 I and figure 11J have been replaced with larger and higher-resolution images.

6. We now provide enhanced supplementary information for our readers (Expanded view). Please carefully read and adhere to our guidelines for the provision of supplementary information (http://embomolmed.embopress.org/authorguide#expandedview).

Authors' response: We are happy that *EMM* provide a "the Expanded view" section to improve the accessibility, visibility and utility of some very important data that are embedded in the supplementary information. We have selected five figures to be presented in the Expanded View. The supplementary figure now reduced to thirteen. The number of the supplementary figures has been adjusted accordingly in the supplementary information (appendix figures) and articles.

7. Please remove the red lettering from the manuscript, as it is no longer needed.

Authors' response: Done

Thanks again for considering our manuscript.
