# Supplementary figures and images for: The Hippo/YAP pathway interacts with EGFR signaling and HPV oncoproteins to regulate cervical cancer progression
Source: EMBO Mol Med. 2015 Sep 28;7(11):1426–49. doi: 10.15252/emmm.201404976 (PMC4644376; doi:10.15252/emmm.201404976)

## Source Data Figures

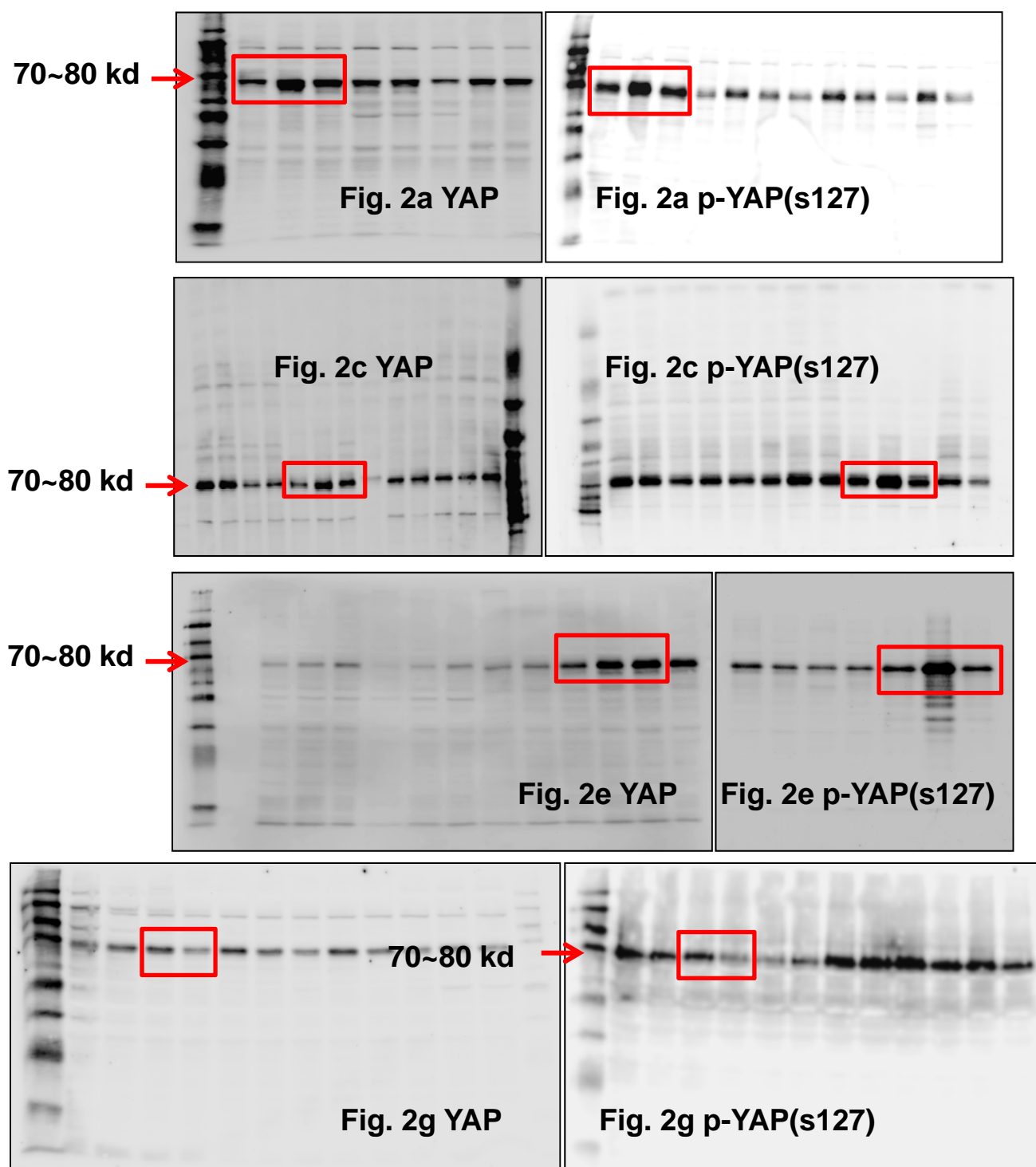

Source Data for Figure 2

Supplement: Supplementary file 4 [file emmm0007-1426-sd4.pdf]

Source Data for Figures

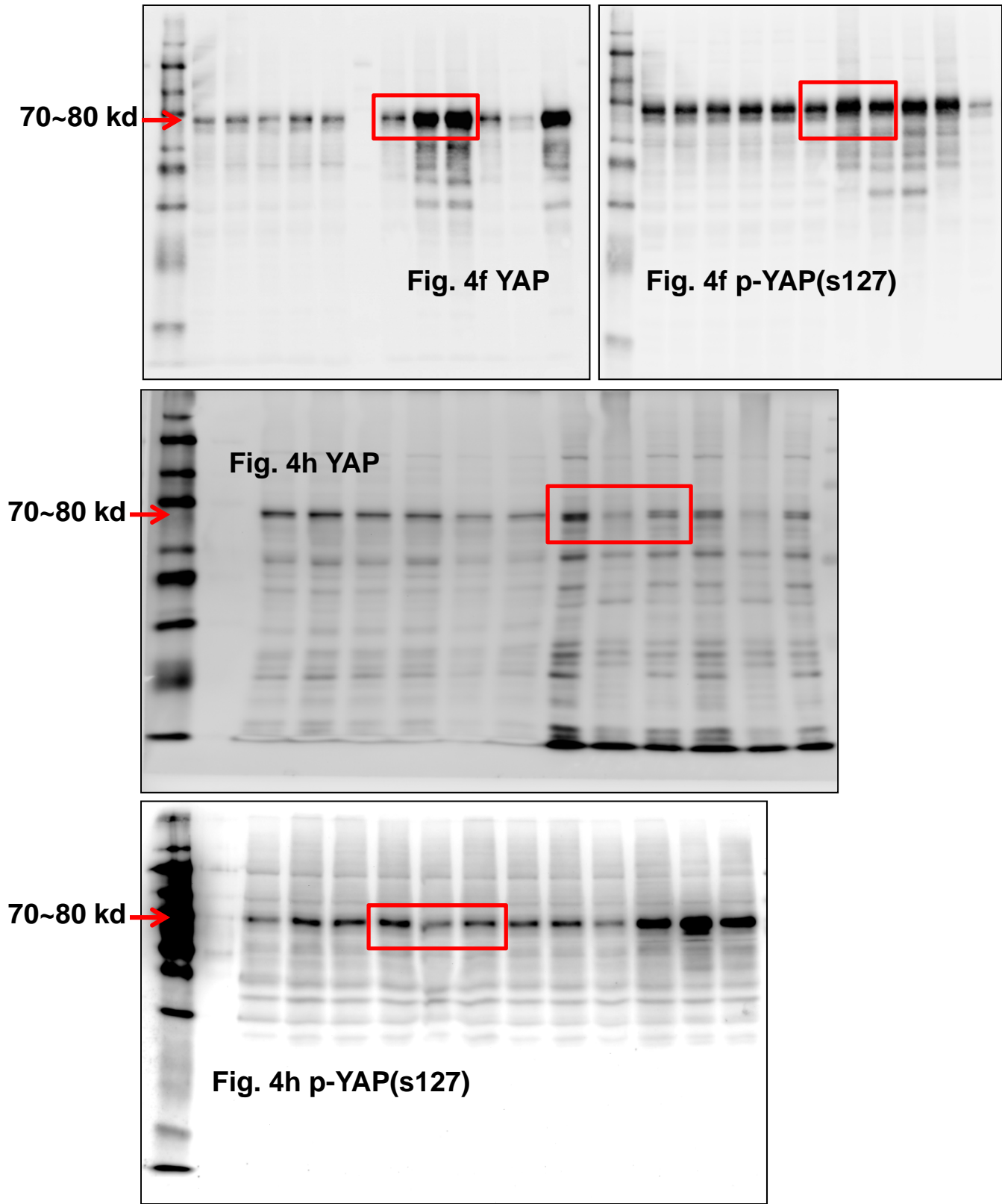

Source Data for Figure 4

Supplement: Supplementary file 5 [file emmm0007-1426-sd5.pdf]

## Source Data for Figures

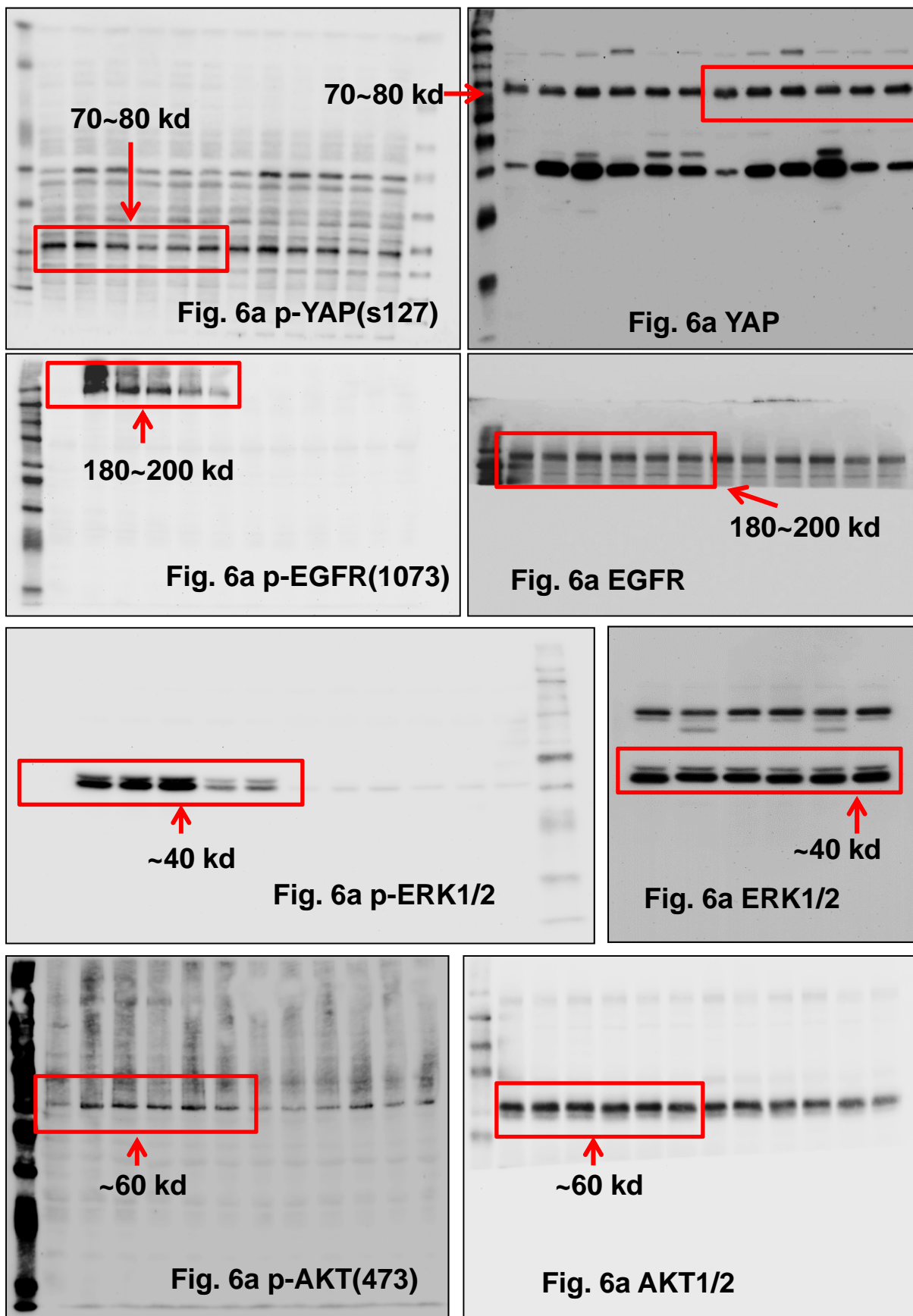

Source Data for Figure 6

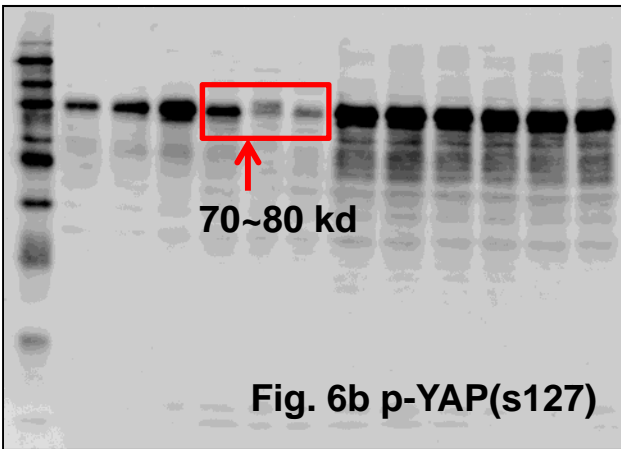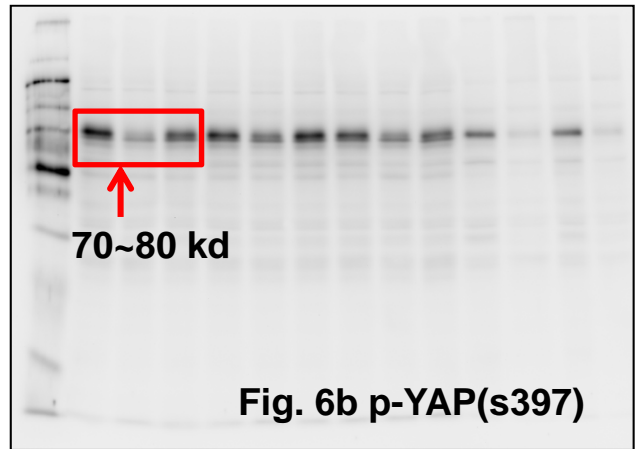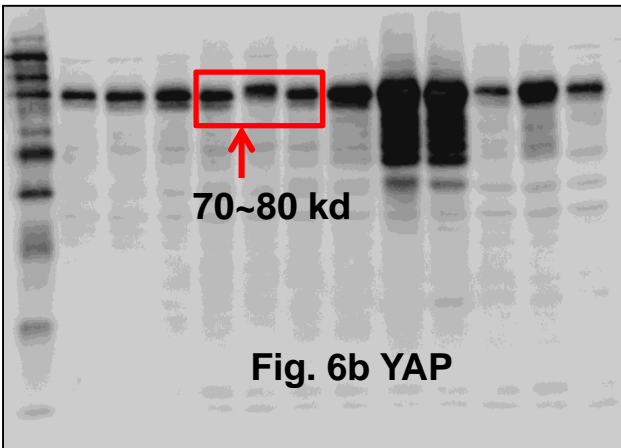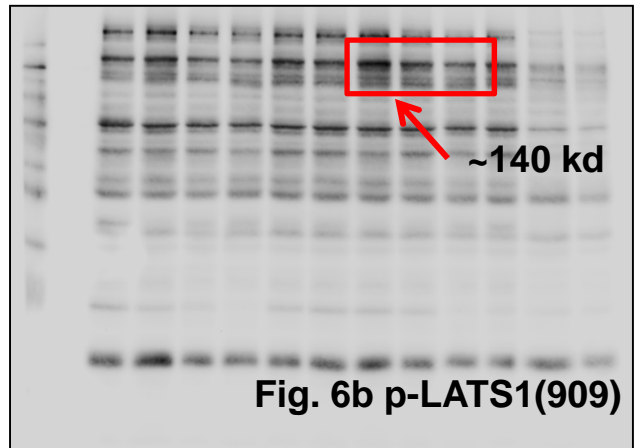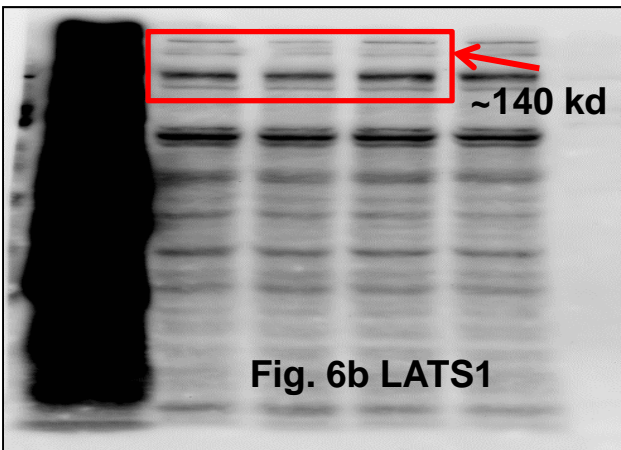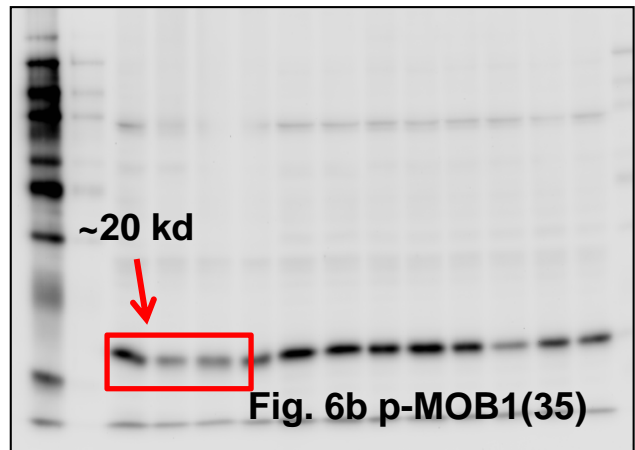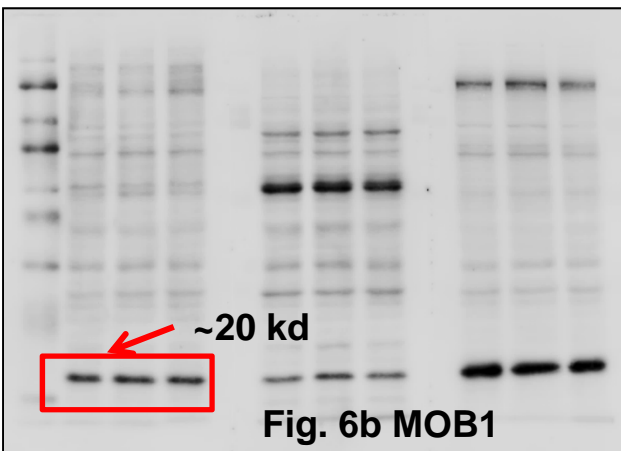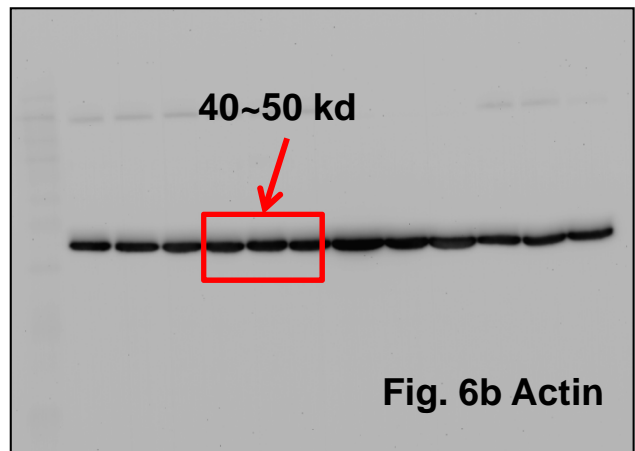

Source Data for Figure 6

Supplement: Supplementary file 6 [file emmm0007-1426-sd6.pdf]

## Source Data for Figures

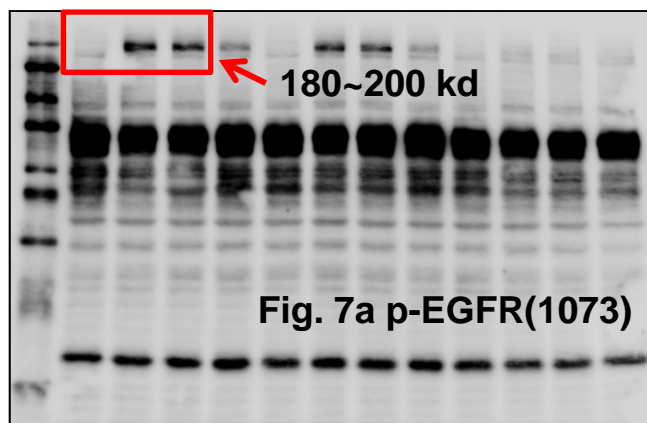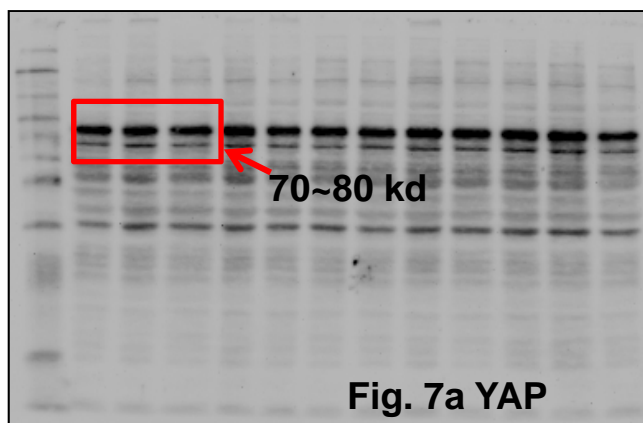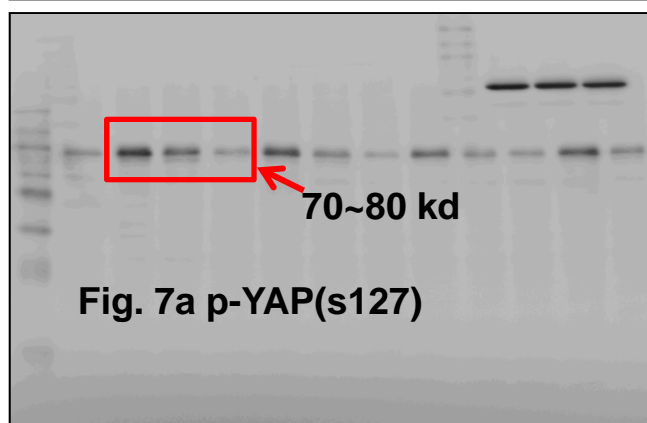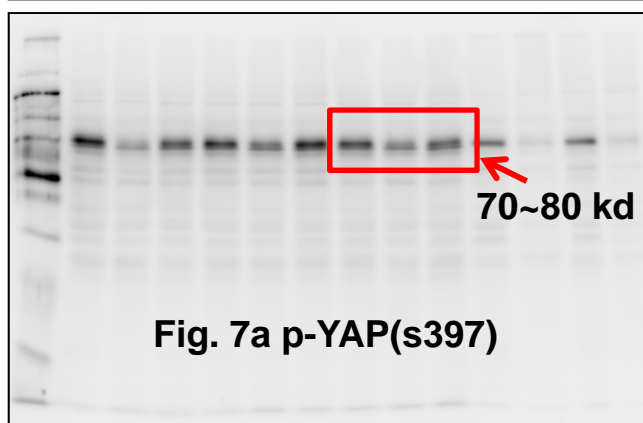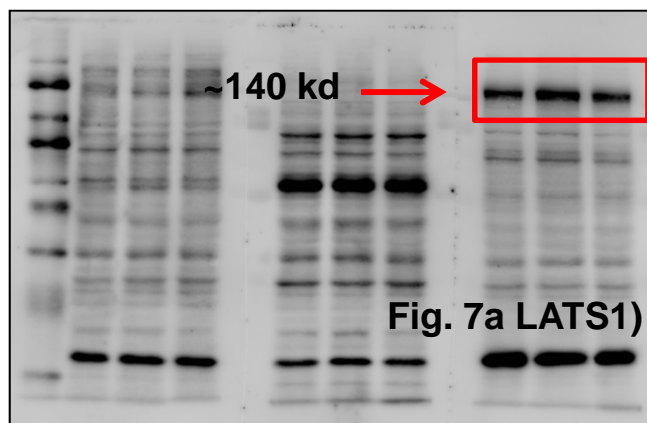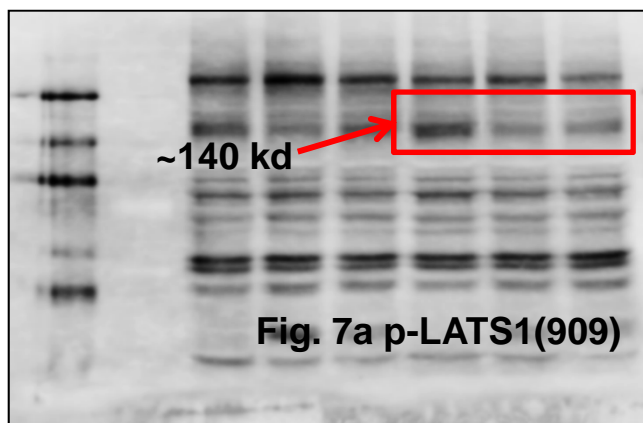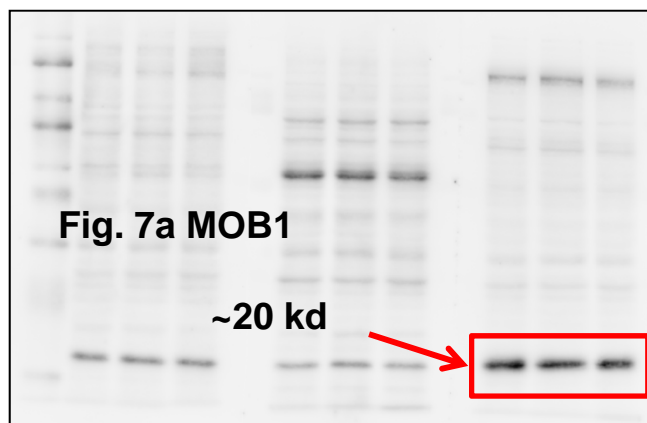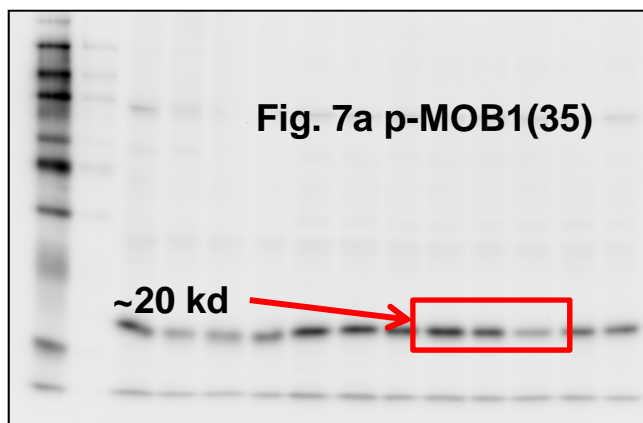

Source Data for Figure 7

Supplement: Supplementary file 7 [file emmm0007-1426-sd7.pdf]

## Source Data for Figures

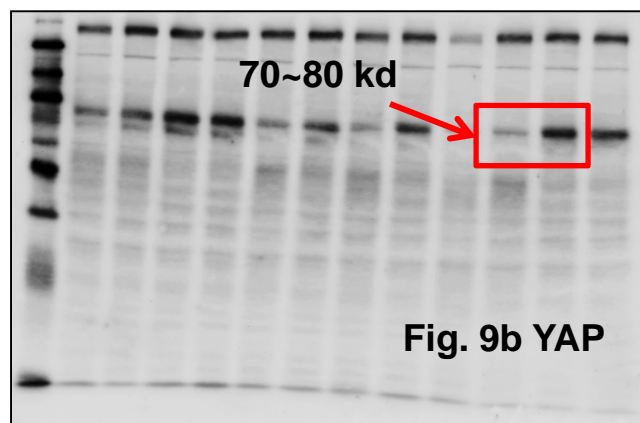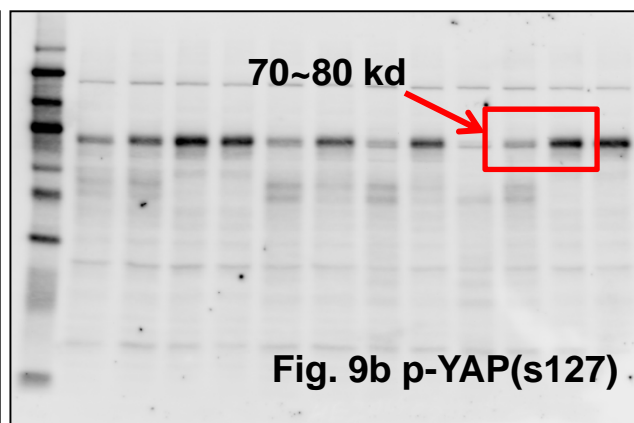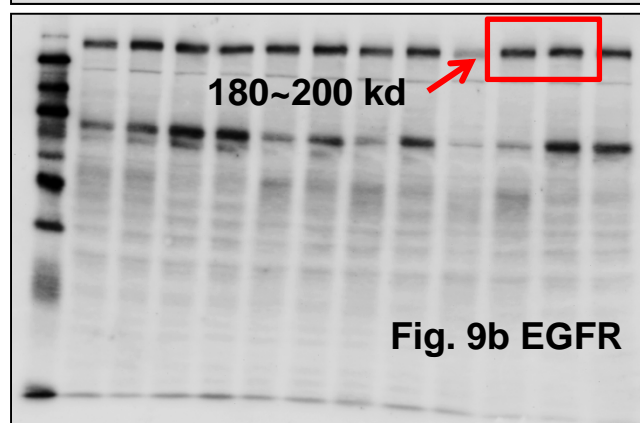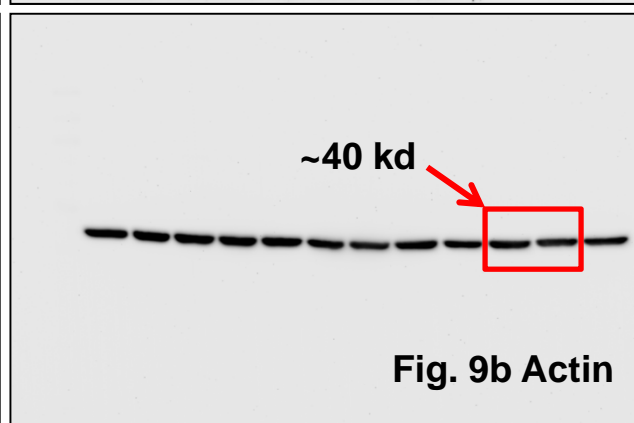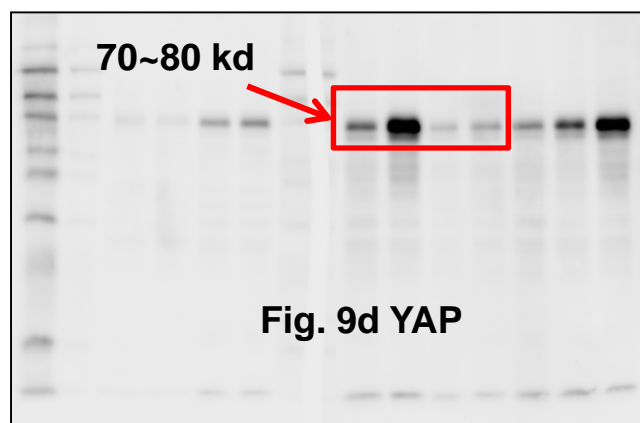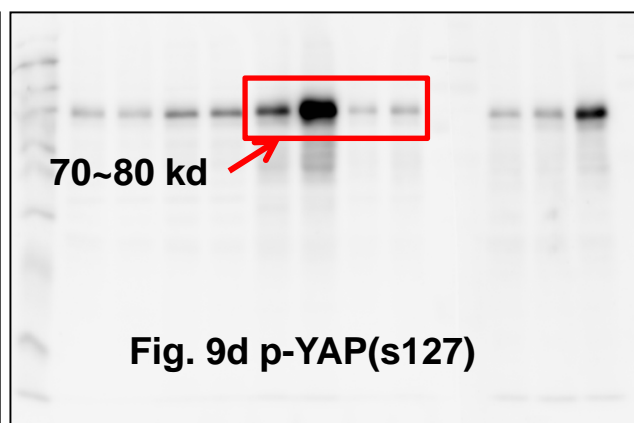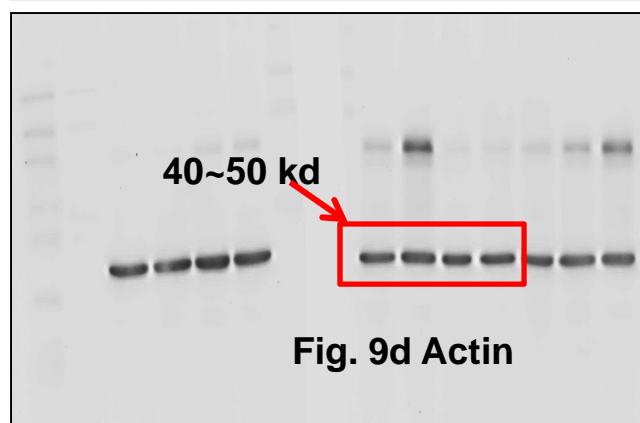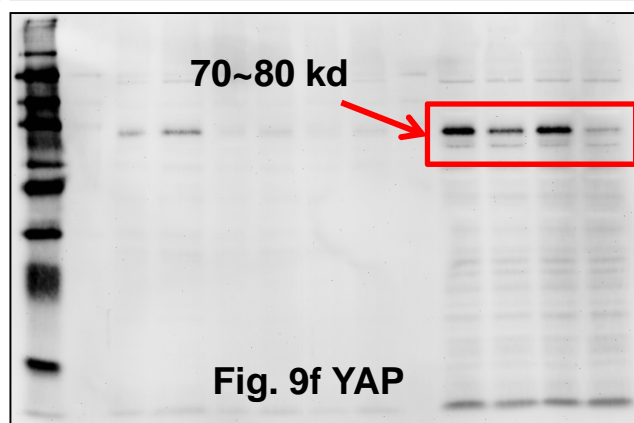

## Source Data for Figure 9

Supplement: Supplementary file 8 [file emmm0007-1426-sd8.pdf]

Source Data for Figures

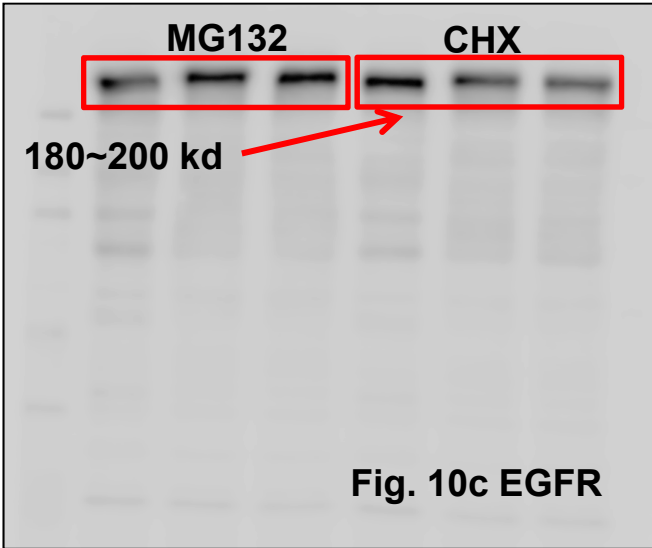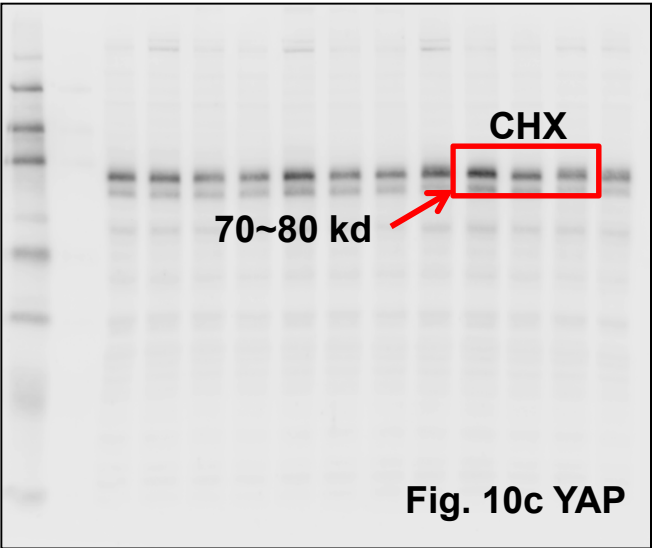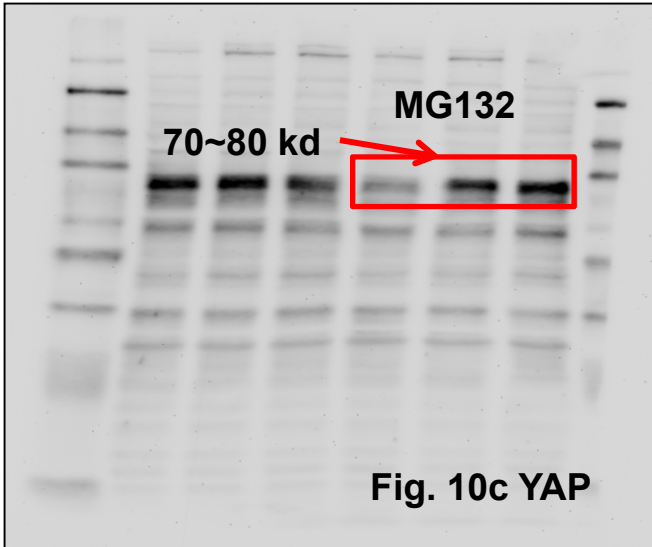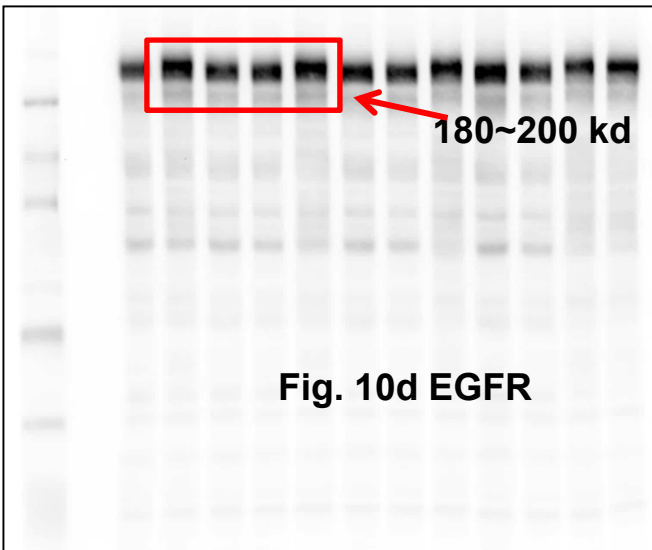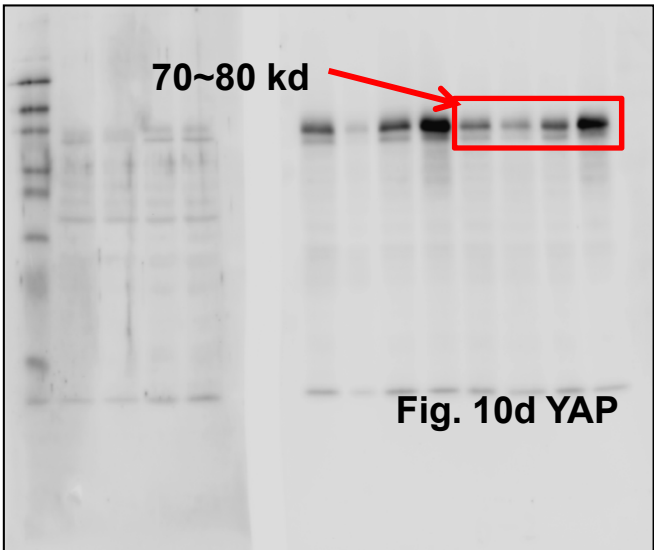

Source Data for Figure 10

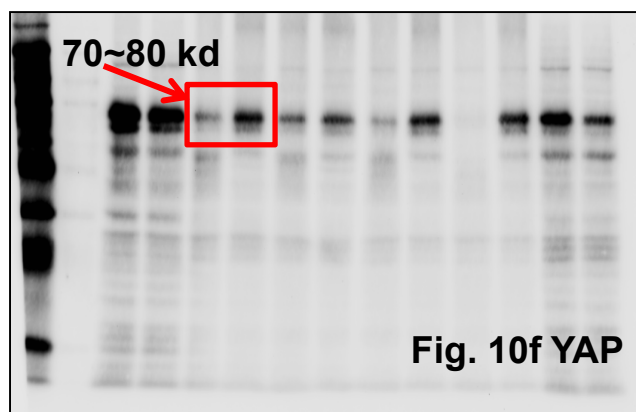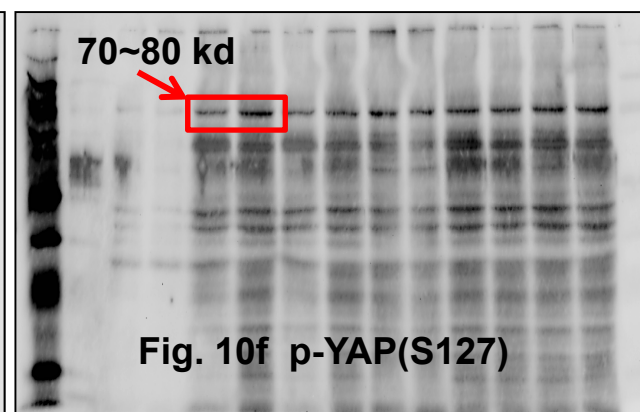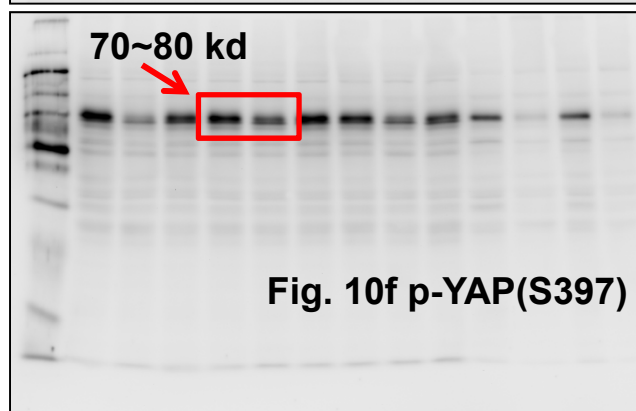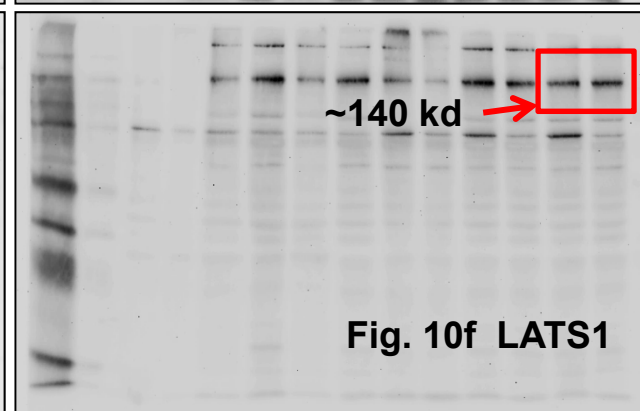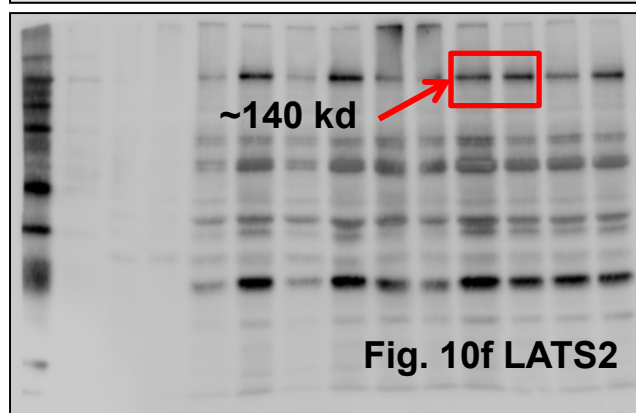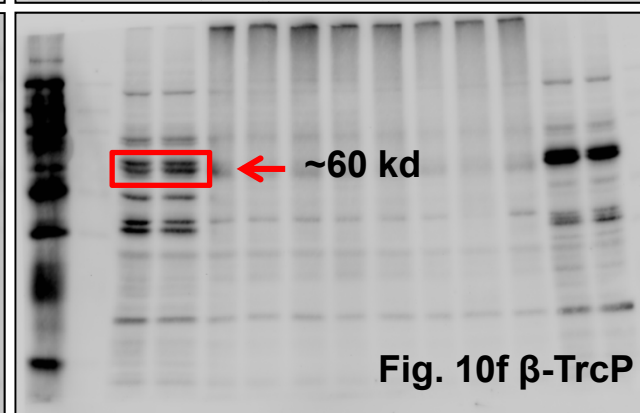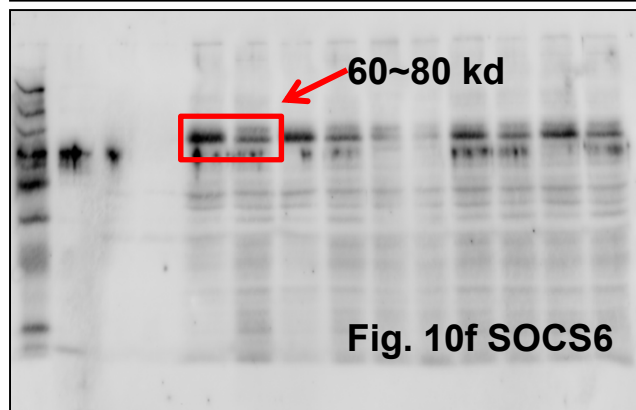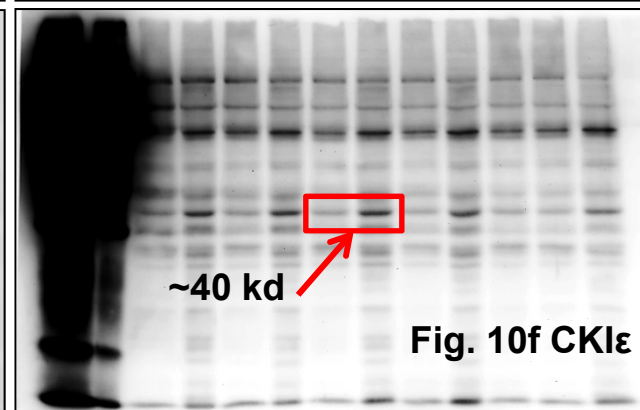

Source Data for Figure 10

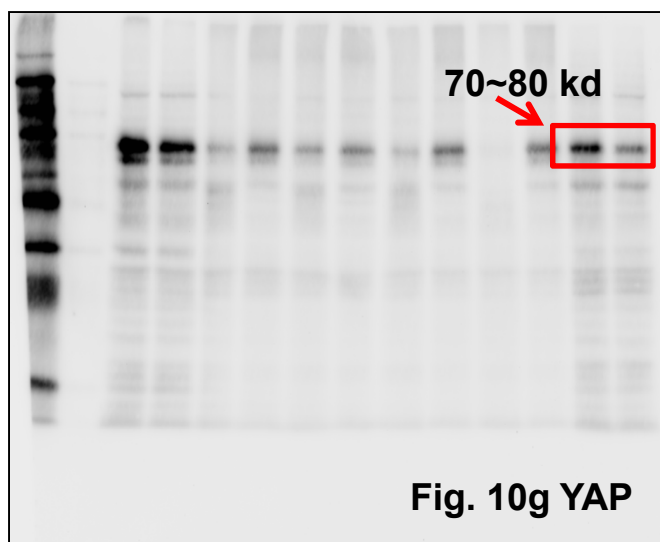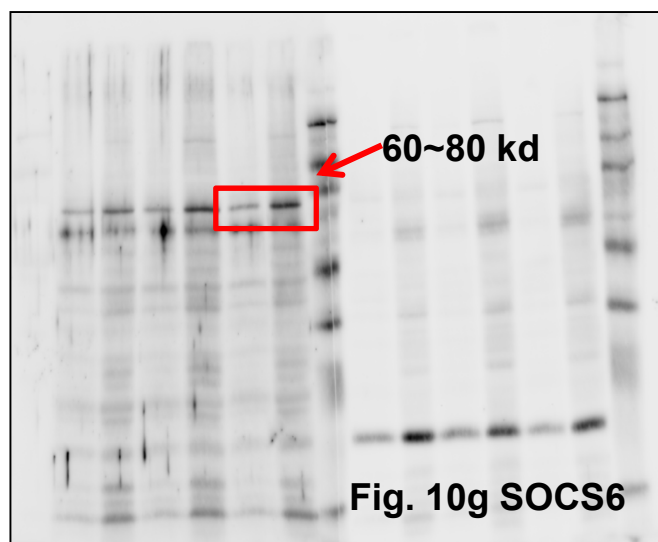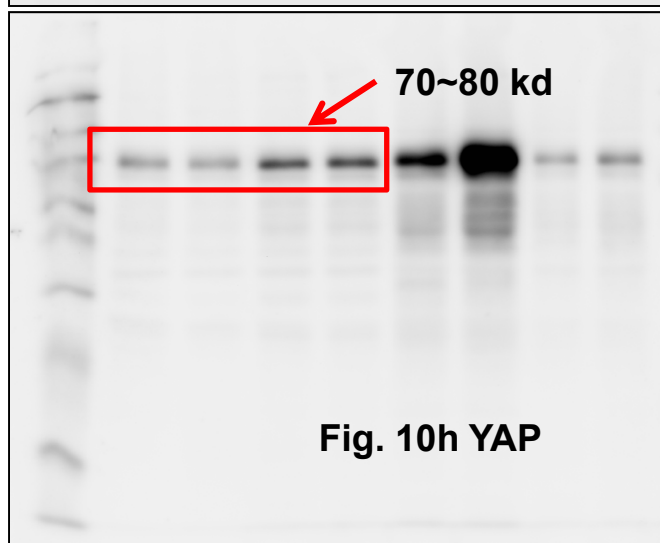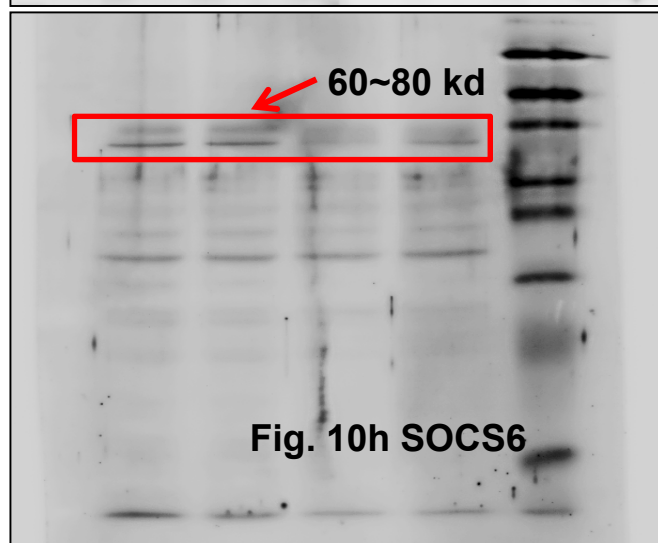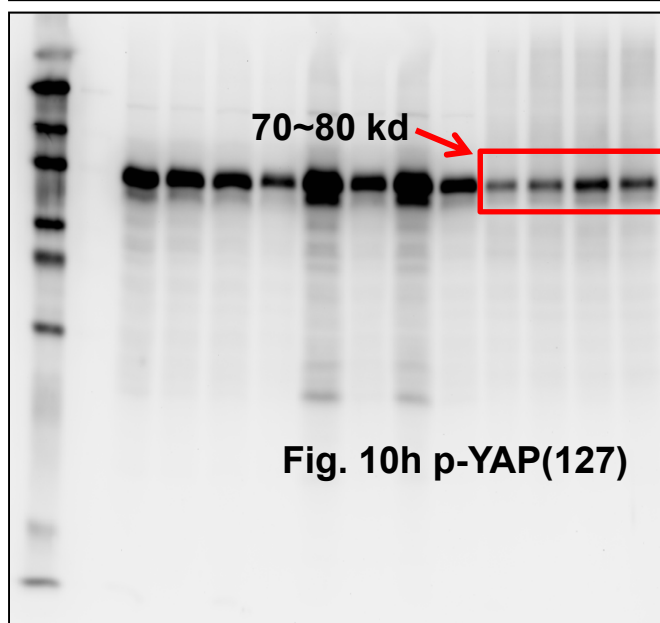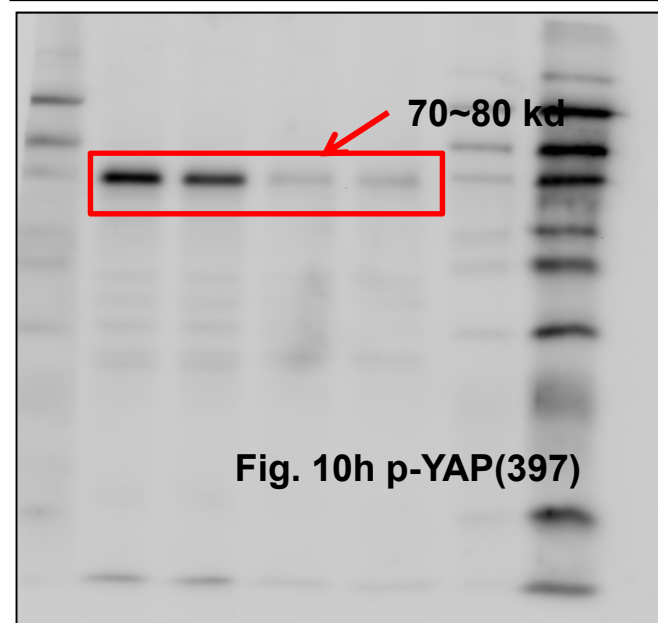

Source Data for Figure 10

Supplement: Supplementary file 9 [file emmm0007-1426-sd9.pdf]

## Source Data for Expanded View

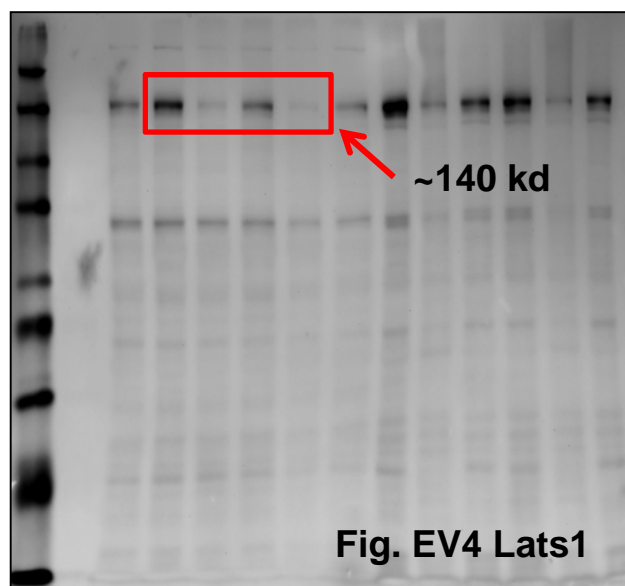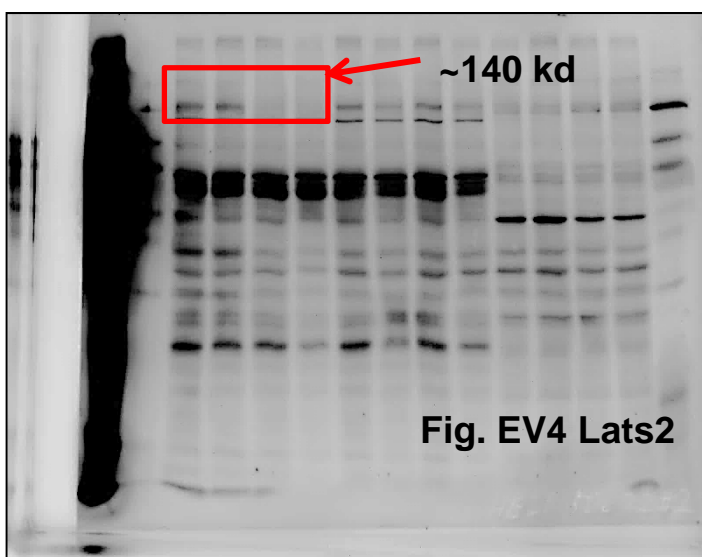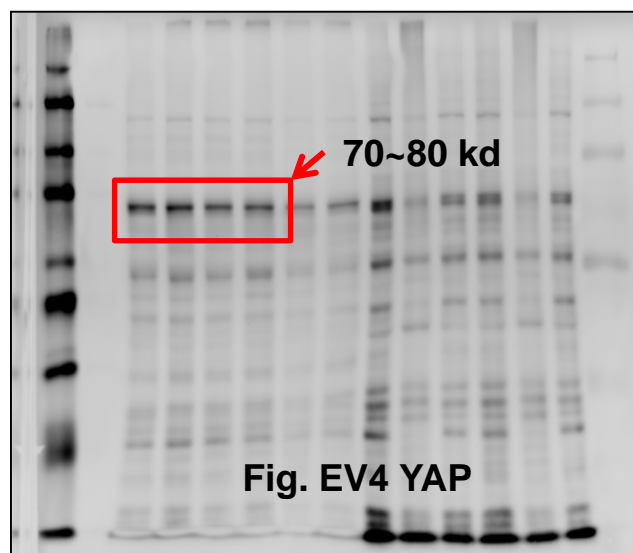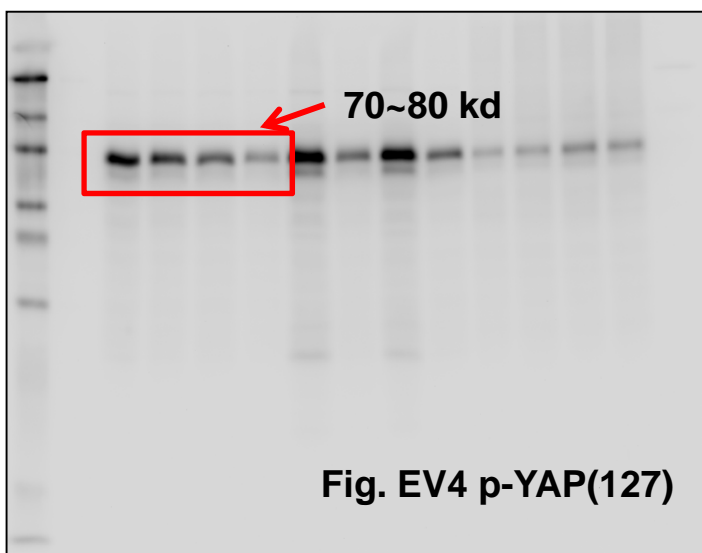

Source Data for figure EV4

Supplement: Supplementary file 10 [file emmm0007-1426-sd10.zip › Source_data_for_Expanded_View_and_Appendix/Source_Data_Fig_EV4.pdf]

Source Data for Appendix

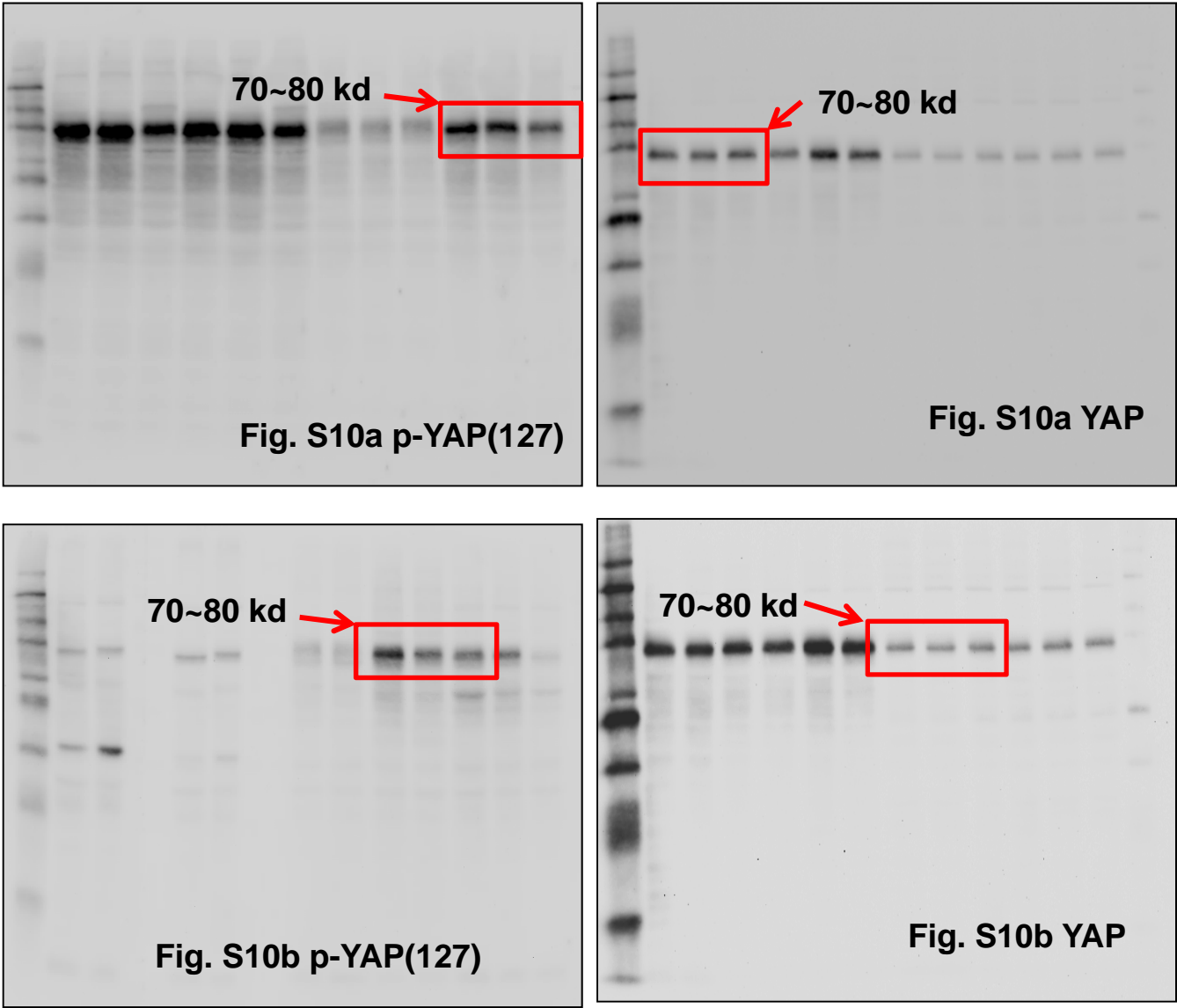

Source Data for Appendix figure S10

Supplement: Supplementary file 10 [file emmm0007-1426-sd10.zip › Source_data_for_Expanded_View_and_Appendix/Source_Data_for_Appendix_figure_S10.pdf]

Source Data for Appendix

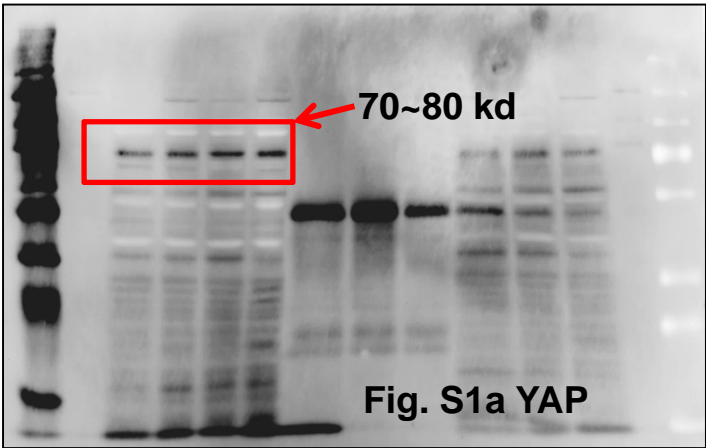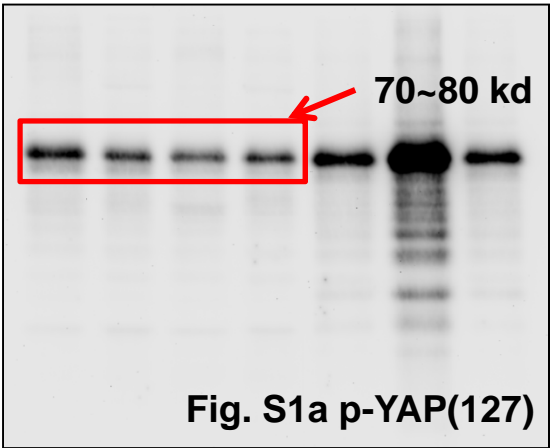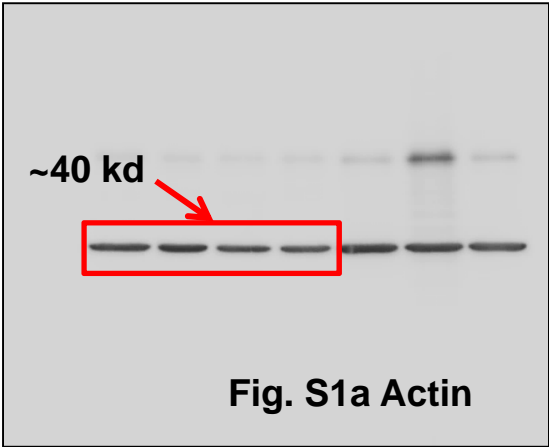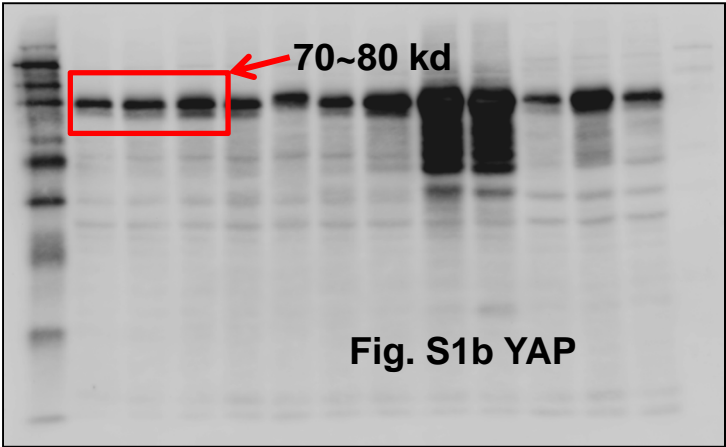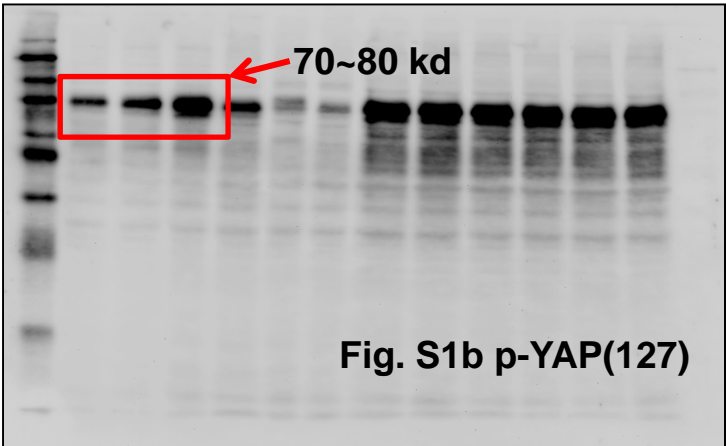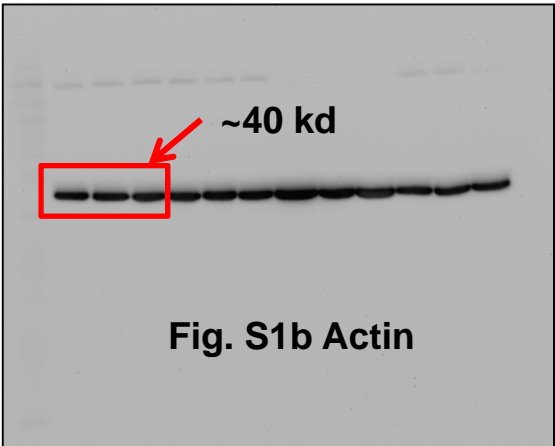

Source Data for Appendix figure S1

Supplement: Supplementary file 10 [file emmm0007-1426-sd10.zip › Source_data_for_Expanded_View_and_Appendix/Source_Data_for_Appendix_figure_S1.pdf]

Source Data for Appendix

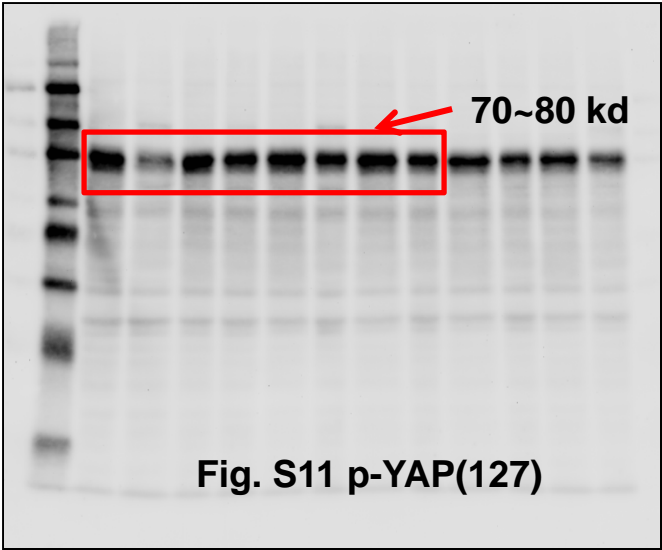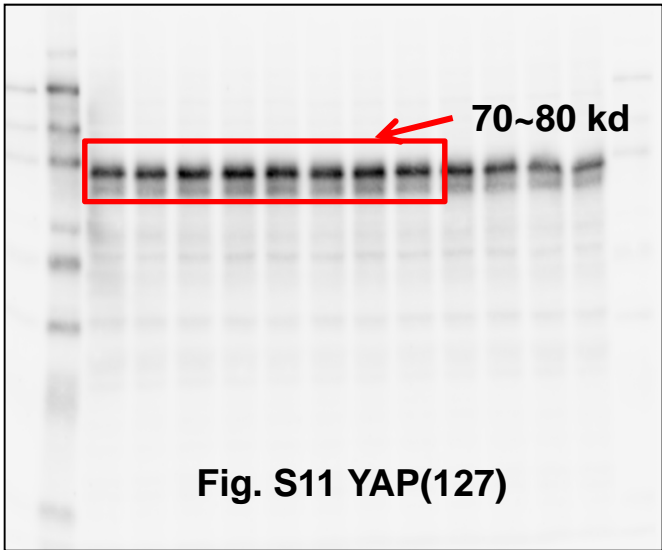

Supplement: Supplementary file 10 [file emmm0007-1426-sd10.zip › Source_data_for_Expanded_View_and_Appendix/Source_Data_for_Appendix_Figure_S11.pdf]

## Source Data for Expanded View

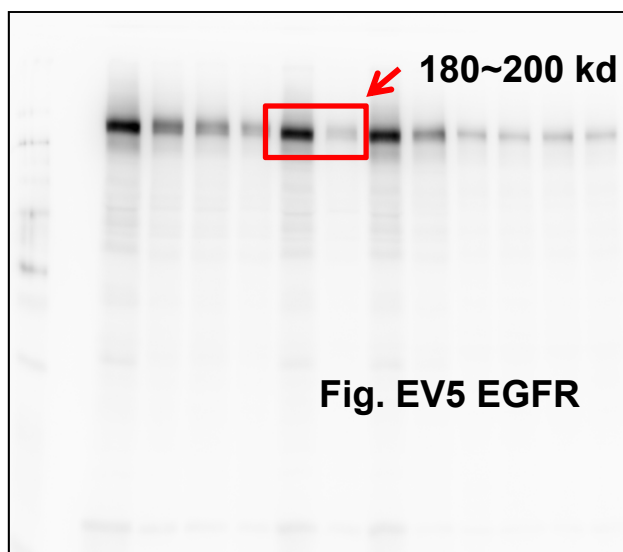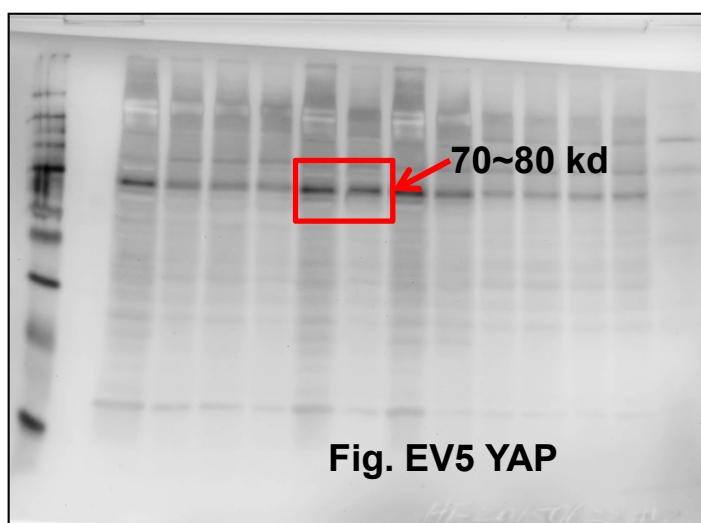

Source Data for figure EV5

Supplement: Supplementary file 10 [file emmm0007-1426-sd10.zip › Source_data_for_Expanded_View_and_Appendix/Source_Data_Fig_EV5.pdf]

Source Data for Appendix

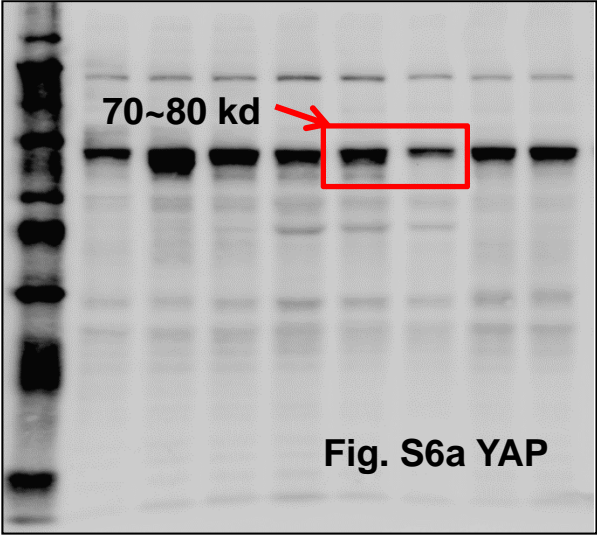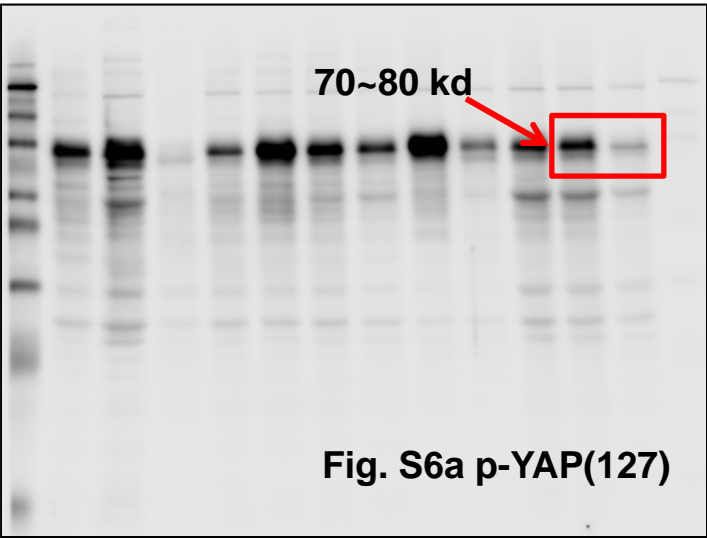

Supplement: Supplementary file 10 [file emmm0007-1426-sd10.zip › Source_data_for_Expanded_View_and_Appendix/Source_Data_for_Appendix_figure_S6.pdf]

Source Data for Expanded View

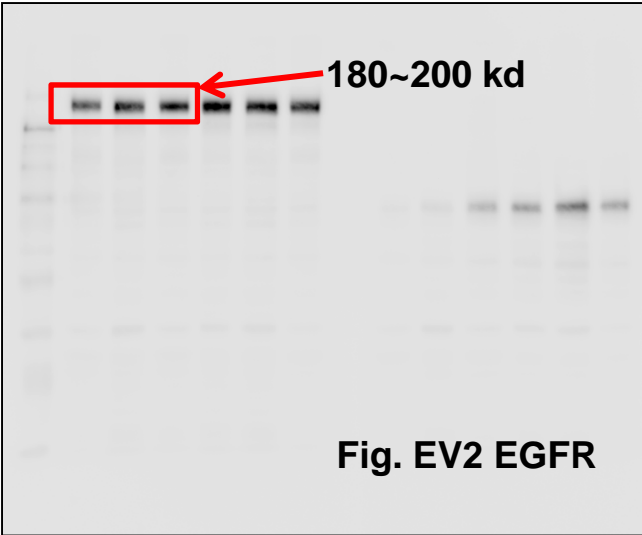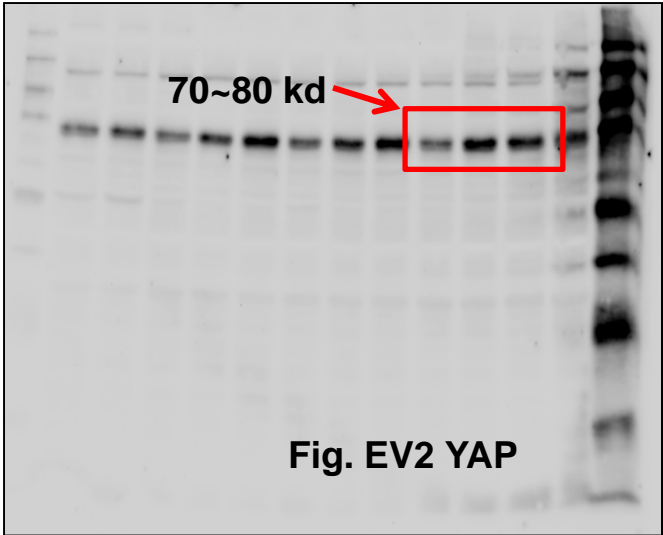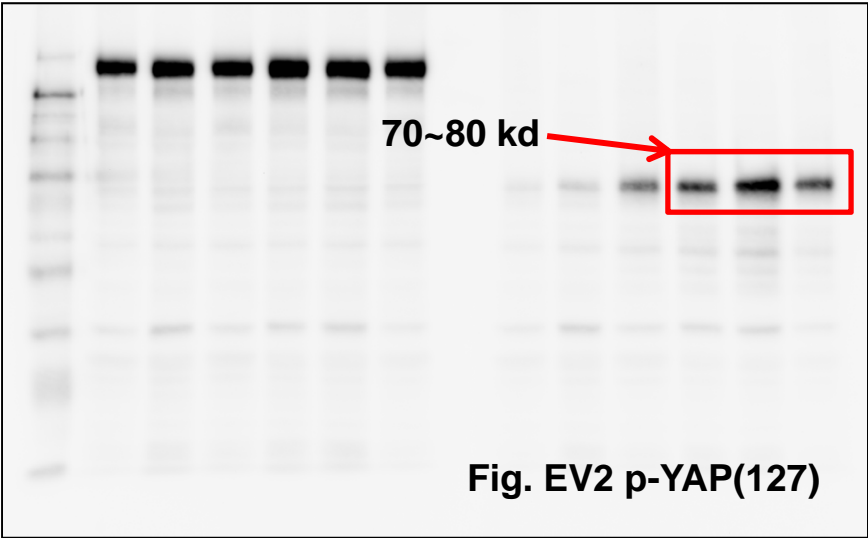

Supplement: Supplementary file 10 [file emmm0007-1426-sd10.zip › Source_data_for_Expanded_View_and_Appendix/Source_Data_Fig_EV2.pdf]
